# Solid-State Self-Assembly: Exclusive Formation and Dynamic Interconversion of Discrete Cyclic Assemblies Based on Molecular Tweezers

**DOI:** 10.1021/acs.joc.4c00794

**Published:** 2024-06-24

**Authors:** Koki Okabe, Masahiro Yamashina, Eiji Tsurumaki, Hidehiro Uekusa, Shinji Toyota

**Affiliations:** Department of Chemistry, School of Science, Tokyo Institute of Technology, 2-12-1 Ookayama, Meguro-ku, Tokyo 152-8551, Japan

## Abstract

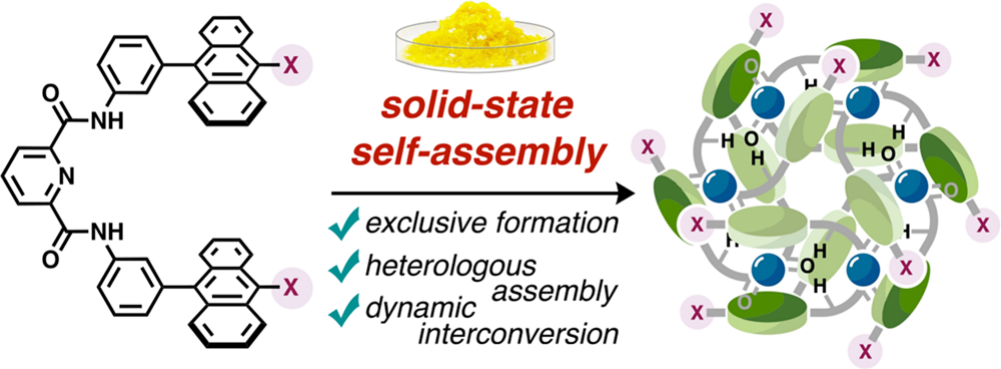

In contrast to self-assembly in solution systems, the construction of well-defined assemblies in the solid state has long been identified as a challenging task. Herein, we report the formation of tweezers-shaped molecules into various assemblies through a solid-state self-assembly strategy. The relatively flexible molecular tweezers undergo exclusive and quantitative assembly into either cyclic hexamers or a porous network through classical recrystallization or the exposure of powders to solvent vapor, despite the fact that they form only dimers in solution. The cyclic hexamers have high thermal stability and exhibit moderate solid-state fluorescence. The formation of heterologous assemblies consisting of different tweezers allows for tuning these solid-state properties of the cyclic hexamer. Furthermore, (trimethylsilyl)ethynyl-substituted tweezers demonstrate solvent-vapor-induced dynamic interconversion between the cyclic hexamer and a pseudocyclic dimer in the solid state. This assembly behavior, which has been studied extensively in solution-based supramolecular chemistry, had not been accomplished in the solid state so far.

## Introduction

Supramolecular self-assembly is a promising method for constructing well-defined and well-ordered architectures,^1^ some of which exhibit molecular recognition,^2^ catalyst,^3^ container,^4^ and biomedical applications.^5^ The dynamic nature of noncovalent interactions and dynamic covalent bonds can endow such assemblies with further structural complexity^6−8^ and variability,^9,10^ reminiscent of protein folding, and virus capsids.^11^ Historically, these assemblies have been constructed in solution based on kinetic and thermodynamic control,^12,13^ and there are extremely few reports on the construction of discrete structures in solid systems (i.e., solid-state self-assembly). This is because ordinary organic molecules prefer to adopt simple stacking or network arrangements in the crystal.^14−18^ Moreover, owing to the rigidity and uniformity of crystalline materials, heterologous assemblies^7^ and dynamic structural transformations,^9,10^ which are commonly observed in solution systems, are challenging to achieve in the solid state.

Despite these challenges, solid-state self-assembly^19,20^ has the potential to generate unique stable/metastable assemblies that differ from those in solution due to the lack of solvation and the availability of packing forces and mechanochemical stimuli. The term “solid-state self-assembly” encompasses two meanings here: (i) the construction of assemblies through crystallization (from solution to the solid state) and (ii) self-assembly in the solid state (from the solid state to the solid state), similar to solid-state synthesis.^21−24^ Indeed, some macrocyclic organic molecules coincidentally form unique cyclic or capsule-shaped assemblies such as dimers,^20,25,26^ trimers,^27^ and hexamers^19,28−30^ through crystallization. Recently, a few energetically unfavored coordination-driven self-assemblies have been constructed in the solid state via crystallization^31−34^ or under ball-milling conditions.^35−38^ As shown in these representative studies, the resulting self-assembled structures are essentially inaccessible in solution. Thus, solid-state self-assembly beyond typical crystal engineering^14,15^ represents an unconventional strategy that can potentially lead to the development of sophisticated well-defined supramolecules with unusual functions.

**Figure 1:**
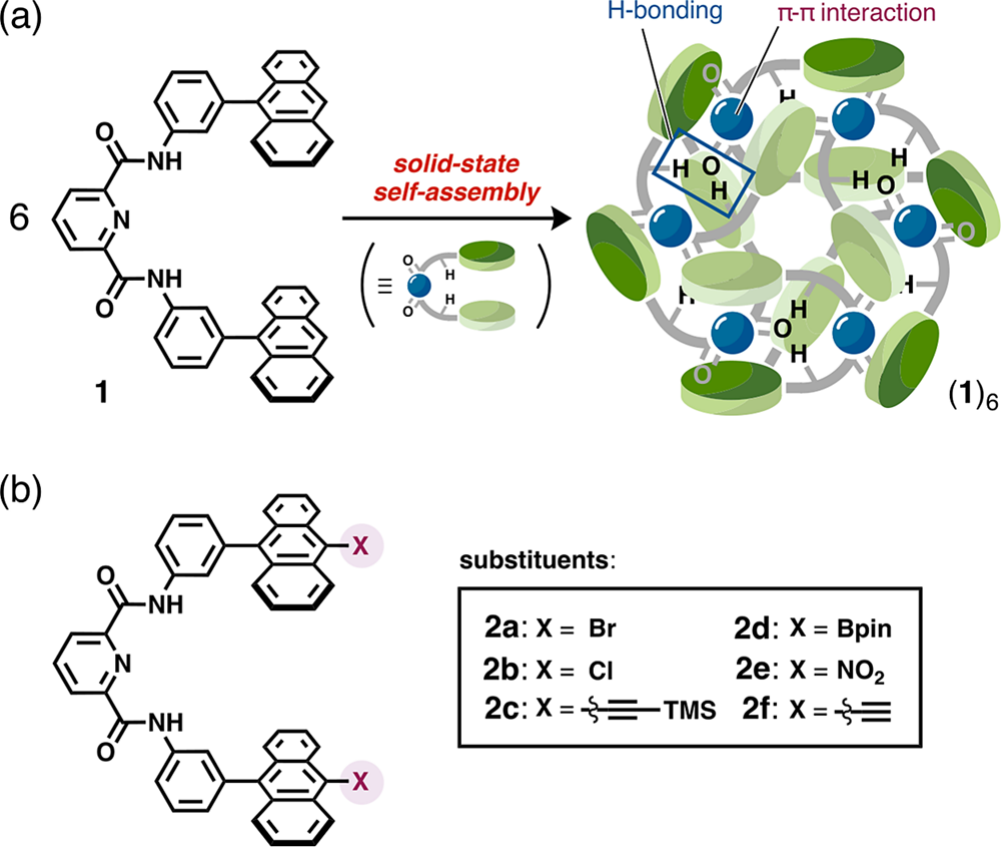
Conceptual illustration of the design and solid-state self-assembly of **1** and **2a–2f.** (a) Schematic representation of the solid-state self-assembly of molecular tweezers **1** based on self-complementary hydrogen bonding and π–π interactions. (b) Chemical structures of the substituted molecular tweezers **2** designed in this work.

For the investigation of the solid-state self-assembly, we took inspiration from anthracene-based molecular tweezers **1**, which possess a pyridinedicarboxamide (PDA) moiety (Figure 1a).^39^ Although the U-shaped tweezers molecules of **1** spontaneously form a dimer assembly ((**1**)_2_) in CHCl_3_, they assemble exclusively into a cyclic hexamer ((**1**)_6_) during crystallization (Figure 1a). A dual-interaction system with cooperative self-complementary hydrogen bonds and π–π interactions between molecules of **1** plays a key role in forming (**1**)_6_, as do crystalline packing forces. The cyclic hexamer not only exhibits solid-state fluorescence and high thermal stability but also shows hierarchical assembly to give a large rhombohedral structure in the presence of acids. Generally, rational construction strategies provide interior or exterior functionalized assemblies from substituted monomers.^13,40−43^ While the dual-interaction system observed for such molecular tweezers can be expected to represent a method to obtain metal-free, complex assemblies using intermolecular interactions,^44−46^ this type of cyclic hexamer has not been reported for systems other than tweezers **1**, which feature nonsubstituted anthracenes.

To demonstrate the solid-state self-assembly of organic molecules, we herein synthesize 10-substituted 9-anthryl molecular tweezers with bromo (**2a**), chloro (**2b**), (trimethylsilyl)ethynyl (TMSE; **2c**), boronic pinacol ester (Bpin; **2d**), nitro (**2e**), and ethynyl (**2f**) groups. The relatively flexible tweezers **2a**–**2f** assemble exclusively and quantitatively into either a cyclic hexamer with a diameter of up to ∼30 Å or a porous network with pores of ∼10–17 Å, depending on the substituents. These solid-state self-assemblies can be obtained using classical recrystallization techniques or by simply exposing powder samples to solvent vapor. The cyclic hexamers have high thermal stability and exhibit moderate solid-state fluorescence, the color of which can be tuned by the formation of heterologous assemblies composed of different tweezers. Furthermore, TMSE-substituted tweezers **2c** demonstrate dynamic interconversion between the cyclic hexamer ((**2c**)_6_) and the pseudocyclic dimer ((**2c**)_2_) triggered by solvent vapor, even in the crystalline powder.

## Results and Discussion

### Formation of Cyclic Hexamers Through the Solid-State Self-Assembly

**Figure 2:**
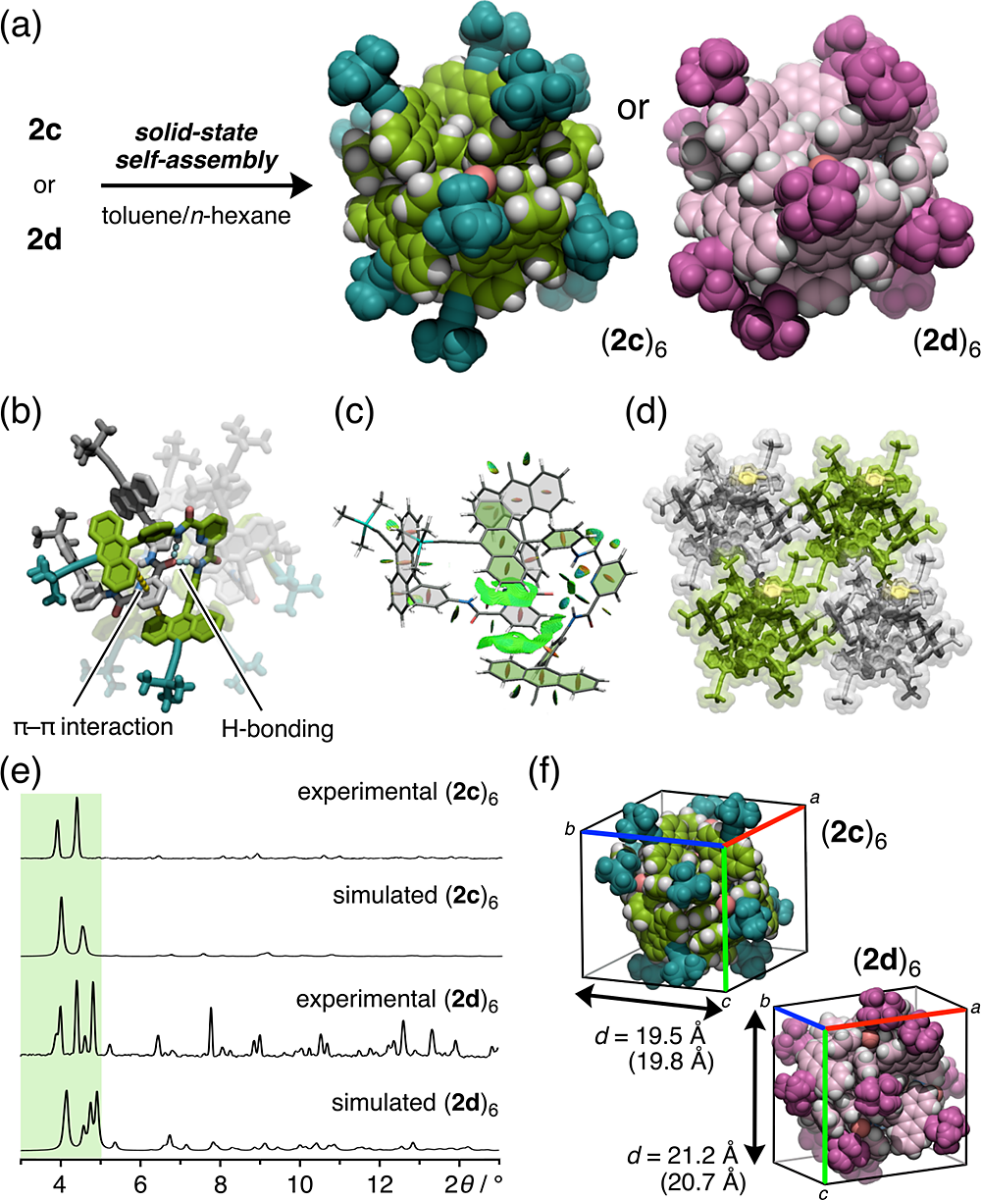
Formation and characterization of cyclic hexamers (**2c**)_6_ and (**2d**)_6_. (a) Formation of cyclic hexamers (**2c**)_6_ and (**2d**)_6_ from molecular tweezers **2c** and **2d**, respectively. In the X-ray crystal structures of (**2c**)_6_ and (**2d**)_6_, the substituted TMSE and Bpin groups are colored in dark green and pink, respectively. (b) Highlighted intramolecular hydrogen bonds (yellow dotted lines) and π–π interactions (green dotted lines) in (**2c**)_6_. (c) NCI plot of a single unit of the head-to-tail complex obtained from DFT calculations at the B3LYP-D3/6-31G(d,p) level (light green isosurface: π–π interactions; light blue isosurface: hydrogen bonds). (d) Crystal packing structure of (**2c**)_6_. For clarity, adjacent hexamers are shown in different colors in stick and transparent CPK representation. Toluene molecules are colored in yellow. (e) PXRD patterns of crystalline powders of (**2c**)_6_ and (**2d**)_6_, along with their simulated patterns based on the SXRD data. (f) Extracted unit cells of cyclic hexamers (**2c**)_6_ and (**2d**)_6_, showing the lattice spacing *d* and the corresponding molecular length between terminal C atoms (in parentheses).

A series of substituted molecular tweezers (**2**) were sequentially synthesized from 9-(3-nitrophenyl)anthracene^39^ (Figure S1). The obtained tweezers **2** form dimeric assemblies in CHCl_3_ with self-association constants (*K*_a_) of ∼130–330 M^–1^ (Figures S2 and S3). Owing to the steric repulsion, **2c** showed a smaller *K*_a_ value than **2a**. Unlike nonsubstituted tweezers **1**, TMSE-substituted tweezers **2c** do not crystallize in CH_2_Cl_2_/*n*-hexane. After exploring various combinations of solvents, we found that the slow diffusion of *n*-hexane into a toluene solution of **2c** affords suitable block-shaped single crystals in excellent yield (∼81%) within 12 h at room temperature. A single-crystal X-ray diffraction (SXRD) analysis unambiguously revealed that in this single crystal, six molecules of **2c** form a precise cyclic hexamer ((**2c**)_6_) with a width of ∼30 Å and a height of 27 Å (Figures 2a and S4). Interestingly, (**2c**)_6_ exhibits a dual-interaction system with global intermolecular hydrogen bonds between the carbonyl oxygen atoms and the amide hydrogen atoms, as well as π–π interactions between the anthracene and pyridine moieties (Figure 2b). These interactions are well supported by a noncovalent interaction (NCI) plot (Figure 2c). In the case of **2d** (X = Bpin), despite the bulky substituents on the anthracene moieties, hexamer (**2d**)_6_ is formed in the same manner (Figures 2a and S5). Due to the presence of Bpin groups, (**2d**)_6_ is considerably larger (width: 29 Å; height: 24 Å; molecular weight: 5530.26) than nonsubstituted hexamer (**1**)_6_. Focusing on the crystal packing structure, both (**2c**)_6_ and (**2d**)_6_ adopt a slip-stacked-like arrangement, in which some of the elongated substituent groups mutually interact with concavities of the adjacent hexamers via toluene molecules (Figures 2d, S4, and S5). Moreover, multiple intermolecular CH−π and van der Waals interactions were observed between adjacent hexamers in the crystal.

The experimental powder X-ray diffraction (PXRD) patterns of (**2c**)_6_ and (**2d**)_6_ were consistent with the simulated PXRD patterns based on the corresponding SXRD data (Figure 2e). This result indicates that the obtained powders are single phase and predominately consist of the cyclic hexamers. Interestingly, cyclic hexamers (**2c**)_6_ and (**2d**)_6_ were also generated upon exposure of their amorphous powders to toluene vapor (Figures S7 and S8). Thus, the solid-state self-assembly method can provide supramolecular assemblies directly from the corresponding amorphous powders. The simulated patterns showed characteristic low-angle diffractions for Miller index (100), (010), or (001) at ∼4° (2θ), indicating long crystal periodicity with a lattice spacing *d* of ∼22 Å as estimated using Bragg’s law. Since each lattice cell is occupied by one hexameric assembly, the observed lattice spacing *d* is comparable to the size of one hexamer (∼20 Å; Figure 2f). Thus, some low-angle diffractions (2θ ≤ 5°) suggest the existence of hexameric assemblies in the crystalline powders. Although the preparation of single crystals of **2f** (X = −C≡CH) was unsuccessful, the presence of low-angle diffractions (2θ = 4.7–4.9°; *d* = 18–19 Å) in the PXRD analysis also suggests the formation of cyclic hexamers (Figure S9).

### Formation of Porous Network Assemblies

**Figure 3:**
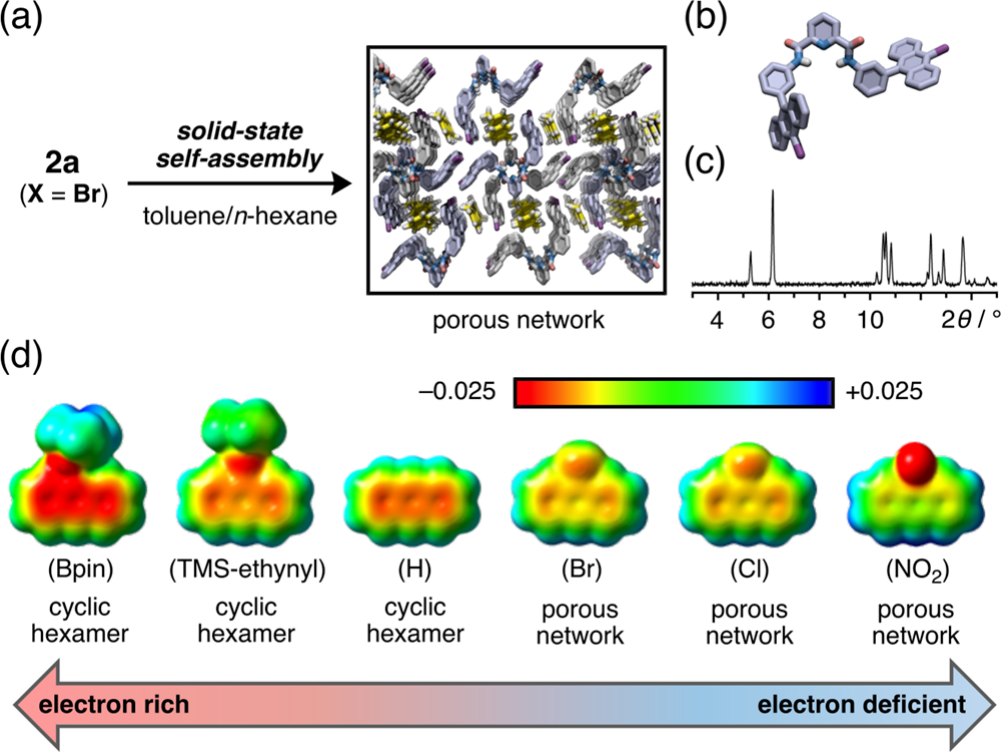
Formation of cyclic hexamers or porous network assemblies. (a) Formation of porous network assemblies by electron-deficient molecular tweezers **2a** (X = Br) and **2b** (X = Cl), as well as the crystal packing structure of **2a** (stick representation; toluene molecules are colored in yellow). (b) Conformation of **2a** in the crystal. (c) PXRD pattern of a crystalline powder sample of **2a**. (d) Relationship between the solid-state self-assembly mode and the electron density on the anthracene moieties. ESP maps of substituted anthracenes were obtained from DFT calculations at the B3LYP-D3/6-31G(d,p) level.

Interestingly, the PXRD pattern of the crystalline powder of **2a** (X = Br) obtained from a toluene/*n*-hexane solution was incongruous with that of cyclic hexamer (**2c**)_6_. In particular, the lowest-angle diffraction peak was observed at 2θ ≈ 5.3° (Figures 3c and S10). An SXRD analysis unambiguously revealed that tweezers **2a** form a porous network structure instead of cyclic hexamers due to the outward rotation of one of the anthracene arms (Figures 3a,b and S6). The network structure features multiple intermolecular π–π interactions (anthracene–anthracene and anthracene–toluene–anthracene) and H-bonds in a one-dimensional array, leading to the formation of an open-pore channel structure, in which two toluene molecules are accommodated within a cavity. Similar PXRD patterns were recorded for **2b** (X = Cl) and **2e** (X = NO_2_) (Figure S11). Notably, **2a**, **2b**, and **2e** all feature electron-withdrawing substituents, and their electrostatic potential (ESP) maps clearly indicate a remarkable decrease in electron density on the anthracene moiety (Figure 3d).^47^ Thus, these results suggest that the molecular tweezers exhibit switchable assembly behavior based on modulation of the electron density on the anthracenes moieties, possibly via the suppression of the electrostatically driven π–π interactions between the anthracene and pyridine moieties. In contrast to the cyclic hexamers, the amorphous powders of **2a**, **2b**, and **2e** merely dissolve upon exposure to toluene vapor.

### Solid-State Optical and Physical Properties of the Cyclic Hexamer

**Figure 4:**
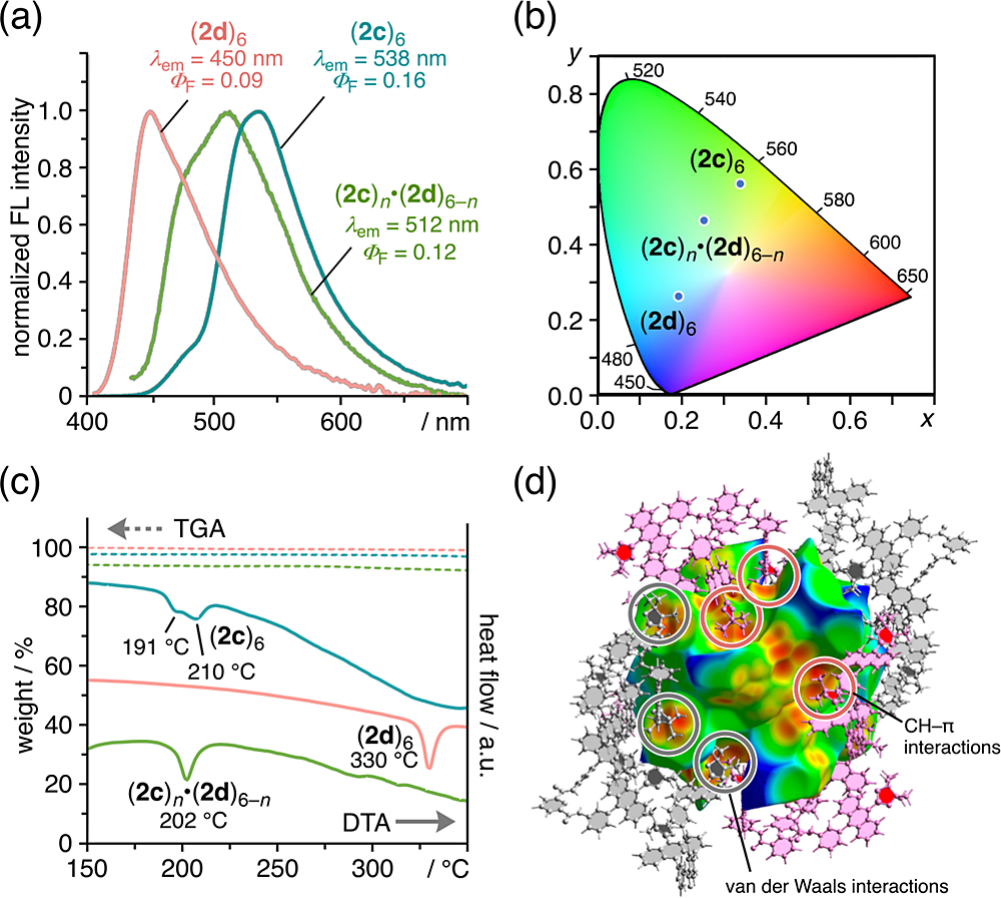
Optical and physical properties of the cyclic hexamers in the solid state. (a) Solid-state fluorescence spectra of the cyclic hexamers and (b) their CIE coordinate diagram. Excitation wavelength for each set of tweezers was set to its absorption maximum in CHCl_3_ at 298 K. (c) TG–DTA curves in the region 150–350 °C (dotted line: TGA; solid line: DTA). (d) Hirshfeld surface analysis of (**2d**)_6_, which illustrates the distance (*d*_e_) from the surface to the nucleus of the external atoms in adjacent molecules using a red–green–blue color scheme. Pink- and gray-colored tweezers engage in CH−π (Bpin–anthracene) and van der Waals (Bpin–Bpin) interactions with adjacent cyclic hexamers (**2d**)_6_, respectively (note: solvent molecules were omitted in the calculations).

We then investigated the solid-state fluorescence of these assemblies. Typically, the fluorescence of molecules tends to be quenched in the solid state compared to that in solution. Indeed, tweezers **2a** and **2b**, which contain halogen atoms, show very weak solid-state fluorescence (Figure S12). Tweezers **2e** was nonfluorescent both in a solution and the solid state, reflecting the nature of 9-nitroanthracene.^48^ However, hexamers (**2c**)_6_ and (**2d**)_6_ retain relatively high fluorescence in the solid state. The Bpin-functionalized hexamer (**2d**)_6_ shows blue emission (λ_em_ = 450 nm; Φ_F_ = 9%) similar to that of (**1**)_6_ (Figures 4a,b and S12). In contrast, hexamer (**2c**)_6_, which contains TMSE groups, exhibits characteristic bright yellowish green fluorescence (λ_em_ = 538 nm; Φ_F_ = 16%). The emission lifetime of (**2c**)_6_ was determined to be 3.1 ns (Figure S12); thus, this unique yellowish green fluorescence was attributed to the ethynyl-substituted anthracene.^49^

The thermal stability of the assemblies was evaluated via thermogravimetry–differential thermal analysis (TG–DTA) (Figures 4c and S13). For (**2c**)_6_, an endothermic trough due to the melting point (i.e., the disassembly temperature) appeared at ∼210 °C, similar to that observed for nonsubstituted hexamer (**1**)_6_. It should be noted here that (**2d**)_6_ displays significantly high thermal stability (330 °C). In the crystal packing of (**2d**)_6_, some of the methyl groups in the substituents interact with adjacent hexamers via CH−π (Bpin–anthracene) and van der Waals (Bpin–Bpin) interactions. A Hirshfeld surface analysis using the *d*_e_ map clearly revealed the presence of close Bpin–anthracene and Bpin–Bpin contacts (Figure 4d), leading to an increase in the total amount of H–H and H–C contacts (Figure S14). Therefore, the high thermal stability of crystals of (**2c**)_6_ and (**2d**)_6_ might be due to the multiple intermolecular interactions between their exterior substituents.

### Construction of Heterologous Cyclic Hexamers

**Figure 5:**
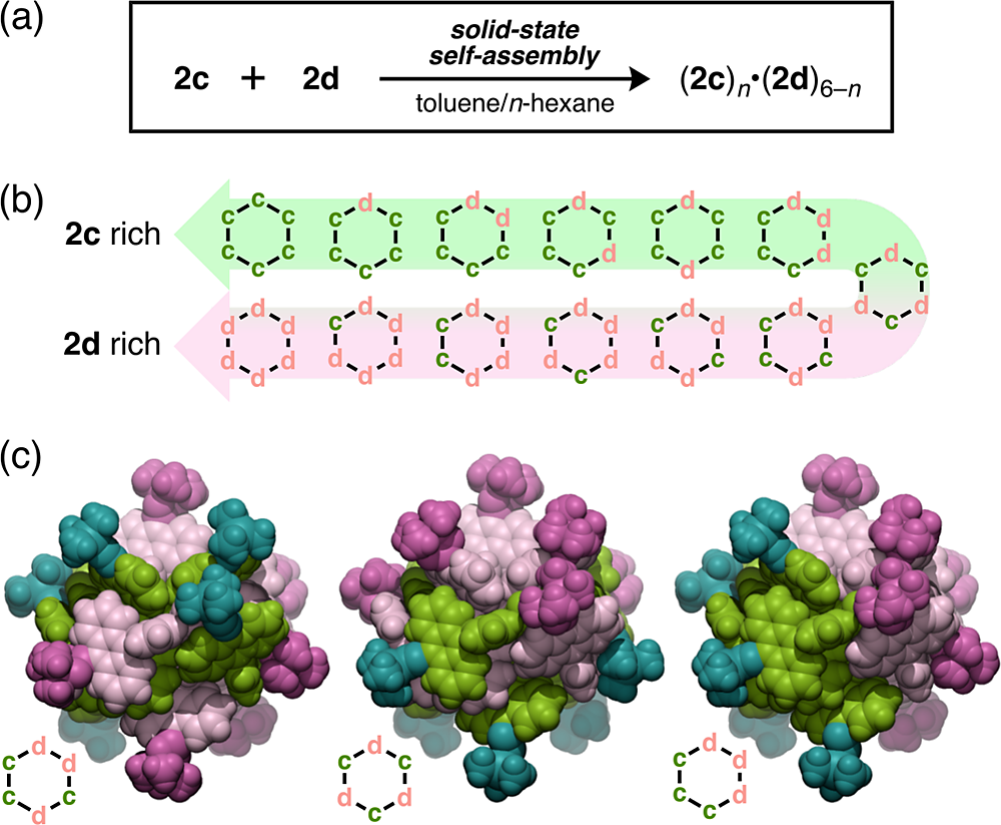
Construction of heterologous cyclic hexamers of the type (**2c**)_*n*_·(**2d**)_6–_*_n_* (*n* = 0–6) in the solid state. (a) Construction of heterologous cyclic hexamers (**2c**)_*n*_·(**2d**)_6–*n*_ (*n* = 0–6) through cocrystallization from a 1:1 mixture of **2c** and **2d**. (b) Schematic representation of the 13 possible different kinds of heterologous assemblies that can be formed. Letters c and d in the hexagons indicate tweezers **2c** and **2d**, respectively. (c) CPK representations of heterologous cyclic hexamers consisting of **2c** and **2d** in 1:1 ratio, based on the SXRD analysis. Molecules of **2c** and **2d** are colored in green and pink, respectively.

We next investigated the solid-state self-assembly of a mixture of different tweezers. When a mixture of **2c** and **2d** (1:1 molar ratio) was recrystallized from a toluene/*n*-hexane solution, yellow block-shaped single crystals were successfully obtained. The resultant crystals were found to have relatively high stability compared to those of (**2c**)_6_, which were fragile in various oils for crystal mount. Surprisingly, an SXRD analysis revealed cyclic hexamer assemblies in which **2c** and **2d** were randomly arranged (Figures 5a,b and S15). The occupancy of the disordered **2c** and **2d** in the cyclic hexamers was estimated to be ∼50%, which is in good agreement with the ratio of the ^1^H NMR signals (**2c**:**2d** = 1:1) of the cocrystals in CDCl_3_ (Figure S16). We then prepared cocrystals with different loading ratios of **2c** and **2d**, and the ratios of the two tweezers in the cocrystals were comparable to the loading ratios (as determined from ^1^H NMR measurements) (Figure S16). These results suggest that the combination of **2c** and **2d** likely results in the formation of 13 kinds of heterologous cyclic hexamers, (**2c**)_*n*_·(**2d**)_6–*n*_ (*n* = 0–6, Figures 5b,c and S17), and that these are similar to mixed crystals^50^ or nonstoichiometric cocrystals.^51^ As shown in Figure 5c, a 1:1 mixture of **2c** and **2d** provides three feasible conformers of heterologous assemblies, despite the different substituents. Comparing this behavior with the self-sorting observed for mixtures of **1** and various PDA-based compounds,^39^ we concluded that the cocrystallization of molecular tweezers **2c** and **2d** stems from their structural similarity. The PXRD pattern of (**2c**)_*n*_·(**2d**)_6–*n*_ was slightly different than those of (**2c**)_6_ and (**2d**)_6_ (Figure S18). The crystalline powders of (**2c**)_*n*_·(**2d**)_6–*n*_ displayed yellowish green emission with a color intermediate between that of (**2c**)_6_ and (**2d**)_6_ (Φ_F_ = 12%, λ_em_ = 512 nm, Figure 4a,b); however, the fluorescence spectrum was not simply a combination of those of (**2c**)_6_ and (**2d**)_6_ (Figure S19). This result may be attributed to the perturbation between **2c** and **2d** in proximity within the cyclic hexamer. In contrast to the change in fluorescence, the disassembly temperature of (**2c**)_*n*_·(**2d**)_6–*n*_ was 202 °C, which was similar to that of (**2c**)_6_ (Figures 4c and S20).

### Dynamic Interconversion Between Assemblies in the Solid State

**Figure 6:**
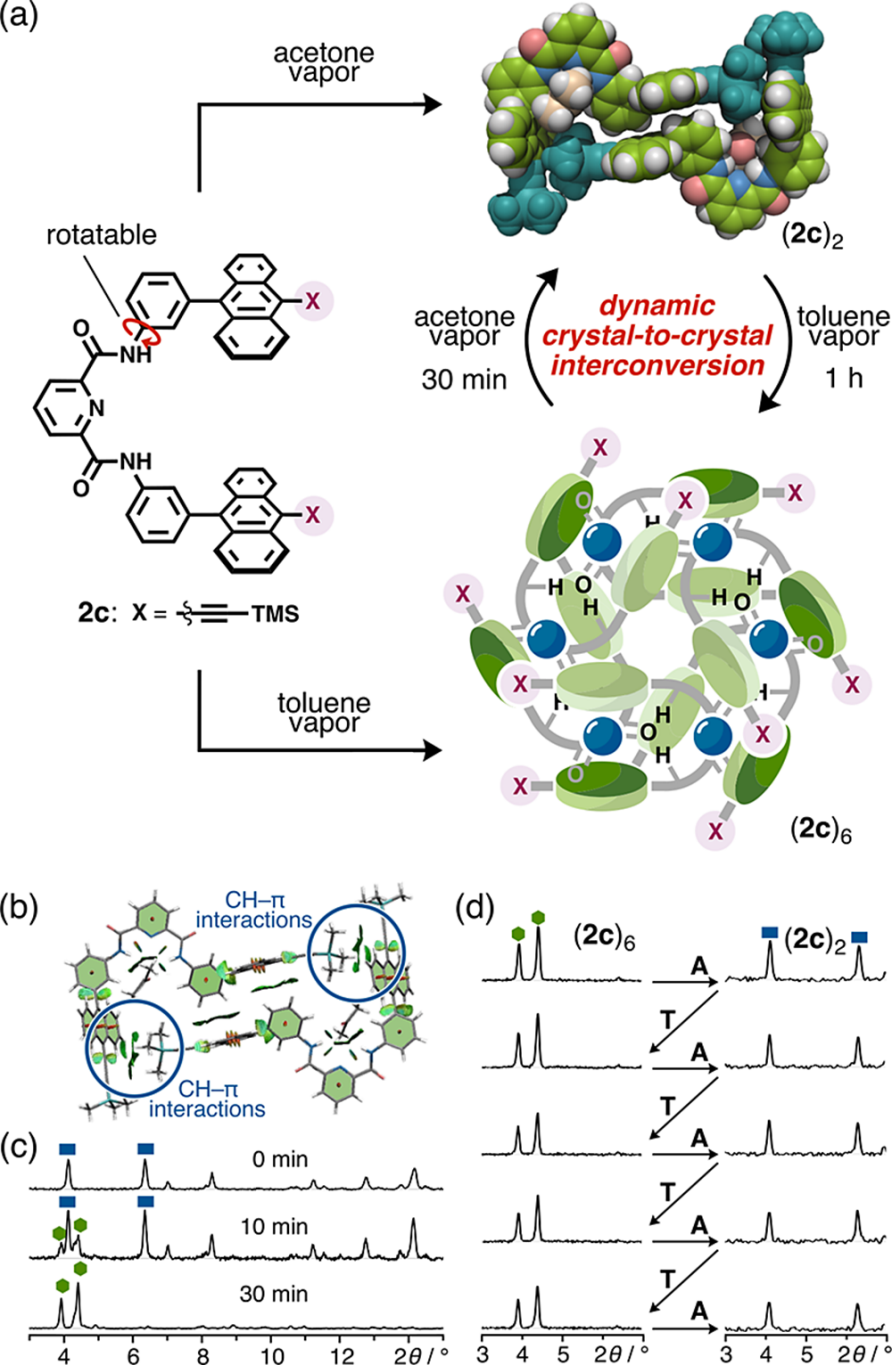
Dynamic interconversion between (**2c**)_6_ and (**2c**)_2_ in the solid state. (a) Schematic representation of the formation of cyclic hexamer (**2c**)_6_ or pseudodimer (**2c**)_2_ controlled by the choice of solvent vapor and solvent vapor-induced dynamic interconversion between the two types of assemblies in the crystalline powders. X-ray crystal structure of (**2c**)_2_ is presented as a CPK representation. TMSE groups and acetone molecules are colored in dark green and yellow, respectively. (b) NCI plot of (**2c**)_2_–acetone adducts obtained from DFT calculations at the B3LYP-D3/6-31G(d,p) level (light green isosurface: π–π interactions; light blue isosurface: hydrogen bonds). (c) Time-course PXRD measurements of (**2c**)_2_ under a toluene vapor atmosphere at 298 K (blue rectangles: (**2c**)_2_; green hexagons: (**2c**)_6_). (d) Tests of repeated crystal-to-crystal interconversion between (**2c**)_6_ and (**2c**)_2_ at 298 K. Crystalline powders were exposed to acetone vapor for 30 min (**A**) or toluene vapor for 1 h (**T**), and the progress of interconversion at each step was confirmed using PXRD.

The aforementioned results allowed us to design other assemblies using molecular tweezers. After careful examination of the crystallization conditions, we developed the idea of adding a polar solvent as an additive. The crystallization of **2c** in toluene/*n*-hexane in the presence of a small amount of acetone led to the formation of pseudocyclic dimer (**2c**)_2_–acetone adducts instead of cyclic hexamer (**2c**)_6_, as confirmed by SXRD analysis (Figures 6a, S21, and S22). Rectangular-shaped cyclic dimer (**2c**)_2_ has a molecular weight of 1724.38 and a size of ∼26 Å × 19 Å. While one anthracene arm of **2c** is inverted, as in the porous network (**2a**)_*n*_, the inner space is completely closed due to the complementary CH−π interactions between the TMS and anthracene moieties. The amide PDA moiety engages in hydrogen bonding to a molecule of acetone; this inhibits intermolecular hydrogen bonding between tweezers, which is important for the construction of cyclic hexamers (i.e., the dual-interaction system). These intermolecular interactions are also reflected in the NCI plot (Figure 6b). Cyclic dimer (**2c**)_2_ displays yellowish green solid-state fluorescence (Φ_F_ = 13%) and relatively high thermal stability (206 °C), similar to that of hexamer (**2c**)_6_ (Figures S23 and S24). Other molecular tweezers, such as **1** and **2d**, did not yield single crystals under these conditions, implying that the CH−π TMS–anthracene interaction may play a key role in forming this characteristic cyclic dimer.

Having observed the additive-induced change in the assembly behavior, we finally attempted an interconversion between the cyclic hexamer and dimer via crystal-to-crystal transformation. First, we found that pseudocyclic dimer (**2c**)_2_ could also be obtained via the solid-state self-assembly method by exposing amorphous powder **2c** to acetone vapor (Figure S22). Surprisingly, upon exposure to toluene vapor for 1 h at room temperature, the crystalline powder of (**2c**)_2_ was quantitatively transformed into cyclic hexamer (**2c**)_6_ (Figure 6a,b). A time-course PXRD analysis indicated a gradual increase in the characteristic low-angle diffractions of (**2c**)_6_ (3.9° and 4.4°) accompanied by a decrease in those of (**2c**)_2_ (4.1° and 6.3°) (Figure S25). Notably, when the obtained crystalline powder of (**2c**)_6_ was subsequently exposed to acetone vapor, the diffractions of (**2c**)_2_ were regenerated within 30 min (Figure S26). Thus, the solvent-triggered interconversion between the hexamer and the dimer is reversible. This interconversion essentially proceeds by local recrystallization induced by solvent vapor from the surface to the interior. In a typical solvent-vapor-induced crystal-to-crystal transformation,^52,53^ the molecular alignment pattern (i.e., crystallinity) only changes when solvent molecules are incorporated into the crystalline system. The present type of crystal-to-crystal transformation, which involves the interconversion between discrete assemblies accompanied by the dynamic structural transformation of the monomer molecules, has rarely been reported so far.

In addition, repeated exposure tests revealed that the interconversion progresses quantitatively, whereby the diffraction intensity of each assembly is fully recovered even after five cycles of alternating exposure to toluene and acetone vapor (Figures 6d and S27). The number of tweezers composing each (**2c**)_2_ assembly is smaller than that for (**2c**)_6_, and correspondingly, the dimers are formed faster (30 min) than the hexamers (1 h) as determined using the exposure time necessary for transformation. The assembly of tweezers **2c** was found to be highly sensitive to acetone. Competitive experiments indicated that cyclic dimer (**2c**)_2_ is formed selectively even under toluene vapor containing a small amount of acetone (toluene/acetone = 20/1, v/v) (Figure S28). Moreover, when single crystalline (**2c**)_2_ was subjected to the interconversion, the single crystallinity was completely collapsed in the resulting (**2c**)_6_, whereas the transformation of single crystalline (**2c**)_6_ gave (**2c**)_2_ with relatively intact single crystallinity. The interconversion also proceeded in selected nonpolar solvents (e.g., benzene and *m*-xylene; Figure S29), while other solvents (e.g., *p*-xylene and mesitylene) did not induce any changes in the PXRD data (Figure S29). In contrast, due to low vapor pressure, the interconversion did not proceed in DMF and DMSO solvents containing C=O and S=O groups, respectively. These results indicate that efficient crystal-to-crystal conversion requires stabilization of the packing structure of each crystal through substituents and crystalline solvents.

## Conclusions

In summary, we have successfully demonstrated solid-state self-assembly with the exclusive formation of and interconversion between two types of supramolecular assemblies in the crystalline state. Molecular tweezers that contain 10-substituted anthracenes (**2a**–**2f**) form either cyclic hexamers or a porous network, depending on the electron density of the anthracene moieties, as determined using single-crystal and powder X-ray diffraction analyses. Interestingly, the construction of homologous and heterologous cyclic hexamers was successfully achieved by cocrystallization of two different molecular tweezers. These cyclic hexamers displayed blue, green, and yellowish green solid-state fluorescence, which reflects the properties of the substituents, as well as high thermal stability. Furthermore, the tweezers that contain TMSE groups showed quantitative interconversion between a self-complementary cyclic hexamer and a pseudodimer involving dynamic structural transformation in the solid state. These results indicate that controlling π–π interactions and hydrogen bonding in flexible PDA-based molecular tweezers has a potential to exhibit various assembly behavior in the solid state. Previous studies on organic frameworks (e.g., COFs^54^ and HOFs^17,18^) have demonstrated that crystals with specifically aligned organic molecules exhibit high functionality. We thus plan to further investigate the structural diversity of supramolecules through solid-state self-assembly, which will open the door to a deeper understanding of solid-state supramolecular chemistry and the design of metal-free functional nanomaterials.

## Experimental Section

### Synthesis of **2a**–**2e**

2,6-Pyridinedicarbonyl dichloride (51.6 mg, 0.253 mmol), **4a** (0.185 g, 0.531 mmol), and anhydrous CH_2_Cl_2_ (3 mL) were added to a 10 mL of reaction vial with a screw cap under N_2_. After addition of triethylamine (71 μL, 0.51 mmol), the solution was stirred for 3 h at room temperature. The resultant mixture was diluted with CH_2_Cl_2_ and washed with 10% HCl aq.,10% NaOH aq., and brine, respectively. The combined organic layer was dried over Na_2_SO_4_, filtrated, and concentrated under reduced pressure. The obtained solid was purified by column chromatography on silica gel (CH_2_Cl_2_) to afford **2a** as an orange solid (199 mg, 95%). Under the same procedure, **2b** (157 mg, 95% yield, a pale yellow solid), **2c** (143 mg, 88% yield, a yellow solid), **2d** (382 mg, 94% yield, a pale yellow solid), and **2e** (438 mg, 97% yield, an orange solid) were synthesized from **4b** (490 mg, 1.61 mmol), **4c** (140 mg, 0.383 mmol), **4d** (360 mg, 0.911 mmol), and **4e** (393 mg, 1.25 mmol), respectively.

for **2a**: ^1^H NMR (500 MHz, CDCl_3_, 298 K, 37 mM): δ 9.11 (s, 2H), 8.46 (d, *J* = 9.1 Hz, 4H), 7.78 (d, *J* = 7.4 Hz, 2H), 7.69 (d, *J* = 7.4 Hz, 2H), 7.51 (s, 2H), 7.47 (t, *J* = 7.4 Hz, 4H), 7.43 (d, *J* = 9.1 Hz, 4H), 7.40 (t, *J* = 7.4 Hz, 2H), 7.23 (br, 1H), 7.18 (t, *J* = 7.4 Hz, 4H), 7.00 (d, *J* = 7.4 Hz, 2H); ^13^C{^1^H} NMR (125 MHz, CDCl_3_, 298 K): δ 161.4 (C=O), 147.7 (C_*q*_), 139.4 (C_*q*_), 138.3 (CH), 137.8 (C_*q*_), 137.1 (C_*q*_), 131.0 (C_*q*_), 130.4 (C_*q*_), 129.7 (CH), 128.4 (CH), 127.9 (CH), 127.5 (CH), 127.4 (CH), 126.1 (CH), 125.3 (CH), 123.4 (C_*q*_), 122.7 (CH), 120.2 (CH); HRMS (ESI-TOF, CHCl_3_/CH_3_OH) *m*/*z*: [M + Na]^+^ calcd for C_47_H_29_^79^Br_2_N_3_O_2_Na, 850.0504; found 850.0489; mp = 193–195 °C.

for **2b**: ^1^H NMR (500 MHz, CDCl_3_, 298 K, 38 mM): δ 9.13 (s, 2H), 8.43 (d, *J* = 9.1 Hz, 4H), 7.80 (d, *J* = 7.4 Hz, 2H), 7.72 (d, *J* = 7.4 Hz, 2H), 7.52 (s, 2H), 7.48 (t, *J* = 9.1 Hz, 4H), 7.44 (d, *J* = 9.1 Hz, 4H), 7.41 (t, *J* = 7.9 Hz, 2H), 7.25 (t, *J* = 7.4 Hz, 1H), 7.20 (dd, *J* = 9.1, 7.4 Hz, 4H), 7.00 (d, *J* = 7.4 Hz, 2H); ^13^C{^1^H} NMR (125 MHz, CDCl_3_, 298 K): δ 161.4 (C=O), 147.8 (C_*q*_), 139.3 (C_*q*_), 138.3 (CH), 137.8 (C_*q*_), 136.1 (C_*q*_), 130.7 (C_*q*_), 129.7 (CH), 129.2 (C_*q*_), 128.7 (C_*q*_), 128.0 (CH), 127.3 (CH), 127.1 (CH), 126.1 (CH), 125.4 (CH), 125.3 (CH), 122.8 (CH), 120.2 (CH); HRMS (ESI-TOF, CHCl_3_/CH_3_OH) *m*/*z*: [M + Na]^+^ calcd for C_47_H_29_^35^Cl_2_N_3_O_2_Na, 760.1529; found 760.1527; mp = 170–174 °C.

for **2c**: ^1^H NMR (500 MHz, CDCl_3_, 298 K, 28 mM): δ 9.29 (s, 2H), 8.55 (d, *J* = 8.5 Hz, 4H), 7.90 (d, *J* = 7.9 Hz, 2H), 7.76 (d, *J* = 7.9 Hz, 2H), 7.63 (s, 2H), 7.48–7.56 (m, 8H), 7.45 (t, *J* = 7.9 Hz, 2H), 7.36 (t, *J* = 7.9 Hz, 1H), 7.25 (t, *J* = 7.9 Hz, 4H), 7.06 (d, *J* = 7.4 Hz, 2H), 0.47 (s, 18H); ^13^C{^1^H} NMR (125 MHz, CDCl_3_, 298 K): δ 161.0 (C=O), 147.7 (C_*q*_), 139.3 (C_*q*_), 138.3 (CH), 137.5 (C_*q*_), 137.4 (C_*q*_), 132.4 (C_*q*_), 129.5 (C_*q*_), 129.2 (CH), 127.6 (CH), 127.1 (CH), 127.0 (CH), 126.5 (CH), 125.8 (CH), 125.1 (CH), 122.6 (CH), 119.8 (CH), 117.6 (C_*q*_), 107.0 (C_*q*_), 101.7 (C_*q*_), 0.5 (CH_3_); HRMS (ESI-TOF, CHCl_3_/CH_3_OH) *m*/*z*: [M + Na]^+^ calcd for C_57_H_47_N_3_O_2_Si_2_Na, 884.3099; found 884.3080; mp = 180–184 °C.

for **2d**: ^1^H NMR (500 MHz, CDCl_3_, 298 K, 34 mM): δ 9.42 (s, 2H), 8.36 (d, *J* = 8.5 Hz, 4H), 7.86 (d, *J* = 7.4 Hz, 2H), 7.75 (d, *J* = 7.7 Hz, 2H), 7.67 (s, 2H), 7.50 (d, *J* = 8.5 Hz, 4H), 7.44 (t, *J* = 7.9 Hz, 2H), 7.40 (t, *J* = 7.9 Hz, 4H), 7.29 (t, *J* = 7.4 Hz, 1H), 7.21 (t, *J* = 7.9 Hz, 4H), 7.03 (d, *J* = 7.4 Hz, 2H), 1.62 (s, 24H); ^13^C{^1^H} NMR (125 MHz, CDCl_3_, 298 K): δ 161.7 (C=O), 148.1 (C_*q*_), 140.4 (C_*q*_), 139.0 (C_*q*_), 138.9 (CH), 137.9 (C_*q*_), 135.7 (C_*q*_), 129.9 (C_*q*_), 129.6 (CH), 129.0 (CH), 128.1 (CH), 127.6 (CH), 126.0 (CH), 125.5 (CH), 125.5 (CH), 123.0 (CH), 120.1 (CH), 85.1 (C_*q*_), 25.9 (CH_3_). One aromatic C_*q*_ signal is significantly broadened; HRMS (ESI-TOF, CHCl_3_/CH_3_OH) *m*/*z*: [M + Na]^+^ calcd for C_59_H_53_^11^B_2_N_3_O_6_Na, 944.4013; found 944.4040; mp = 227–231 °C.

for **2e**: ^1^H NMR (500 MHz, CDCl_3_, 298 K, 26 mM): δ 9.30 (s, 2H), 7.91 (d, *J* = 7.9 Hz, 2H), 7.85 (d, *J* = 9.1 Hz, 4H), 7.69 (d, *J* = 7.9 Hz, 2H), 7.62 (s, 2H), 7.55 (t, *J* = 9.1 Hz, 4H), 7.52 (d, *J* = 9.1 Hz, 4H), 7.44 (t, *J* = 7.4 Hz, 2H), 7.42 (br, 1H), 7.27 (t, *J* = 9.1 Hz, 4H), 7.04 (d, *J* = 7.4 Hz, 2H); ^13^C{^1^H} NMR (125 MHz, CDCl_3_, 298 K): δ 161.0 (C=O), 147.5 (C_*q*_), 144.5 (C_*q*_), 140.1 (C_*q*_), 138.6 (CH), 138.1 (C_*q*_), 137.4 (C_*q*_), 129.6 (C_*q*_), 129.3 (CH), 128.7 (CH), 127.3 (CH), 126.8 (CH), 126.3 (CH), 125.3 (CH), 122.1 (CH), 122.0 (C_*q*_), 121.3 (CH), 120.1 (CH); HRMS (ESI-TOF, CH_3_CN/CH_3_OH) *m*/*z*: [M + Na]^+^ calcd for C_47_H_29_N_5_O_6_Na, 782.2010; found 782.1996; mp = 212–215 °C.

### Synthesis of **2f**

Compound **2c** (15.0 mg, 0.017 mmol) and THF (3 mL) were added to a 10 mL flask. After addition of a 1.0 M *n*-Bu_4_NF solution in THF (40 μL, 0.040 mmol), the solution was stirred for 1 h at room temperature. The resultant mixture was diluted with AcOEt and washed with 10% NaOH aq. and brine, respectively. The combined organic layer was dried over Na_2_SO_4_, filtrated, and concentrated under reduced pressure. The crude product was washed with methanol to afford **2f** as a brown solid (10.4 mg, 83%).

^1^H NMR (500 MHz, CDCl_3_, 298 K, 58 mM): δ 9.05 (s, 2H), 8.51 (d, *J* = 8.2 Hz, 4H), 7.79 (d, *J* = 7.9 Hz, 2H), 7.68 (d, *J* = 7.9 Hz, 2H), 7.55 (s, 2H), 7.47–7.44 (m, 8H), 7.40 (t, *J* = 7.9 Hz, 2H), 7.24 (t, *J* = 7.4 Hz, 1H), 7.19 (t, *J* = 7.9 Hz, 4H), 7.00 (d, *J* = 7.7 Hz, 2H), 3.98 (s, 2H);^13^C{^1^H} NMR (125 MHz, CDCl_3_, 298 K): δ 160.9 (C=O), 147.2 (C_*q*_), 138.9 (C_*q*_), 137.9 (CH), 137.8 (C_*q*_), 137.4 (C_*q*_), 132.6 (C_*q*_), 129.3 (C_*q*_), 129.2 (CH), 127.3 (CH), 127.0 (CH), 126.8 (CH), 126.6 (CH), 125.7 (CH), 124.8 (CH), 122.2 (CH), 119.7 (CH), 116.3 (C_*q*_), 89.0 (-*C*CH), 80.3 (-C*C*H); HRMS (FD) *m*/*z*: [M]^+^ calcd for C_51_H_31_N_3_O_2_, 717.2416; found 717.2437; mp = 190–195 °C (decomp.).

### Construction of Cyclic Hexamer

To a 50 mL round bottom flask, molecular tweezers **2c** (101 mg, 0.264 mmol) was dissolved in toluene (10 mL), and subsequently *n*-hexane (20 mL) was slowly layered on the solution. The flask was placed at room temperature. After 12 h, yellow crystals of cyclic hexamer (**2c**)_6_ were obtained (82.0 mg, 81%). Under the same procedure, pale yellow crystals of (**2d**)_6_ (86% yield) were prepared from **2d**.

for (**2c**)_6_: FT-IR (ATR, cm^–1^): 3311, 3061, 2957, 2898, 2143, 2119, 1689, 1662, 1607, 1533, 1490, 1438, 1383, 1307, 1248, 1219, 1153, 1063, 1027, 1001, 859, 840, 795, 764, 735, 708, 683, 652, 631, 589, 429; Elemental analysis: calcd for (C_57_H_47_N_3_O_2_Si_2_)_6_·(C_7_H_8_): C 79.61; H 5.55; N 4.79. found: C 79.33; H 5.54; N 4.89; mp = 203–204 °C.

for (**2d**)_6_: FT-IR (ATR, cm^–1^): 3302, 3065, 2974, 2933, 1688, 1662, 1607, 1531, 1483, 1391, 1380, 1309, 1234, 1167, 1135, 1080, 1028, 1002, 972, 848, 841, 787, 764, 747, 710, 686, 671, 645, 626, 461, 423; Elemental analysis: calcd for (C_59_H_53_N_3_O_2_B_2_)_6_·(C_7_H_8_)_0.4_: C 76.98; H 5.82; N 4.53. found: C 76.70; H 5.82; N 4.50; mp = 227–231 °C.

### Construction of Porous Network

To a 50 mL round bottom flask, molecular tweezers **2a** (150 mg, 0.181 mmol) was dissolved in toluene (20 mL), and subsequently *n*-hexane (10 mL) was slowly layered on the solution. The flask was placed at room temperature. After 1 h, pale orange crystals of porous network (**2a**)_*n*_ were obtained (99.0 mg, 66%). Under the same procedure, (**2b**)_*n*_ (70% yield, pale yellow crystals) and (**2e**)_*n*_ (80% yield, orange crystals) were prepared from **2b** and **2e**, respectively

for (**2a**)_*n*_: FT-IR (ATR, cm^–1^): 3276, 3071, 3022, 1684, 1674, 1607, 1584, 1533, 1490, 1433, 1345, 1030, 900, 836, 794, 705, 602, 550; Elemental analysis: calcd for C_47_H_29_N_3_O_2_Br_2_·(C_7_H_8_)_0.6_·(H_2_O)_0.5_: C 68.95; H 3.93; N 4.71. found: C 68.85; H 4.03; N 5.01; mp = 193–195 °C.

for (**2b**)_*n*_: FT-IR (ATR, cm^–1^): 3272, 3074, 3023, 1683, 1672, 1606, 1585, 1550, 1535, 1532, 1490, 1442, 1433, 1351, 1263, 1227, 1029, 929, 795, 756, 729, 705, 684, 603, 553, 465; Elemental analysis: calcd for C_47_H_29_N_3_O_2_Cl_2_·(C_7_H_8_)_0.5_·(H_2_O)_0.4_: C 76.59; H 4.30; N 5.31. found: C 76.33; H 4.39; N 5.60; mp = 171–174 °C.

for (**2e**)_*n*_: FT-IR (KBr, cm^–1^): 3371, 3090, 3046, 1686, 1585, 1540, 1520, 1490, 1436, 1402, 1348, 1301, 1216, 1070, 997, 851, 796, 769, 733, 704; Elemental analysis: calcd for C_47_H_29_N_5_O_6_·C_7_H_8_: C 76.13; H 4.38; N 8.22. found: C 75.91; H 4.60; N 8.23; mp = 200–203 °C.

### Construction of Heterologous Cyclic Hexamer

To a 50 mL round bottom flask, molecular tweezers **2c** (43.1 mg, 0.050 mmol) and **2d** (46.1 mg, 0.050 mmol) were dissolved in toluene (10 mL), and subsequently *n*-hexane (20 mL) was slowly layered on the solution. The flask was placed at room temperature. After 12 h, yellow crystals of heterologous cyclic hexamer (**2c**)_*n*_·(**2d**)_6–*n*_ (*n* = 1–6) were obtained (53.0 mg, 59%). FT-IR (KBr, cm^–1^): 3314, 3060, 2977, 2145, 2115, 1688, 1606, 1539, 1489, 1437, 1418, 1383, 1313, 1240, 1139, 1074, 1063, 1028, 1001, 975, 879, 844, 795, 767, 732, 710; Elemental analysis: calcd for (C_57_H_47_N_3_O_2_Si_2_)_3_·(C_59_H_53_N_3_O_2_B_2_)_3_·(C_7_H_8_)_2_·(H_2_O)_3_: C 77.78; H 5.81; N 4.51. found: C 77.57; H 6.00; N 4.27; mp = 220–223 °C.

### Construction of Pseudo Cyclic Dimer

To a 10 mL round bottom flask, molecular tweezers **2c** (30.1 mg, 0.0348 mmol) was dissolved in toluene (1 mL) and acetone (one drop); thereafter, *n*-hexane (3 mL) was slowly layered on the solution. The flask was placed at room temperature. After 12 h, the yellow crystals of pseudocyclic dimer (**2c**)_2_–acetone adducts were obtained (23.0 mg, 76%). FT-IR (KBr, cm^–1^): 3354, 3309, 2957, 2146, 2120, 1690, 1604, 1534, 1490, 1448, 1429, 1383, 1312, 1249, 1153, 1128, 1080, 1064, 1000, 846, 796, 767, 717; Elemental analysis: calcd for (C_57_H_47_N_3_O_2_Si_2_)_2_·(C_3_H_6_O)_2_: C 78.31; H 5.81; N 4.57. found: C 78.04; H 6.10; N 4.48; mp = 197–201 °C.

### Dynamic Interconversion in the Solid State

Crystalline powders of molecular tweezers **2c** were set on a PXRD sample holder and placed in a 300 mL glass bottle with 100 mL of toluene or acetone. After exposure to solvent vapor for an appropriate time, PXRD analysis was performed.

## Data Availability

The data underlying this study are available in the published article and its Supporting Information.

## Supporting Information Available

Synthetic schemes; experimental details and spectroscopic data (NMR, MS, UV–vis, and fluorescence spectra); titration experiment; theoretical calculation; and crystallographic data (SXRD and PXRD) (PDF)

## References

[ref1] Steed J. W.; Atwood J. L. Supramolecular Chemistry; John Wiley & Sons, 2013.

[ref2] Ariga K.; Ito H.; Hill J. P.; Tsukube H. Molecular Recognition: From Solution Science to Nano/materials Technology. Chem. Soc. Rev. 2012, 41, 5800–5835. 10.1039/c2cs35162e. 22773130

[ref3] Morimoto M.; Bierschenk S. M.; Xia K. T.; Bergman R. G.; Raymond K. N.; Toste F. D. Advances in Supramolecular Host-Mediated Reactivity. Nat. Catal. 2020, 3, 969–984. 10.1038/s41929-020-00528-3.

[ref4] Montà-González G.; Sancenón F.; Martínez-Máñez R.; Martí-Centelles V. Purely Covalent Molecular Cages and Containers for Guest Encapsulation. Chem. Rev. 2022, 122, 13636–13708. 10.1021/acs.chemrev.2c00198. 35867555 PMC9413269

[ref5] Cook T. R.; Vajpayee V.; Lee M. H.; Stang P. J.; Chi K.-W. Biomedical and Biochemical Applications of Self-Assembled Metallacycles and Metallacages. Acc. Chem. Res. 2013, 46, 2464–2474. 10.1021/ar400010v. 23786636 PMC3833955

[ref6] Sawada T.; Fujita M. Folding and Assembly of Metal-Linked Peptidic Nanostructures. Chem. 2020, 6, 1861–1876. 10.1016/j.chempr.2020.07.002.

[ref7] Pullen S.; Tessarolo J.; Clever G. H. Increasing Structural and Functional Complexity in Self-Assembled Coordination Cages. Chem. Sci. 2021, 12, 7269–7293. 10.1039/D1SC01226F. 34163819 PMC8171321

[ref8] Lei Y.; Li Z.; Wu G.; Zhang L.; Tong L.; Tong T.; Chen Q.; Wang L.; Ge C.; Wei Y.; Pan Y.; Sue A. C.-H.; Wang L.; Huang F.; Li H. A Trefoil Knot Self-Templated through Imination in Water. Nat. Commun. 2022, 13, 3557 10.1038/s41467-022-31289-1. 35729153 PMC9213439

[ref9] Ono K.; Iwasawa N. Dynamic Behavior of Covalent Organic Cages. Chem.—Eur. J. 2018, 24, 17856–17868. 10.1002/chem.201802253. 29989667

[ref10] Benchimol E.; Nguyen B.-N. T.; Ronson T. K.; Nitschke J. R. Transformation Networks of Metal-Organic Cages Controlled by Chemical Stimuli. Chem. Soc. Rev. 2022, 51, 5101–5135. 10.1039/D0CS00801J. 35661155 PMC9207707

[ref11] Onuchic J. N.; Wolynes P. G. Theory of Protein Folding. Curr. Opin. Struct. Biol. 2004, 14, 70–75. 10.1016/j.sbi.2004.01.009. 15102452

[ref12] Leininger S.; Olenyuk B.; Stang P. J. Self-Assembly of Discrete Cyclic Nanostructures Mediated by Transition Metals. Chem. Rev. 2000, 100, 853–908. 10.1021/cr9601324. 11749254

[ref13] Kobayashi K.; Yamanaka M. Self-Assembled Capsules Based on Tetrafunctionalized Calix[4]resorcinarene Cavitands. Chem. Soc. Rev. 2015, 44, 449–466. 10.1039/C4CS00153B. 24938592

[ref14] Desiraju G. R. Supramolecular Synthons in Crystal Engineering—A New Organic Synthesis. Angew. Chem., Int. Ed. Engl. 1995, 34, 2311–2327. 10.1002/anie.199523111.

[ref15] Moulton B.; Zaworotko M. J. From Molecules to Crystal Engineering: Supramolecular Isomerism and Polymorphism in Network Solids. Chem. Rev. 2001, 101, 1629–1658. 10.1021/cr9900432. 11709994

[ref16] Yamagishi H. Functions and Fundamentals of Porous Molecular Crystals Sustained by Labile Bonds. Chem. Commun. 2022, 58, 11887–11897. 10.1039/D2CC04719E. 36193786

[ref17] Hisaki I.; Xin C.; Takahashi K.; Nakamura T. Designing Hydrogen-Bonded Organic Frameworks (HOFs) with Permanent Porosity. Angew. Chem., Int. Ed. 2019, 58, 11160–11170. 10.1002/anie.201902147. 30891889

[ref18] Song X.; Wang Y.; Wang C.; Wang D.; Zhuang G.; Kirlikovali K. O.; Li P.; Farha O. K. Design Rules of Hydrogen-Bonded Organic Frameworks with High Chemical and Thermal Stabilities. J. Am. Chem. Soc. 2022, 144, 10663–10687. 10.1021/jacs.2c02598. 35675383

[ref19] Gil-Ramírez G.; Benet-Buchholz J.; Escudero-Adan E. C.; Ballester P. Solid-State Self-Assembly of a Calix[4]pyrrole-Resorcinarene Hybrid into a Hexameric Cage. J. Am. Chem. Soc. 2007, 129, 3820–3821. 10.1021/ja070037k. 17348657

[ref20] Yao Y.; Liu J.; Zhang P.; Sun K.; Jin L.; Li S.; Huang F.; Ni Z.; Zhang Z. Solid-State Self-Assembly of Heteroditopic Copillar[5]arenes. Cryst. Growth Des. 2023, 23, 68–76. 10.1021/acs.cgd.2c00711.

[ref21] MacGillivray L. R.; Papaefstathiou G. S.; Friscić T.; Hamilton T. D.; Bucar D.-K.; Chu Q.; Varshney D. B.; Georgiev I. G. Supramolecular Control of Reactivity in the Solid State: From Templates to Ladderanes to Metal-Organic Frameworks. Acc. Chem. Res. 2008, 41, 280–291. 10.1021/ar700145r. 18281948

[ref22] Friščić T.; Mottillo C.; Titi H. M. Mechanochemistry for Synthesis. Angew. Chem., Int. Ed. 2020, 59, 1018–1029. 10.1002/anie.201906755. 31294885

[ref23] Kubota K.; Ito H. Mechanochemical Cross-Coupling Reactions. Trends Chem. 2020, 2, 1066–1081. 10.1016/j.trechm.2020.09.006.

[ref24] Kumar A.; Dutta S.; Kim S.; Kwon T.; Patil S. S.; Kumari N.; Jeevanandham S.; Lee I. S. Solid-State Reaction Synthesis of Nanoscale Materials: Strategies and Applications. Chem. Rev. 2022, 122, 12748–12863. 10.1021/acs.chemrev.1c00637. 35715344

[ref25] Atwood J. L.; Barbour L. J.; Jerga A. Polymorphism of Pure *p*-*tert*-Butylcalix[4]arene: Conclusive Identification of the Phase Obtained by Desolvation. Chem. Commun. 2002, 24, 2952–2953. 10.1039/b209718b. 12536762

[ref26] Ananchenko G. S.; Moudrakovski I. L.; Coleman A. W.; Ripmeester J. A. A Channel-Free Soft-Walled Capsular Calixarene Solid for Gas Adsorption. Angew. Chem., Int. Ed. 2008, 47, 5616–5618. 10.1002/anie.200800071. 18567038

[ref27] Atwood J. L.; Barbour L. J.; Jerga A. Storage of Methane and Freon by Interstitial van der Waals Confinement. Science 2002, 296, 2367–2369. 10.1126/science.1072252. 12004074

[ref28] McKinlay R. M.; Atwood J. L. A Hydrogen-Bonded Hexameric Nanotoroidal Assembly. Angew. Chem., Int. Ed. 2007, 46, 2394–2397. 10.1002/anie.200604453. 17315143

[ref29] Thallapally P. K.; McGrail B. P.; Atwood J. L.; Gaeta C.; Tedesco C.; Neri P. Carbon Dioxide Capture in a Self-Assembled Organic Nanochannels. Chem. Mater. 2007, 19, 3355–3357. 10.1021/cm0709121.

[ref30] Yamasaki Y.; Sekiya R.; Haino T. Hexameric Assembly of 5,17-Di-Substituted Calix[4]arene in the Solid State. CrystEngComm 2017, 19, 6744–6751. 10.1039/C7CE01515A.

[ref31] Stephenson A.; Argent S. P.; Riis-Johannessen T.; Tidmarsh I. S.; Ward M. D. Structures and Dynamic Behavior of Large Polyhedral Coordination Cages: An Unusual Cage-to-Cage Interconversion. J. Am. Chem. Soc. 2011, 133, 858–870. 10.1021/ja107403p. 21175180

[ref32] Zarra S.; Clegg J. K.; Nitschke J. R. Selective Assembly and Disassembly of a Water-Soluble Fe_10_L_15_ Prism. Angew. Chem., Int. Ed. 2013, 52, 4837–4840. 10.1002/anie.201209694. 23441043

[ref33] Markwell-Heys A. W.; Schneider M. L.; Madridejos J. M. L.; Metha G. F.; Bloch W. M. Self-Sorting of Porous Cu_4_L_2_L′_2_ Metal–organic Cages Composed of Isomerisable Ligands. Chem. Commun. 2021, 57, 2915–2918. 10.1039/D0CC08076D. 33616581

[ref34] Inomata Y.; Sawada T.; Fujita M. Metal-Peptide Nonafoil Knots and Decafoil Supercoils. J. Am. Chem. Soc. 2021, 143, 16734–16739. 10.1021/jacs.1c08094. 34601872

[ref35] Orita A.; Jiang L.; Nakano T.; Ma N.; Otera J. Solventless Reaction Dramatically Accelerates Supramolecular Self-Assembly. Chem. Commun. 2002, 13, 1362–1363. 10.1039/b203651g.

[ref36] Garci A.; Castor K. J.; Fakhoury J.; Do J.-L.; Di Trani J.; Chidchob P.; Stein R. S.; Mittermaier A. K.; Friščić T.; Sleiman H. Efficient and Rapid Mechanochemical Assembly of Platinum(II) Squares for Guanine Quadruplex Targeting. J. Am. Chem. Soc. 2017, 139, 16913–16922. 10.1021/jacs.7b09819. 29058892

[ref37] Liu Y.; Liu F.-Z.; Yan K. Mechanochemical Access to a Short-Lived Cyclic Dimer Pd_2_L_2_: An Elusive Kinetic Species En Route to Molecular Triangle Pd_3_L_3_ and Molecular Square Pd_4_L_4_. Angew. Chem., Int. Ed. 2022, 134, e202116980 10.1002/ange.202116980. 35191567

[ref38] Liu Y.; Liu F.-Z.; Li S.; Liu H.; Yan K. Biasing the Formation of Solution-Unstable Intermediates in Coordination Self-Assembly by Mechanochemistry. Chem.—Eur. J. 2023, 29, e202302563 10.1002/chem.202302563. 37670119

[ref39] Sawanaka Y.; Yamashina M.; Ohtsu H.; Toyota S. A Self-Complementary Macrocycle by a Dual Interaction System. Nat. Commun. 2022, 13, 5648 10.1038/s41467-022-33357-y. 36163173 PMC9512892

[ref40] Barrett E. S.; Dale T. J.; Rebek J. Assembly and Exchange of Resorcinarene Capsules Monitored by Fluorescence Resonance Energy Transfer. J. Am. Chem. Soc. 2007, 129, 3818–3819. 10.1021/ja0700956. 17355138

[ref41] Casini A.; Woods B.; Wenzel M. The Promise of Self-Assembled 3D Supramolecular Coordination Complexes for Biomedical Applications. Inorg. Chem. 2017, 56, 14715–14729. 10.1021/acs.inorgchem.7b02599. 29172467

[ref42] Zhukhovitskiy A. V.; Zhong M.; Keeler E. G.; Michaelis V. K.; Sun J. E. P.; Hore M. J. A.; Pochan D. J.; Griffin R. G.; Willard A. P.; Johnson J. A. Highly Branched and Loop-Rich Gels via Formation of Metal-Organic Cages Linked by Polymers. Nat. Chem. 2016, 8, 33–41. 10.1038/nchem.2390. 26673262 PMC5418868

[ref43] Otte M. Reactions in Endohedral Functionalized Cages. Eur. J. Org. Chem. 2023, 26, e202300012 10.1002/ejoc.202300012.

[ref44] Conn M. M.; Rebek J. Self-Assembling Capsules. Chem. Rev. 1997, 97, 1647–1668. 10.1021/cr9603800. 11851461

[ref45] Hiraoka S.; Harano K.; Shiro M.; Shionoya M. A Self-Assembled Organic Capsule Formed from the Union of Six Hexagram-Shaped Amphiphile Molecules. J. Am. Chem. Soc. 2008, 130, 14368–14369. 10.1021/ja804885k. 18839942

[ref46] Tang M.; Liang Y.; Lu X.; Miao X.; Liu Z. Molecular-Strain Engineering of Double-Walled Tetrahedra. Chem 2021, 7, 2160–2174. 10.1016/j.chempr.2021.05.004.

[ref47] Wheeler S. E. Understanding Substituent Effects in Noncovalent Interactions Involving Aromatic Rings. Acc. Chem. Res. 2013, 46, 1029–1038. 10.1021/ar300109n. 22725832

[ref48] Hirayama S.; Kajiwara Y.; Nakayama T.; Hamanoue K.; Teranishi H. Correct Assighment of the Low-Temperature Luminescence from 9-Nitroanthracene. J. Phys. Chem. 1985, 89, 1945–1947. 10.1021/j100256a026.

[ref49] Sahu S.; Parthasarathy V.; Mishra A. K. Phenylethynylanthracene Based Push-Pull Molecular Systems: Tuning the Photophysics through *para*-Substituents on the Phenyl Ring. Phys. Chem. Chem. Phys. 2023, 25, 1957–1969. 10.1039/D2CP05074A. 36541448

[ref50] Lusi M. A Rough Guide to Molecular Solid Solutions: Design, Synthesis and Characterization of Mixed Crystals. CrystEngComm 2018, 20, 7042–7052. 10.1039/C8CE00691A.

[ref51] Hashimoto T.; Oketani R.; Nobuoka M.; Seki S.; Hisaki I. Single Crystalline, Non-Stoichiometric Cocrystals of Hydrogen-Bonded Organic Frameworks. Angew. Chem., Int. Ed. 2023, 135, e202215836 10.1002/ange.202215836. 36347770

[ref52] Vittal J. J. Supramolecular Structural Transformations Involving Coordination Polymers in the Solid State. Coord. Chem. Rev. 2007, 251, 1781–1795. 10.1016/j.ccr.2007.02.002.

[ref53] Chaudhary A.; Mohammad A.; Mobin S. M. Recent Advances in Single-Crystal-to-Single-Crystal Transformation at the Discrete Molecular Level. Cryst. Growth Des. 2017, 17, 2893–2910. 10.1021/acs.cgd.7b00154.

[ref54] Waller P. J.; Gándara F.; Yaghi O. M. Chemistry of Covalent Organic Frameworks. Acc. Chem. Res. 2015, 48, 3053–3063. 10.1021/acs.accounts.5b00369. 26580002

