# Supplementary material for: Solid-State Self-Assembly: Exclusive Formation and Dynamic Interconversion of Discrete Cyclic Assemblies Based on Molecular Tweezers
Source: J Org Chem. 2024 Jun 24;89(13):9488–95. doi: 10.1021/acs.joc.4c00794 (PMC11232003; doi:10.1021/acs.joc.4c00794)
Supplement: Supplementary file 1 — jo4c00794_si_001.pdf [file jo4c00794_si_001.pdf]

Supporting information

Solid-State Self-Assembly – Exclusive Formation and Dynamic Interconversion of Discrete Cyclic Assemblies based on Molecular Tweezers

Koki Okabe, Masahiro Yamashina,* Eiji Tsurumaki, Hidehiro Uekusa, and Shinji Toyota*

Department of Chemistry, School of Science, Tokyo Institute of Technology, 2-12-1 Ookayama, Meguro-ku, Tokyo 152-8551, Japan

*E-mails: yamashina@chem.titech.ac.jp, stoyota@chem.titech.ac.jp

Table of Contents

1. General S3

2. Synthesis S7

2.1. Synthesis of 9-bromo-10-(3-nitrophenyl)anthracene (**3a**) S7

2.2. Synthesis of 9-chloro-10-(3-nitrophenyl)anthracene (**3b**) S7

2.3. Synthesis of 9-(3-nitrophenyl)-10-[(trimethylsilyl)ethynyl]anthracene (**3c**) S8

2.4. Synthesis of 4,4,5,5-tetramethyl-2-(10-(3-nitrophenyl)anthracene-9-yl)-1,3,2-dioxaborolane (**3d**) S9

2.5. Synthesis of 9-nitro-10-(3-nitrophenyl)anthracene (**3e**) S9

2.6. Synthesis of **4a**–**4e** S10

2.7. Association constant for dimerization S13

3. Formation of solid-state self-assemblies S14

3.1. Construction of cyclic hexamer S14

3.2. Construction of porous network S14

3.3. Single crystal X-ray diffraction measurements S15

3.4. Powder X-ray diffraction measurements S18

4. Solid-state physical properties S21

4.1. UV-vis and fluorescence spectra S21

4.2. Thermal analysis S22

4.3. Hirshfeld surface analysis S23

5. Formation of heterologous cyclic hexamers S24

5.1. Construction of heterologous cyclic hexamers S24

5.2. Solid-state physical properties S28

6. Dynamic Interconversion S29

6.1. Construction of pseudo cyclic dimer S29

6.2. Solid-state physical properties of pseudo cyclic dimer S31

6.3. Dynamic interconversion in the solid state S32

7. NMR and MS spectra S36

8. Cartesian Coordinates S58

1. General

Materials and methods

Unless otherwise stated, all reagents and solvents were purchased from commercial sources and used as received. An oil bath was used for all reactions. NMR spectra were recorded using a JEOL 500 MHz JNM-ECX500 spectrometer, a 500 MHz JNM-ECZ500 spectrometer, and a Bruker 500 MHz Avance III HD Smart Probe NMR spectrometer. Chemical shifts for ^{1}H and ^{13}C are reported in ppm on the δ scale; ^{1}H and ^{13}C signals were referenced to the residual solvent peak. Structural assignments were made with additional information from COSY, NOESY, and HMQC experiments. High-resolution electrospray ionization mass spectrometry (HRMS-ESI) were performed on a Bruker micrOTOF II mass Spectrometer. High-resolution FD mass spectra were measured on a JEOL JMS-T100 mass spectrometer using PFTBA (perfluorotri-*n*-butylamine for FD^{+}) or polystyrene. Attenuated Total Reflection Fourier transform infrared (ATR-FTIR) spectra were measured by a JASCO FT/IR 4100. X-ray crystallography was carried out with a Rigaku XtaLAB Synergy R/DW HyPix diffractometer with multi-layer mirror monochromated Cu K_{α} radiation (λ = 1.54184 Å) and processed on a CrysAlisPro 1.171 (Rigaku Oxford Diffraction, 2018) and a diffractometer equipped in a beamline BL-5A at the High Energy Accelerator Research Organization (KEK), Japan with a Pilatus3 S6M detector (synchrotron, λ = 0.7500 Å) and processed on an XDS ver 2022.01.10.[S1] The structure was solved by intrinsic phasing methods using SHELXT-2018[S2] and then refined with SHELXL-2016[S3] using the Olex2.1.5[S4] as graphical user interfaces. All non-hydrogen atoms were refined with anisotropic displacement parameters. All hydrogen atoms were created with ideal geometry and refined using a riding model. Powder X-ray diffraction data were collected for samples on a Rigaku SmartLab instruments in house using transmission geometry mode (Cu X-ray source with D/teX Ultra detector, 2 theta range: 3–40°, scan step: 0.03°). Thermal analysis was carried out with a Shimadzu DTG-60 instrument. Elemental analyses were performed on a Perkin-Elmer 2400 instrument. UV-visible absorption spectroscopy and fluorescence spectroscopy were performed using a JASCO V-730 UV-Vis-NIR and a JASCO FP-8550 spectrofluorometers, respectively. Samples were analyzed at room temperature using quartz cuvettes with optical path lengths of 2.0 mm. Fluorescence lifetime was recorded using a Hamamatsu C7700-ABS-N. Solid-state fluorescence and absolute photoluminescence quantum yield were recorded using a Hamamatsu Quantaurus-QY C11347–01. Melting points were measured using a

Yanako MP-500P micro melting point apparatus without standard correction and data are uncorrected. All DFT calculations were performed by using Gaussian 16 program suite.[S5] Hirshfeld surface analysis was carried out with CrystalExplorer program.[S6,S7]

References

S1. Kabsch, W. Integration, Scaling, Space-Group Assignment and Post-Refinement. *Acta Crystallogr., Sect. D: Biol. Crystallogr.* **2010**, *66*, 133–144.

S2. Sheldrick, G. M. SHELXT – Integrated Space-Group and Crystalstructure Determination. *Acta Crystallogr.* **2015**, *A71*, 3–8.

S3. Sheldrick, G. M. Crystal Structure Refinement with SHELXL. *Acta Crystallogr.* **2015**, *C71*, 3–8.

S4. Dolomanov, O. V.; Bourhis, L. J.; Gildea, R. J.; Howard, J. A. K.; Puschmann, H. OLEX2: A Complete Structure Solution, Refinement and Analysis Program. *J. Appl. Crystallogr.* **2009**, *42*, 339–341.

S5. Gaussian 16, Revision C.01, Frisch, M. J.; Trucks, G. W.; Schlegel, H. B.; Scuseria, G. E.; Robb, M. A.; Cheeseman, J. R.; Scalmani, G.; Barone, V.; Petersson, G. A.; Nakatsuji, H.; Li, X.; Caricato, M.; Marenich, A. V.; Bloino, J.; Janesko, B. G.; Gomperts, R.; Mennucci, B.; Hratchian, H. P.; Ortiz, J. V.; Izmaylov, A. F.; Sonnenberg, J. L.; Williams-Young, D.; Ding, F.; Lipparini, F.; Egidi, F.; Goings, J.; Peng, B.; Petrone, A.; Henderson, T.; Ranasinghe, D.; Zakrzewski, V. G.; Gao, J.; Rega, N.; Zheng, G.; Liang, W.; Hada, M.; Ehara, M.; Toyota, K.; Fukuda, R.; Hasegawa, J.; Ishida, M.; Nakajima, T.; Honda, Y.; Kitao, O.; Nakai, H.; Vreven, T.; Throssell, K.; Montgomery Jr., J. A.; Peralta, J. E.; Ogliaro, F.; Bearpark, M. J.; Heyd, J. J.; Brothers, E. N.; Kudin, K. N.; Staroverov, V. N.; Keith, T. A.; Kobayashi, R.; Normand, J.; Raghavachari, K.; Rendell, A. P.; Burant, J. C.; Iyengar, S. S.; Tomasi, J.; Cossi, M.; Millam, J. M.; Klene, M.; Adamo, C.; Cammi, R.; Ochterski, J. W.; Martin, R. L.; Morokuma, K.; Farkas, O.; Foresman, J. B.; Fox, D. J. Gaussian, Inc., Wallingford CT, USA, **2016**.

S6. Spackman, P. R.; Turner, M. J.; McKinnon, J. J.; Wolff, S. K.; Grimwood, D. J.; Jayatilaka, D.; Spackman, M. A. CrystalExplorer: A Program for Hirshfeld Surface Analysis, Visualization and Quantitative Analysis of Molecular Crystals. *J. Appl. Crystallogr.* **2021**, *54*, 1006–1011.

S7. Spackman, M. A.; Jayatilaka, D. Hirshfeld Surface Analysis. *CrystEngComm* **2009**, *11*, 19–32.

S8. Sawanaka, Y.; Yamashina, M.; Ohtsu, H.; Toyota, S. A Self-Complementary Macrocycle by a Dual Interaction System. *Nat. Commun.* **2022**, *13*, 5648.

S9. Du, J.; Kodikara, M. S.; Moxey, G. J.; Morshedi, M.; Barlow, A.; Quintana, C.; Wang, G.; Stranger, R.; Zhang, C.; Cifuentes, M. P.; Humphrey, M. G. Quadratic and Cubic Hyperpolarizabilities of Nitro-Phenyl/-Naphthalenyl/-Anthracenyl Alkynyl Complexes. *Dalton Trans.* **2018**, *47*, 4560–4571.

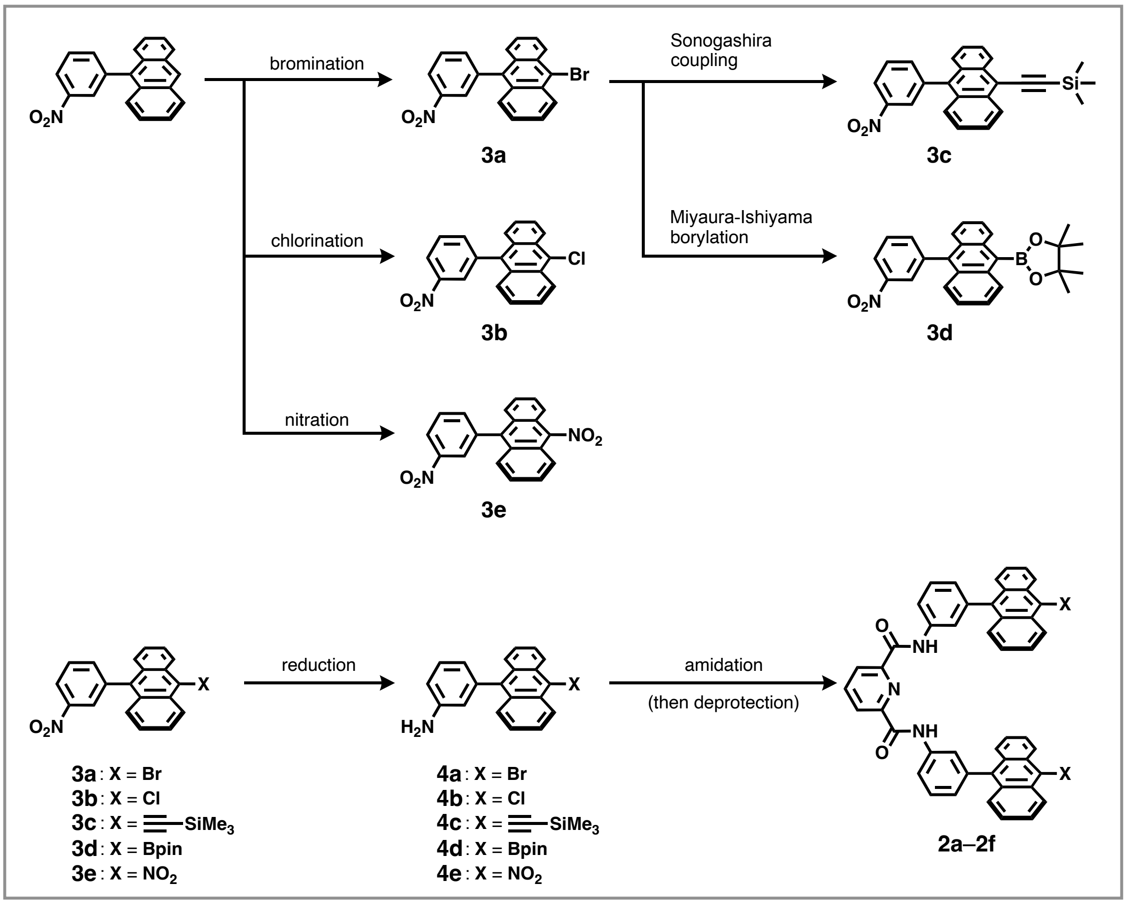

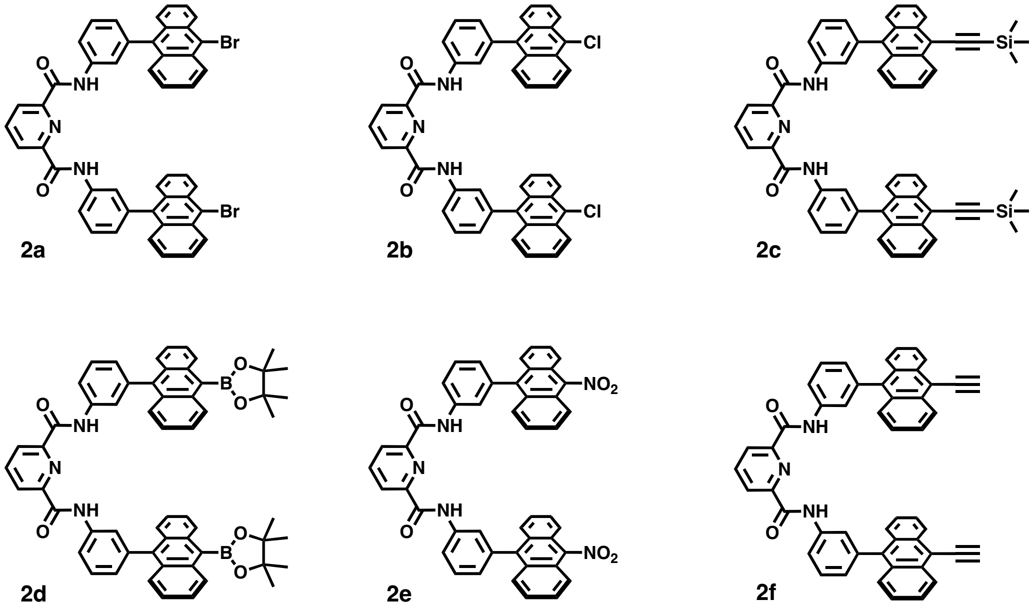

Figure S1. Synthetic scheme of molecular tweezers **2a**–**2f** and their chemical structures.

2. Synthesis

2.1. Synthesis of 9-bromo-10-(3-nitrophenyl)anthracene (3a) KO-1-083

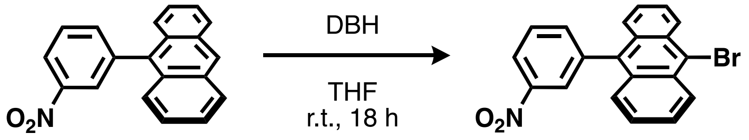

98% **3a**

9-(3-Nitrophenyl)anthracene[S8] (1.00 g, 3.34 mmol) and THF (60 mL) were added to a 200 mL round bottom flask, and 1,3-dibromo-5,5-dimethylhydantoin (DBH; 0.573 g, 2.00 mmol) was subsequently added to the flask at 0 °C. After stirring at room temperature for 18 h, the resultant mixture was concentrated under reduced pressure. The crude product was washed with methanol to afford **3a** as a yellow solid (1.24 g, 98%).

^{1}H NMR (500 MHz, $CDCl_3$, 298 K): δ 8.65 (dd, J = 9.1, 1.1 Hz, 2H), 8.44 (ddd, J = 7.9, 2.3, 1.1 Hz, 1H), 8.31 (dd, J = 2.3, 1.7 Hz, 1H), 7.80 (dd, J = 7.9, 7.4 Hz, 1H), 7.76 (ddd, J = 7.4, 1.7, 1.1 Hz, 1H), 7.62 (ddd, J = 9.1, 6.2, 1.1 Hz, 2H), 7.50 (d, J = 9.1 Hz, 2H), 7.42 (ddd, J = 9.1, 6.2, 1.1 Hz, 2H); ^{13}C{^{1}H} NMR (125 MHz, $CDCl_3$, 298 K): δ 148.7 (C_q), 140.5 (C_q), 137.5 (CH), 134.5 (C_q), 130.9 (C_q), 130.3 (C_q), 129.8 (CH), 128.4 (CH), 127.3 (CH), 126.50 (CH), 126.55 (CH), 126.2 (CH), 124.3 (C_q), 123.1 (CH); HRMS (FD) *m/z*: $[M]^+$ calcd for $C_{20}H_{12}{}^{79}BrNO_2$, 377.0051; found 377.0051; m.p. = 182–184 °C.

2.2. Synthesis of 9-chloro-10-(3-nitrophenyl)anthracene (3b) KO-2-013

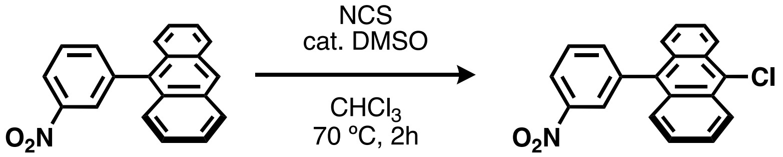

62% **3b**

9-(3-Nitrophenyl)anthracene (80.3 mg, 0.268 mmol), *N*-chlorosuccinimide (NCS; 71.2 mg, 0.533 mmol), and $CHCl_3$ (5 mL) were added to a 10 mL vial tube. DMSO (2.75 mg, 0.0352 mmol) was subsequently added to the solution. After stirring at 70 °C for 2 h, the resultant mixture was concentrated under reduced pressure. The crude product was washed with methanol to afford **3b** as a yellow solid (55.5 mg, 62%).

^{1}H NMR (500 MHz, $CDCl_3$, 298 K): δ 8.63 (d, J = 9.1 Hz, 2H), 8.44 (dd, J = 7.4, 1.7 Hz, 1H), 8.31 (dd, J = 2.3, 1.7 Hz, 1H), 7.80 (t, J = 7.4 Hz, 1H), 7.76 (dd, J = 7.4, 1.7 Hz, 1H), 7.63 (ddd, J = 9.1, 6.8, 1.1 Hz, 2H), 7.52 (d, J = 9.1 Hz, 2H), 7.44 (ddd, J = 9.1, 6.8, 1.1 Hz, 2H); ^{13}C{^{1}H} NMR (125 MHz, $CDCl_3$, 298 K): δ 149.2 (C_q), 140.8 (C_q), 138.1 (CH), 134.0 (C_q), 131.1 (C_q), 130.6 (C_q), 130.2 (CH), 129.1 (C_q), 127.4 (CH), 127.0 (CH), 126.9 (CH), 126.8 (CH), 125.8 (CH), 123.5 (CH); HRMS (FD) *m/z*: $[M]^+$ calcd for $C_{20}H_{12}{}^{35}ClNO_2$, 333.0557; found 333.0548; m.p. = 189–193 °C.

2.3. Synthesis of 9-(3-nitrophenyl)-10-[(trimethylsilyl)ethynyl]anthracene (3c)

KO-1-064

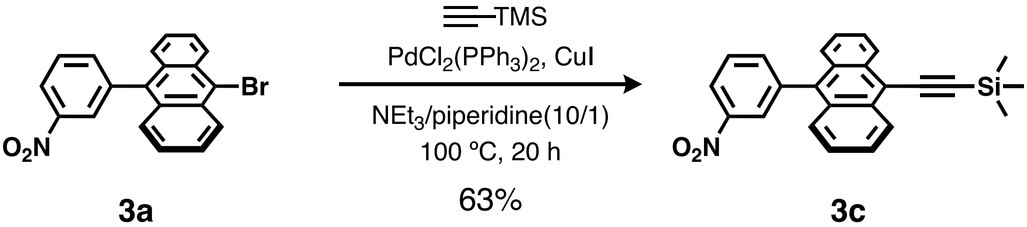

All solvents were degassed by N_2 gas bubbling before use. **3a** (208 mg, 0.550 mmol), $PdCl_2(PPh_3)_2$ (19.3 mg, 0.0275 mmol), CuI (11.6 mg, 0.0609 mmol), triethylamine (4.0 mL) and piperidine (0.40 mL) were added to a 10 mL vial tube under N_2 atmosphere. (Trimethylsilyl)acetylene (108 mg, 1.10 mmol) was subsequently added to the solution. After stirring at 100 °C for 20 h, the resultant mixture was concentrated under reduced pressure. The crude mixture was purified by column chromatography on silica gel (CH_2Cl_2/*n*-hexane = 1/2) to afford **3c** as an orange solid (137 mg, 63%).

^{1}H NMR (500 MHz, $CDCl_3$, 298 K): δ 8.68 (d, J = 9.1 Hz, 2H), 8.43 (dd, J = 8.0, 1.7 Hz, 1H), 8.31 (dd, J =2.3, 1.7 Hz, 1H), 7.79 (dd, J = 7.9, 7.4 Hz, 1H), 7.76 (dd, J = 7.4, 1.7 Hz, 1H), 7.61 (ddd, J = 9.1, 6.8, 1.1 Hz, 2H), 7.51 (d, J = 9.1 Hz, 2H), 7.42 (ddd, J = 9.1, 6.8, 1.1 Hz, 2H), 0.46 (s, 9H); ^{13}C{^{1}H} NMR (125 MHz, $CDCl_3$, 298 K): δ 149.0 (C_q), 141.0 (C_q), 138.0 (CH), 135.5 (C_q), 133.0 (C_q), 130.13 (CH), 127.8 (CH), 127.2 (CH), 127.0 (CH), 126.9 (CH), 126.7 (CH), 123.5 (CH), 119.2 (C_q), 108.0 (C_q), 101.9 (C_q), 0.8 (CH_3). One aromatic C_q signal is overlapped; HRMS (FD) *m/z*: $[M]^+$ calcd for $C_{25}H_{21}NO_2Si$, 395.1342; found 395.1333; m.p. = 168–171 °C.

2.4. Synthesis of 4,4,5,5-tetramethyl-2-(10-(3-nitrophenyl)anthracene-9-yl)-1,3,2-dioxaborolane (3d) KO-2-043

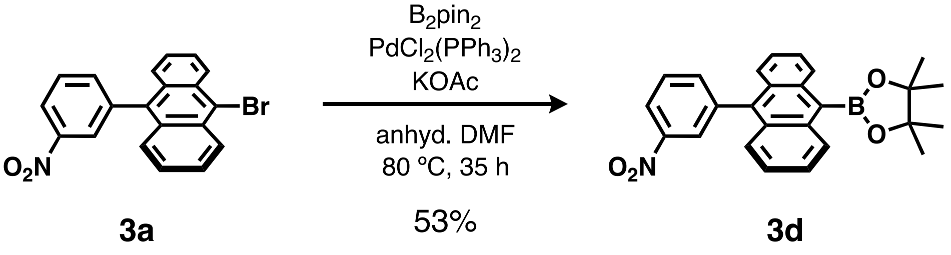

3a (0.833 g, 2.20 mmol), bis(pinacolato)diboron (1.12 g, 4.42 mmol), KOAc (1.42 g, 14.4 mmol), $PdCl_2(PPh_3)_2$ (0.148 g, 0.211 mmol), and anhydrous DMF (30 mL) were added to a 100 mL two-necked flask under N_2. After stirring at 80 °C for 35 h, the resultant mixture was poured into water (100 mL). The precipitate was filtered, and the obtained solid was dried under reduced pressure at 70 °C. The crude product was purified by column chromatography on silica gel (CH_2Cl_2/*n*-hexane = 1/2) to afford **3d** as a yellow solid (500 mg, 53%).

^{1}H NMR (500 MHz, $CDCl_3$, 298 K): δ 8.46 (d, J = 9.1 Hz, 2H), 8.41 (dt, J = 7.9, 1.7 Hz, 1H), 8.29 (t, J = 1.7 Hz, 1H), 7.77 (t, J = 7.4 Hz, 1H), 7.73 (dt, J = 7.4, 1.7 Hz, 1H), 7.51 (ddd, J = 9.1, 6.8, 1.1 Hz, 2H), 7.49 (d, J = 9.1 Hz, 2H), 7.37 (ddd, J = 9.1, 6.8, 1.1 Hz, 2H), 1.62 (s, 12H); ^{13}C{^{1}H} NMR (125 MHz, $CDCl_3$, 298 K): δ 149.0 (C_q), 141.6 (C_q), 138.1 (CH), 136.6 (C_q), 135.8 (C_q), 130.1 (CH), 130.0 (C_q), 129.28 (CH), 126.9 (CH), 126.6 (CH), 126.3 (CH), 126.2 (CH), 123.2 (CH), 85.3 (C_q), 25.8 (CH_3). One aromatic C_q signal is significantly broadened; HRMS (FD) *m/z*: $[M]^+$ calcd for $C_{26}H_{24}{}^{11}BNO_4$, 425.1798; found 425.1819; m.p. = 170–175 °C.

2.5. Synthesis of 9-nitro-10-(3-nitrophenyl)anthracene (3e) KO-2-149

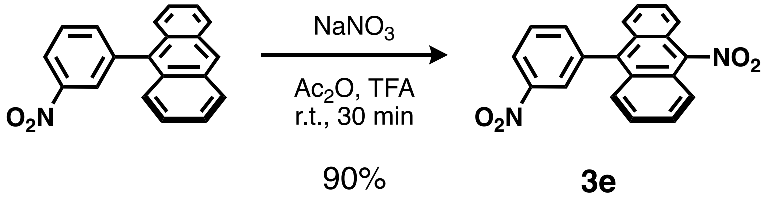

Nitration of anthracene was carried out based on the literature.[S9] 9-(3-Nitrophenyl)anthracene (1.02 g, 3.41 mmol), Ac_2O (50 mL) and TFA (30 mL) were added

to a 200 mL round bottom flask. Subsequently, $NaNO_3$ (0.312 g, 3.67 mmol) was slowly added to the flask. After stirring at room temperature for 30 min, the resultant mixture was poured into H_2O/H_2SO_4 (400 mL:100/1). The precipitate was filtered and washed with water. The crude mixture was dissolved in EtOAc and washed with 10% NaOH aq. The combined organic extract was dried over Na_2SO_4, filtrated, and concentrated under reduced pressure. The obtained solid was purified by short column chromatography on silica gel (CH_2Cl_2) to afford **3e** as a yellow solid (1.06 g, 90%).

^{1}H NMR (500 MHz, $CDCl_3$, 298 K): δ 8.48 (d, J = 8.5 Hz, 1H), 8.32 (s, 1H), 7.98 (d, J = 9.1 Hz, 2H) , 7.84 (t, J = 7.4 Hz, 1H) , 7.76 (d, J = 7.4 Hz, 1H) , 7.68 (t, J = 7.4 Hz, 2H) , 7.57 (d, J = 9.1 Hz, 2H), 7.49 (t, J = 9.1 Hz, 2H); ^{13}C{^{1}H} NMR (125 MHz, $CDCl_3$, 298 K): δ 149.1 (C_q), 145.8 (C_q), 139.8 (C_q), 138.2 (C_q), 137.6 (CH), 130.5 (CH), 130.1 (C_q), 129.3 (CH), 127.5 (CH), 126.8 (CH), 126.4 (CH), 124.0 (CH), 122.6 (C_q), 122.2 (CH); HRMS (FD) *m/z*: $[M]^+$ calcd for $C_{20}H_{12}N_2O_4$, 344.0797; found 344.0781; m.p. = 71–75 °C.

2.6. Synthesis of 4a–4e KO-2-011

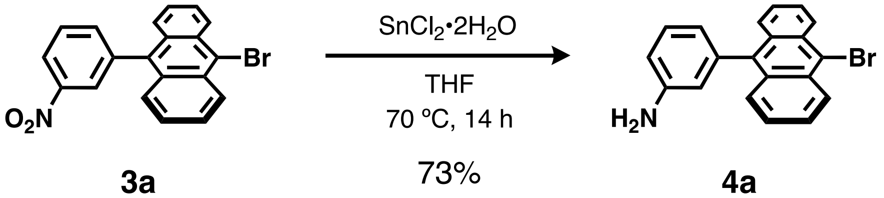

3a 4a

3a (104 mg, 0.264 mmol), $SnCl_2$•$2H_2O$ (108 mg, 0.792 mmol), and THF (3.0 mL) were added to a 10 mL vial tube. After stirring at 70 °C for 14 h, the resultant mixture was concentrated under reduced pressure. The crude mixture was dissolved in EtOAc and washed with 10% NaOH aq. The combined organic extract was dried over Na_2SO_4, filtrated, and concentrated under reduced pressure. The obtained solid was purified by column chromatography on silica gel (CH_2Cl_2/*n*-hexane = 1/1) to afford **4a** as a pale-yellow solid (69.6 mg, 73%). Under the same procedure, **4b** (138 mg, 77% yield, a pale-yellow solid), **4c** (70.5 mg, 77% yield, a yellow solid), **4d** (330 mg, 74% yield, a pale-yellow solid), and **4e** (32.0 mg, 68% yield, a yellow solid) were synthesized from **3b** (200 mg, 0.599 mmol), **3c** (91.5 mg, 0.231 mmol), **3d** (480 mg, 1.13 mmol), and **3e** (52.0 mg, 0.151 mmol), respectively.

for **4a**

^{1}H NMR (500 MHz, $CDCl_3$, 298 K): δ 8.60 (d, J = 9.1 Hz, 2H), 7.76 (d, J = 9.1 Hz, 2H), 7.58 (ddd, J = 9.1, 6.8, 1.1 Hz, 2H), 7.38 (ddd, J = 9.1, 6.8, 1.1 Hz, 2H), 7.36 (t, J = 7.4 Hz, 1H), 6.86 (dd, J = 7.4, 1.7 Hz, 1H), 6.80 (d, J = 7.4 Hz, 1H), 6.73 (t, J = 1.7 Hz, 1H), 3.78 (br, 2H); $^{13}C\{^{1}H\}$ NMR (125 MHz, $CDCl_3$, 298 K): δ 146.9 (C_q), 140.0 (C_q), 138.6 (C_q), 131.4 (C_q), 130.7 (C_q), 129.9 (CH), 128.3 (CH), 128.2 (CH), 127.5 (CH), 126.0 (CH), 123.0 (C_q), 122.2 (CH), 118.3 (CH), 115.0 (CH); HRMS (FD) *m/z*: $[M]^+$ calcd for $C_{20}H_{14}{}^{79}BrN$, 347.0310; found 347.0316; m.p. = 166–169 °C.

for **4b** KO-1-091

^{1}H NMR (500 MHz, $CDCl_3$, 298 K): δ 8.57 (d, J = 9.1 Hz, 2H), 7.77 (d, J = 9.1 Hz, 2H), 7.58 (ddd, J = 9.1, 6.2, 1.1 Hz, 2H), 7.39 (ddd, J = 9.1, 6.2, 1.1 Hz, 2H), 7.36 (t, J = 7.4 Hz, 1H), 6.86 (dd, J = 7.4, 2.3 Hz, 1H), 6.80 (d, J = 7.4 Hz, 1H), 6.73 (s, 1H), 3.78 (br, 2H); $^{13}C\{^{1}H\}$ NMR (125 MHz, $CDCl_3$, 298 K): δ 146.9 (C_q), 140.0 (C_q), 137.7 (C_q), 131.2 (C_q), 129.9 (CH), 129.1 (C_q), 128.9 (C_q), 128.1 (CH), 127.1 (CH), 126.0 (CH), 125.3 (CH), 122.3 (CH), 118.5 (CH), 115.0 (CH); HRMS (FD) *m/z*: $[M]^+$ calcd for $C_{20}H_{14}{}^{35}ClN$, 303.0815; found 303.0814; m.p. = 151–154 °C.

for **4c** KO-1-097

^{1}H NMR (500 MHz, $CDCl_3$, 298 K): δ 8.62 (d, J = 9.1 Hz, 2H), 7.75 (d, J = 9.1 Hz, 2H), 7.56 (ddd, J = 9.1, 6.2, 1.1 Hz, 2H), 7.37 (ddd, J = 9.1, 6.2, 1.1 Hz, 2H), 7.35 (t, J = 7.4 Hz, 1H), 6.85 (ddd, J = 7.4, 2.3, 1.1 Hz, 1H), 6.80 (d, J = 7.4 Hz, 1H), 6.73 (dd, J = 2.3, 1.1, 1H), 3.78 (br, 2H), 0.44 (s, 9H); $^{13}C\{^{1}H\}$ NMR (125 MHz, $CDCl_3$, 298 K): δ 146.9 (C_q), 140.2 (C_q), 139.5 (C_q), 133.1 (C_q), 130.2 (C_q), 129.8 (CH), 128.1 (CH), 127.4 (CH), 127.0 (CH), 126.0 (CH), 122.2 (CH), 118.4 (CH), 117.6 (C_q), 114.9 (CH), 106.9 (C_q), 102.4 (C_q), 0.9 (CH_3); HRMS (FD) *m/z*: $[M]^+$ calcd for $C_{25}H_{23}NSi$, 365.1600; found 365.1595; m.p. = 141–145 °C.

for **4d** KO-2-055

^{1}H NMR (500 MHz, $CDCl_3$, 298 K): δ 8.43 (d, J = 9.1 Hz, 2H), 7.74 (d, J = 9.1 Hz, 2H), 7.47 (ddd, J = 9.1, 6.2, 1.1 Hz, 2H), 7.34 (t, J = 7.4 Hz, 1H), 7.32 (ddd, J = 9.1, 6.2, 1.1 Hz, 2H), 6.84 (dd, J = 7.4, 2.3 Hz, 1H), 6.79 (d, J = 7.4 Hz, 1H), 6.72 (dd, J = 2.3, 1.7 Hz, 1H), 3.76 (br, 2H), 1.59 (s, 12H); $^{13}C\{^{1}H\}$ NMR (125 MHz, $CDCl_3$, 298 K): δ 146.8

(C_q), 140.8 (C_q), 140.4 (C_q), 135.9 (C_q), 130.1 (C_q), 129.8 (CH), 128.8 (CH), 128.2 (CH), 126.0 (CH), 125.3 (CH), 122.1 (CH), 118.4 (CH), 114.7 (CH), 85.0 (C_q), 25.8 (CH_3). One aromatic C_q signal is significantly broadened; HRMS (FD) *m/z*: $[M]^+$ calcd for $C_{26}H_{26}{}^{11}BNO_2$, 395.2057; found 395.2054; m.p. = 231–235 °C.

for **4e** KO-2-127

^1H NMR (500 MHz, $CDCl_3$, 298 K): δ 7.94 (d, J = 9.1 Hz, 2H), 7.80 (d, J = 9.1 Hz, 2H), 7.62 (t, J = 9.1 Hz, 2H), 7.42 (t, J = 9.1 Hz, 2H), 7.37 (t, J = 7.9 Hz, 1H), 6.89 (d, J = 7.9 Hz, 1H), 6.78 (d, J = 7.9 Hz, 1H), 6.70 (s, 1H), 3.83 (br, 2H); $^{13}C\{^1H\}$ NMR (125 MHz, $CDCl_3$, 298 K): δ 146.7 (C_q), 144.5 (C_q), 141.9 (C_q), 138.6 (C_q), 129.7 (CH), 128.7 (CH), 127.7 (CH), 126.1 (CH), 122.4 (C_q), 121.39 (CH), 121.35 (CH), 117.5 (CH), 115.0 (CH). One aromatic C_q signal is overlapped; HRMS (FD) *m/z*: $[M]^+$ calcd for $C_{20}H_{14}N_2O_2$, 314.1055; found 314.1056; m.p. = 229–233 °C.

2.7. Association constant for dimerization

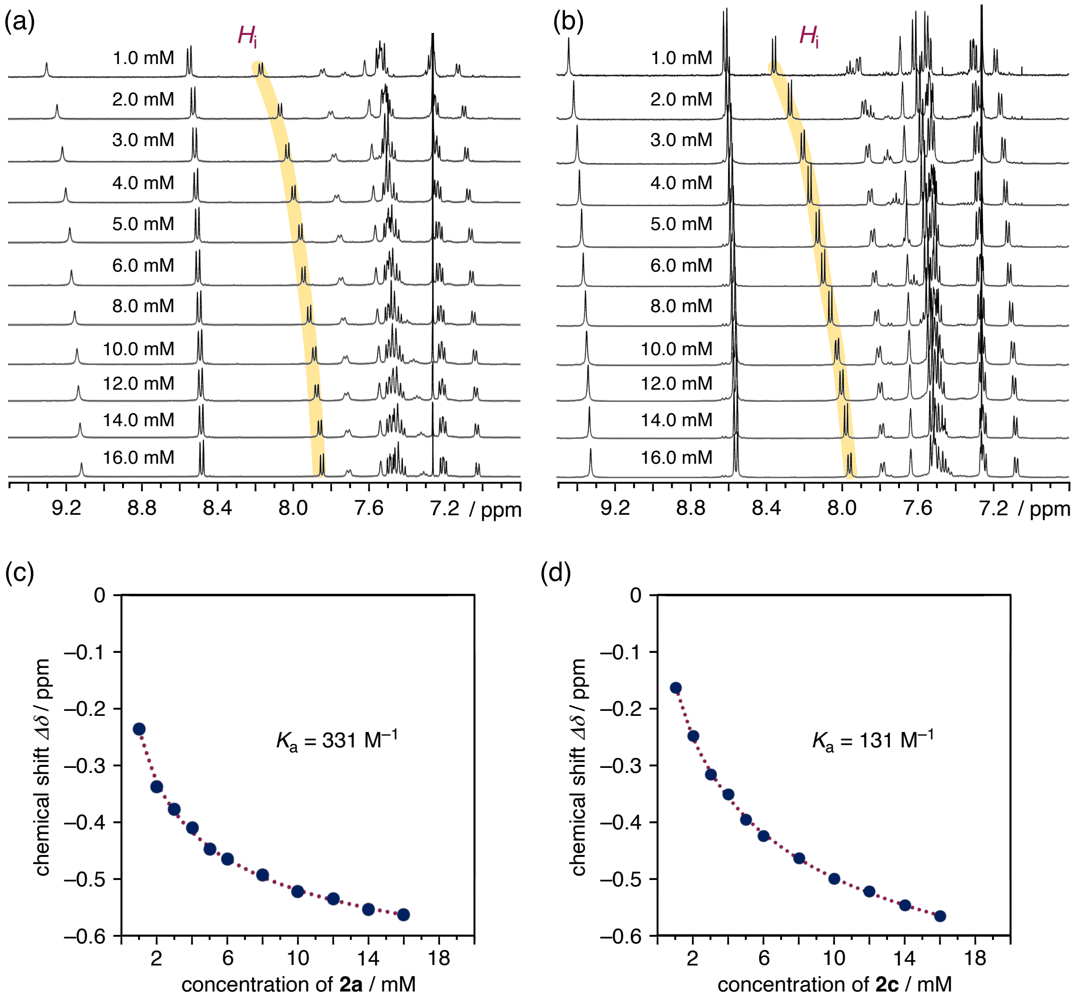

Figure S2. (a) Concentration-dependent ^{1}H NMR spectra (500 MHz, 298 K, $CDCl_3$) of (a) **2a** and (b) **2c**. Titration curves of (c) **2a** and (d) **2c** using H_i with their dimerization constants K_a. The association constant (K_a) for the monomer-dimer exchange model was calculated by curve fitting and a nonlinear least-squares method using the SOLVER program.[S8]

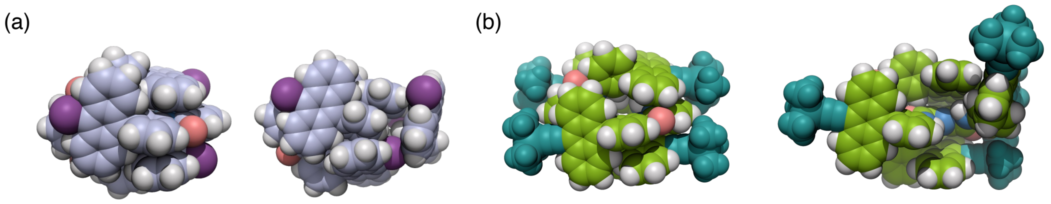

mode	head-to-head	head-to-tail	head-to-head	head-to-tail
ΔE (kJ mol^{-1})	0	+ 2.5	–	–
E (kJ mol^{-1})	−38158528.39445	−38158525.868719		

Figure S3. The optimized structures of possible self-complementary dimers (a) (**2a**)$_2$ calculated by CONFLEX and then DFT method at the B3LYP-D3/6-31G (d,p) level with PCM ($CHCl_3$) and (b) (**2c**)$_2$ by semi-empirical method at PM6 level.

3. Formation of solid-state self-assemblies

3.1. Construction of cyclic hexamer KO-2-175

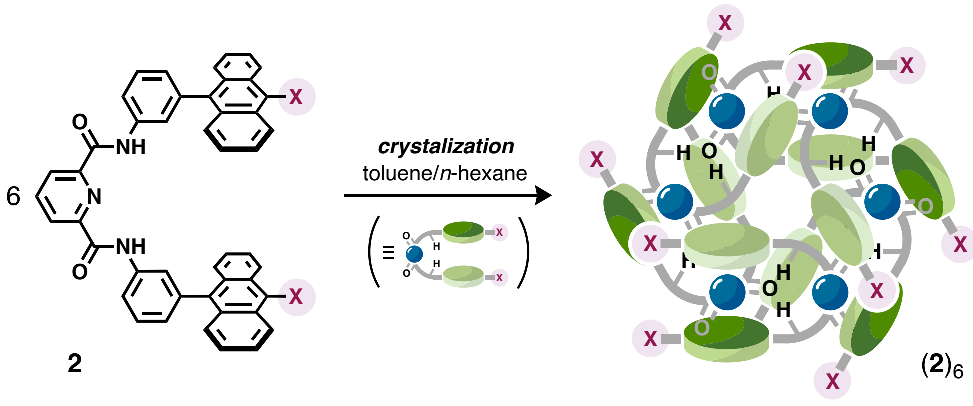

See experimental section in the manuscript for experimental procedure.

3.2. Construction of porous network KO-2-049

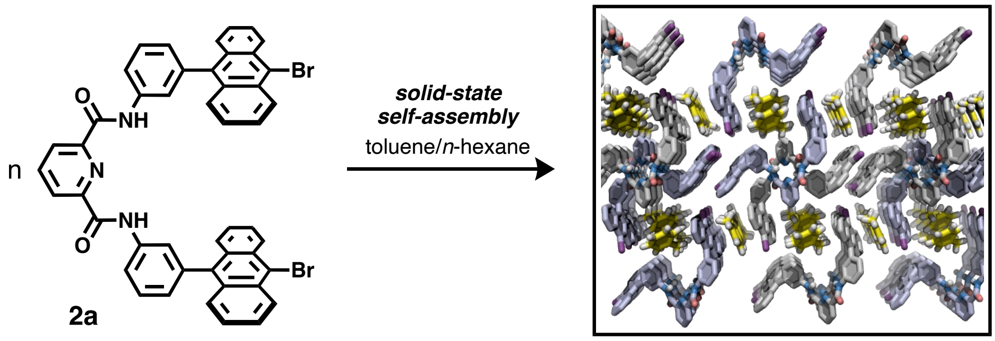

porous network $(\mathbf{2a})_n$

See experimental section in the manuscript for experimental procedure.

3.3. Single crystal X-ray diffraction measurements

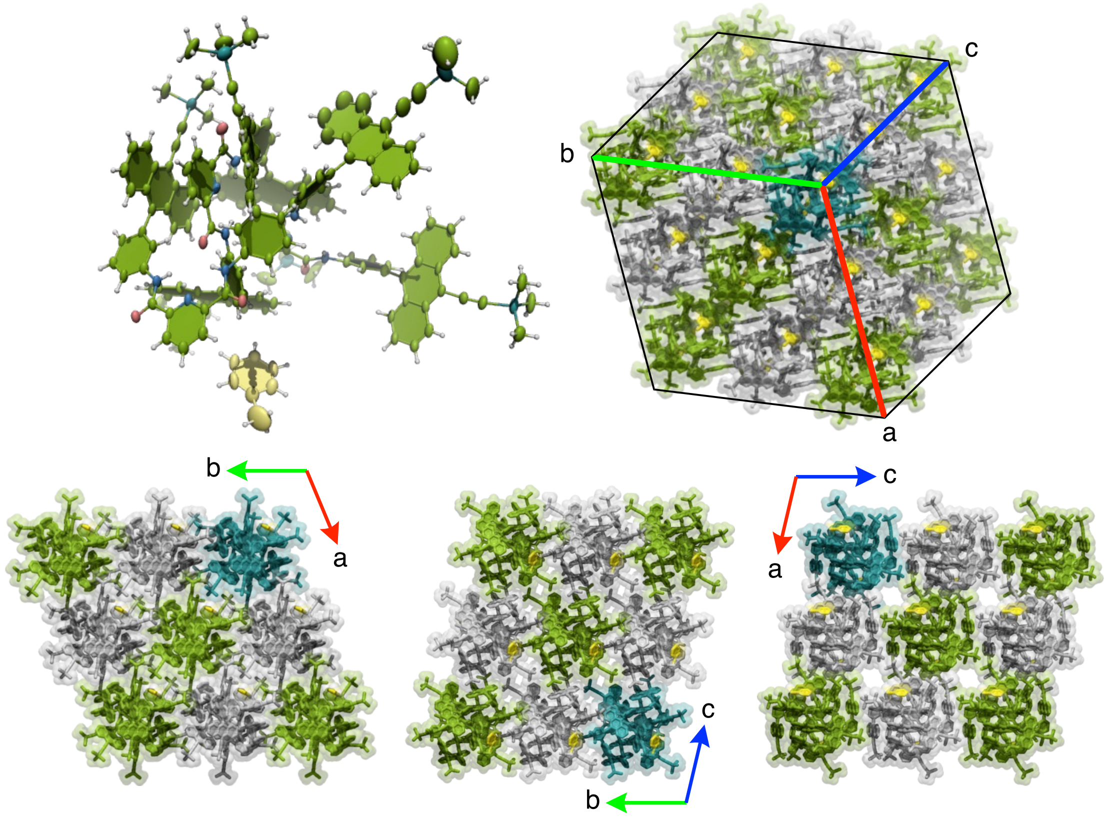

Figure S4. The ORTEP drawing structures of (**2c**)$_6$ and its packing structure. The thermal ellipsoids are drawn at 30% possibility. Green and gray: carbon ((**2c**)$_6$), yellow: carbon (toluene), blue: nitrogen, pink: oxygen, and dark green: silicon.

Note:

There are two alerts A in the crystalline data of (**2c**)$_6$ as below;

PLAT029_ALERT_3_A _diffrn_measured_fraction_theta_full value Low.

Because the measurement could perform only one axis rotation scan (omega scan with chi = 0) using the goniometer, we couldn't collect high completeness data for triclinic space group.

PLAT026_ALERT_3_A Ratio Observed / Unique Reflections (too) Low.

This is partly due to the severe disorder of the crystalline solvents, anthracene arms, and substituents of tweezers. In addition, as we discussed in the main text, the single crystal of (**2c**)$_6$ was fragile in the oil owing to volatile solvents probably because of rapid exchanging crystalline solvents, leading to the loss of their single crystalline nature. Therefore, we applied the solvent mask implemented in Olex2.[S4]

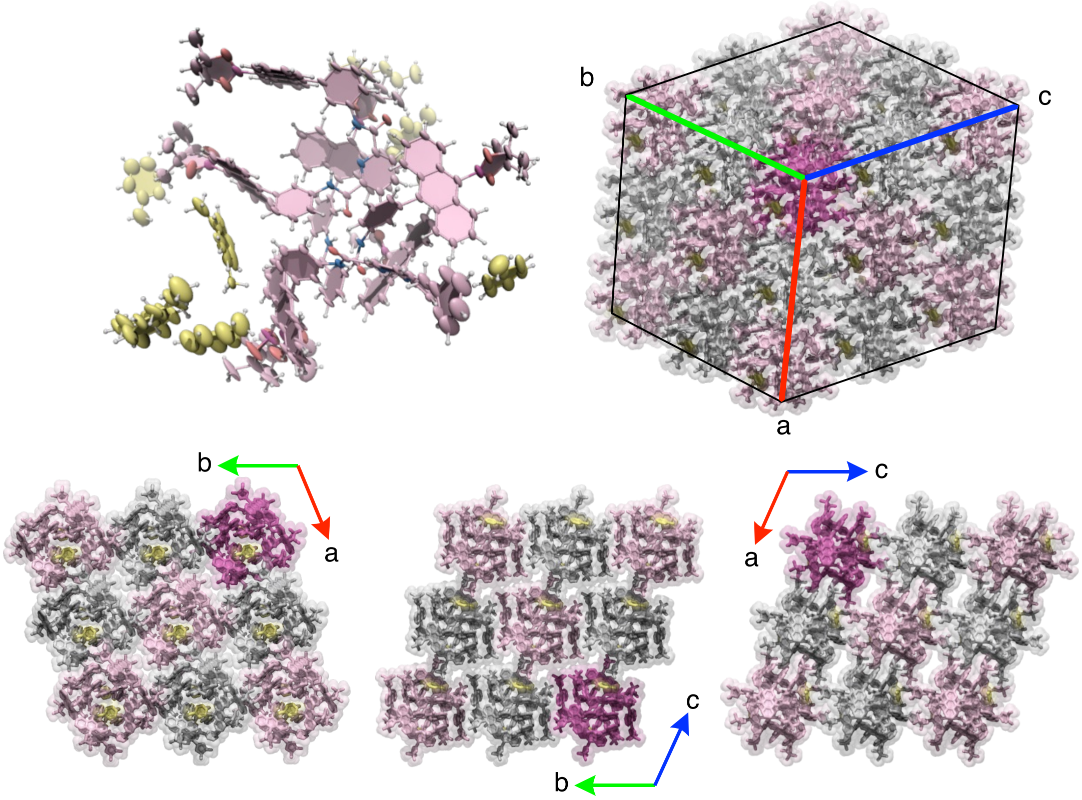

Figure S5. The ORTEP drawing structures of (**2d**)$_6$ and its packing structure. The thermal ellipsoids are drawn at 30% possibility. Light pink and gray: carbon ((**2d**)$_6$), and yellow (toluene and *n*-hexane), blue: nitrogen, pink: oxygen, and magenta: boron. In the packing structures, some of the solvent molecules are omitted for clarity.

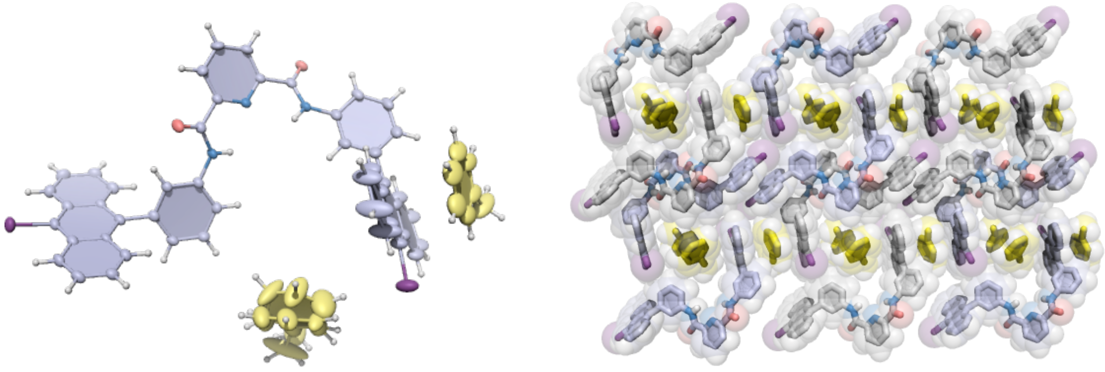

Figure S6. The ORTEP drawing structures of (**2a**)$_n$ and its packing structure. The thermal ellipsoids are drawn at 50% possibility. Light purple and yellow: carbon, blue: nitrogen, pink: oxygen, and purple: bromine.

Table S1. Crystal data and structure refinement.

Compounds	**(2c)**$_6$	**(2d)**$_6$	**(2a)**$_n$
Identification code	TMS6	KO230522	KO-Br-tolhex
CCDC number	2326652	2326654	2326656
Empirical formula	$C_{178}H_{149}N_9O_6Si_6$	$C_{231.31}H_{231.23}B_6N_9O_{18}$	$C_{57.5}H_{41}Br_2N_3O_2$
Formula weight	2678.59	3489.99	965.75
Temperature (K)	93	123.15	123
Wavelength (Å)	0.750	1.54184	1.54184
Crystal system	triclinic	triclinic	triclinic
Space group	P-1	P-1	P-1
Unit cell dimensions			
a (Å)	21.033(5)	20.9021(3)	8.8888(2)
b (Å)	21.187(8)	22.8015(3)	15.2557(3)
c (Å)	22.828(9)	24.0585(4)	17.4871(3)
α (°)	97.904(7)	105.8450(10)	70.288(2)
β (°)	99.65(3)	107.7890(10)	77.878(2)
γ (°)	109.928(10)	98.1980(10)	76.574(2)
Volume (Å^3)	9218(6)	10178.5(3)	2148.80(8)
Z	2	2	2
Density (calculated)	0.965	1.139	1.493
Absorption coefficient	0.108	0.555	2.785
F(000)	2824.0	3712.0	986.0
Crystal size	0.1 × 0.1 × 0.1	0.46 × 0.321 × 0.214	0.483 × 0.09 × 0.068
2θ range for data collection	1.954 to 71.138	4.582 to 134.154	5.424 to 149.002
Index ranges	$-31 \le h \le 31$	$-24 \le h \le 24$	$-10 \le h \le 11$
	$-29 \le k \le 31$	$-27 \le k \le 27$	$-18 \le k \le 19$
	$-26 \le l \le 26$	$-28 \le l \le 28$	$-20 \le l \le 21$
Reflections collected	98710	121601	33394
Independent reflections	55847 [R_{int} = 0.2452]	36150 [Rint = 0.0306]	8756 [R_{int} = 0.0412]
Completeness to θ	0.775	0.995	0.997
Absorption correction	multi-scan	multi-scan	multi-scan
Refinement method		Full-matrix least-squares on F^2	
Data / restraints / parameters	55847/128/1809	36150/2456/2824	8756/174/653
Goodness-of-fit on F^2	0.840	1.533	1.048
Final *R* indices [I>2sigma(I)]	R_1 = 0.1119	R_1 = 0.1197	R_1 = 0.0553
	wR_2 = 0.2419	wR_2 = 0.3507	wR_2 = 0.1443
R indices (all data)	R_1 = 0.3555	R_1 = 0.1431	R_1 = 0.0663
	wR_2 = 0.3726	wR_2 = 0.3759	wR_2 = 0.1512
Largest diff. peak and hole	0.55 and -0.37 e Å^{-3}	0.88 and -0.70 e Å^{-3}	0.89 and -1.46 e Å^{-3}

3.4. Powder X-ray diffractions measurements

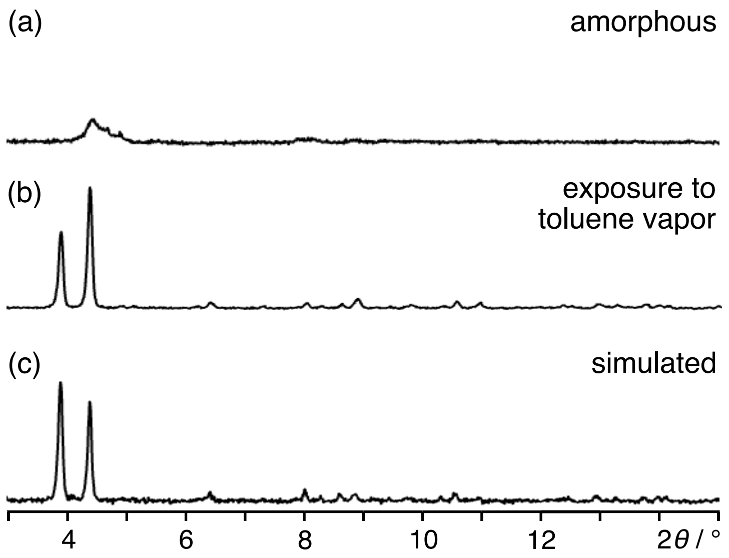

Figure S7. Powder X-ray diffraction patterns of (**2c**)$_6$ at 298 K. Diffraction patterns of (a) amorphous powders of **2c** prepared from a CH_2Cl_2 solution after evaporation of the solvent under reduced pressure, and then (b) exposure to toluene vapor for 1 h. (c) Simulated pattern of (**2c**)$_6$ based on its SXRD analysis. Intensities are shown as arbitrary units.

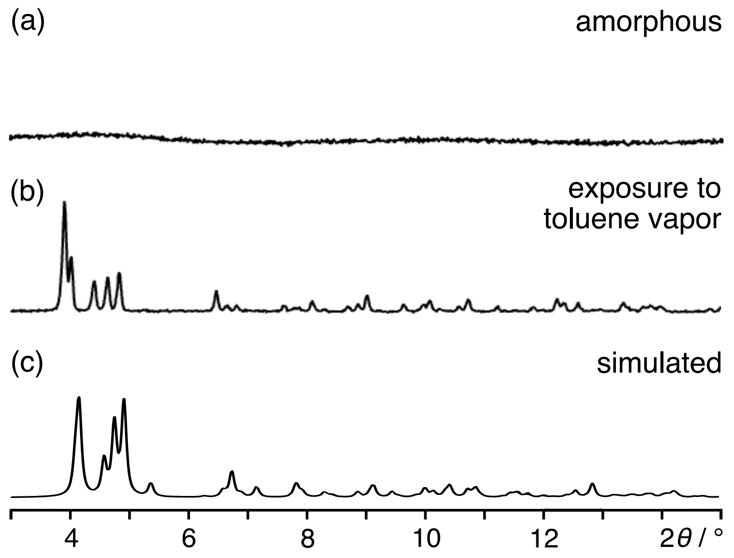

Figure S8. Powder X-ray diffraction patterns of (**2d**)$_6$ at 298 K. Diffraction patterns of (a) amorphous powders of **2d** prepared from a CH_2Cl_2 solution after evaporation of the solvent under reduced pressure, and then (b) exposure to toluene vapor for 12 h. (c) Simulated pattern of (**2d**)$_6$ based on its SXRD analysis. Intensities are shown as arbitrary units.

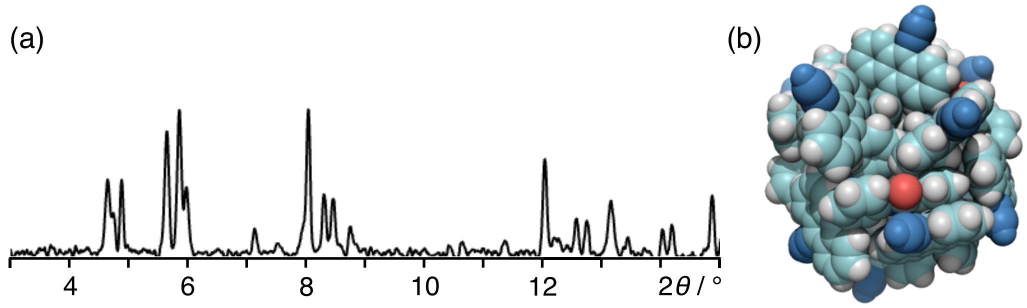

Figure S9. Powder X-ray diffraction patterns of crystalline powders of **2f** at 298 K. (a) A diffraction pattern of the crystalline powders of **2f** prepared from a toluene/*n*-hexane solution. Intensities are shown as arbitrary units. (b) CPK representation of molecular modeling of (**2f**)$_6$. Ethynyl groups were colored in dark blue.

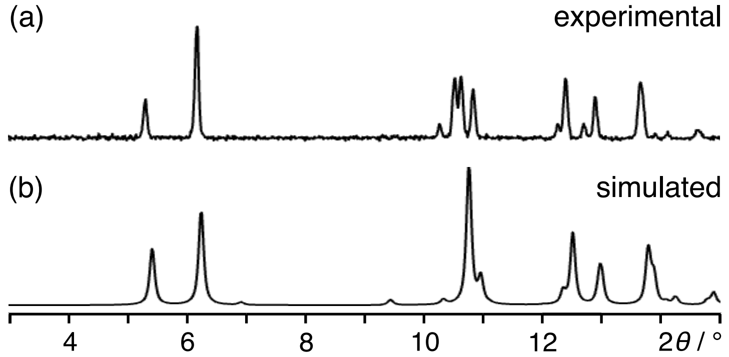

Figure S10. Powder X-ray diffraction patterns of (**2a**)$_n$ at 298 K. (a) A diffraction pattern of (**2a**)$_n$. (b) Simulated pattern of (**2a**)$_n$ based on its SXRD analysis. Intensities are shown as arbitrary units.

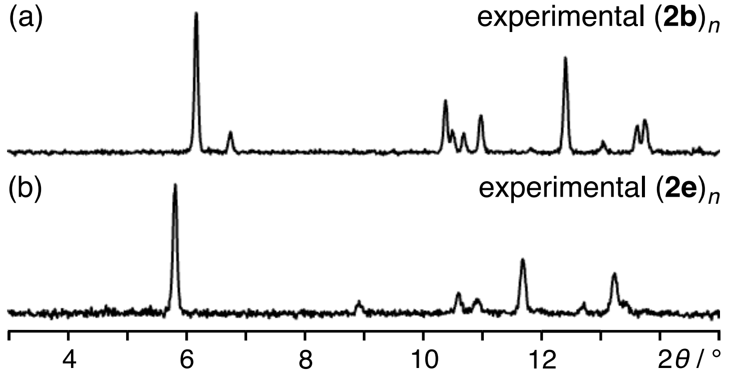

Figure S11. Powder X-ray diffraction patterns of (**2b**)$_n$ and (**2e**)$_n$ at 298 K. Diffraction patterns of (a) (**2b**)$_n$ and (b) (**2e**)$_n$. Intensities are shown as arbitrary units.

4. Solid-state physical properties

4.1. UV-vis and fluorescence spectra

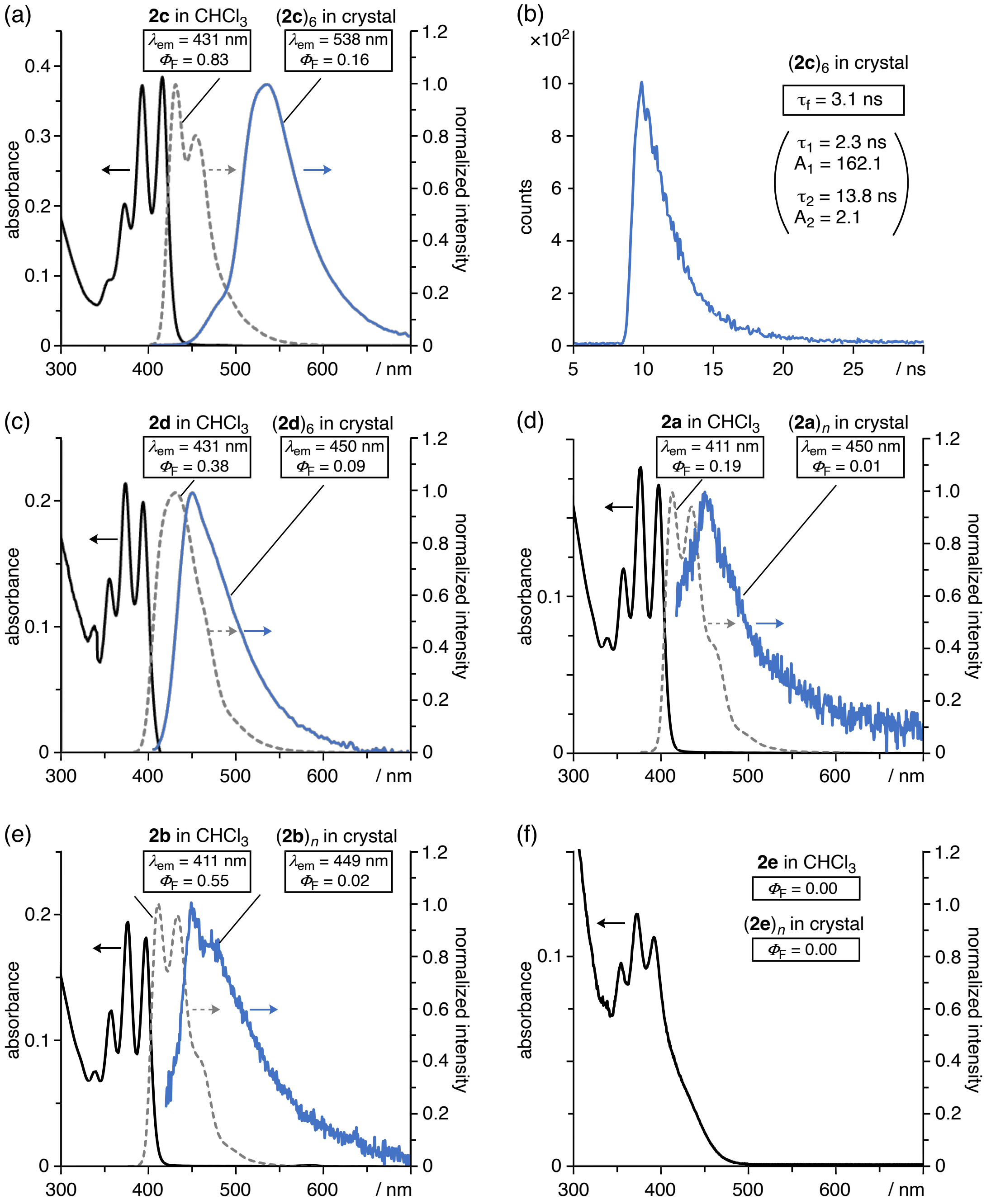

Figure S12. Photophysical properties. UV-vis spectra (black line) and fluorescence spectra (gray dash line: solution, blue line: solid-state) of (a) **2c** and $(\mathbf{2c})_6$, (c) **2d** and $(\mathbf{2d})_6$, (d) **2a** and $(\mathbf{2a})_n$, (e) **2b** and $(\mathbf{2b})_n$, and (f) **2e** and $(\mathbf{2e})_n$. Solution measurements were carried out in $CHCl_3$ (10 μM, 298 K) and the excited wavelengths for fluorescence analyses were utilized each maximum absorption of tweezers at 298 K. (b) Fluorescence lifetime decay curve of $(\mathbf{2c})_6$ in the solid state (monitored at 538 nm upon λ_{ex} = 405 nm, 298 K).

4.2. Thermal analysis

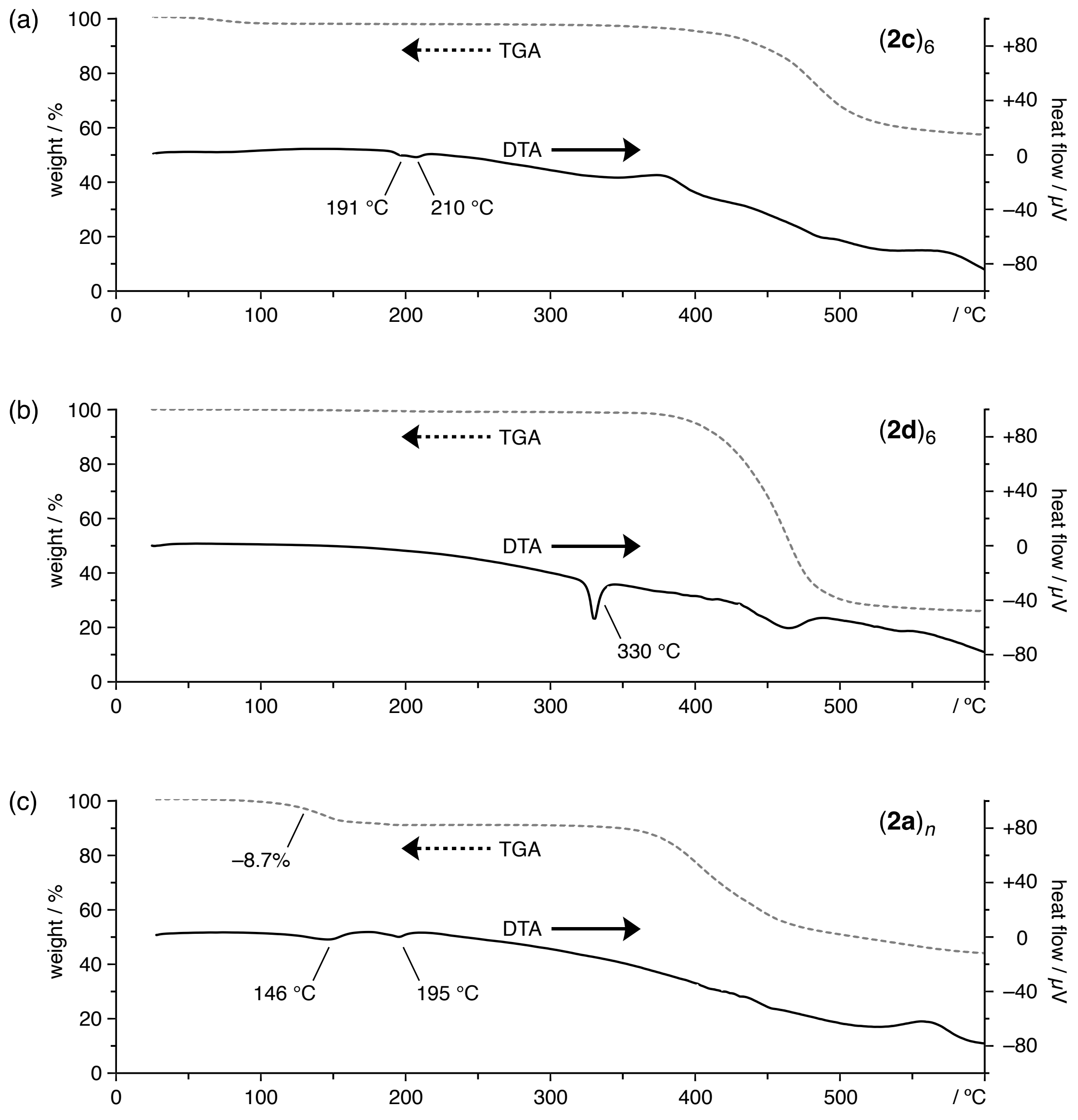

Figure S13. TG-DTA curves (heating rate of 10 K min^{-1} from 30 °C to 600 °C). (a) (**2c**)$_6$, (b) (**2d**)$_6$, and (c) (**2a**)$_n$ (TGA: dash line, DTA: solid line).

4.3. Hirshfeld surface analysis

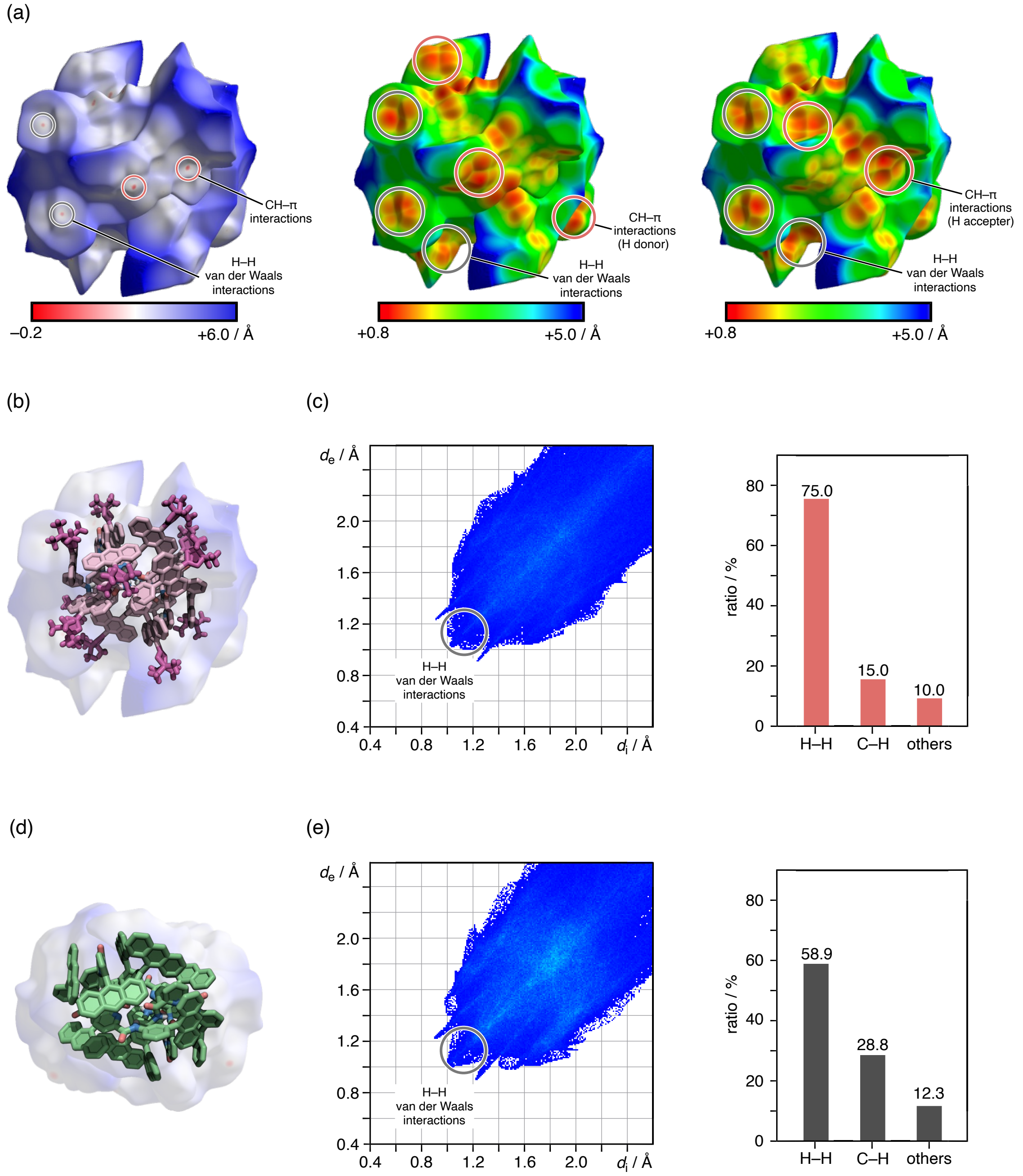

Figure S14. Hirshfeld surface analysis. Three-dimensional views of Hirshfeld surface mapped with (a) d_{norm}, (b) d_i, and (c) d_e of (**2d**)$_6$. The surface colors are utilized to visualize the atom–atom contacts: shorter than van der Waals contacts (red), equal to vdW (white or green), and longer than vdW (blue). Structural orientation of cyclic hexamers (b) (**2d**)$_6$ and (d) (**1**)$_6$ within their Hirshfeld surfaces. Hydrogen atoms are omitted for clarity. Fingerprint plots of (c) (**2d**)$_6$ and (e) (**1**)$_6$, and the ratio of their H–H, C–H, and other close contacts.

5. Formation of heterologous cyclic hexamers

5.1. Construction of heterologous cyclic hexamer KO-2-176

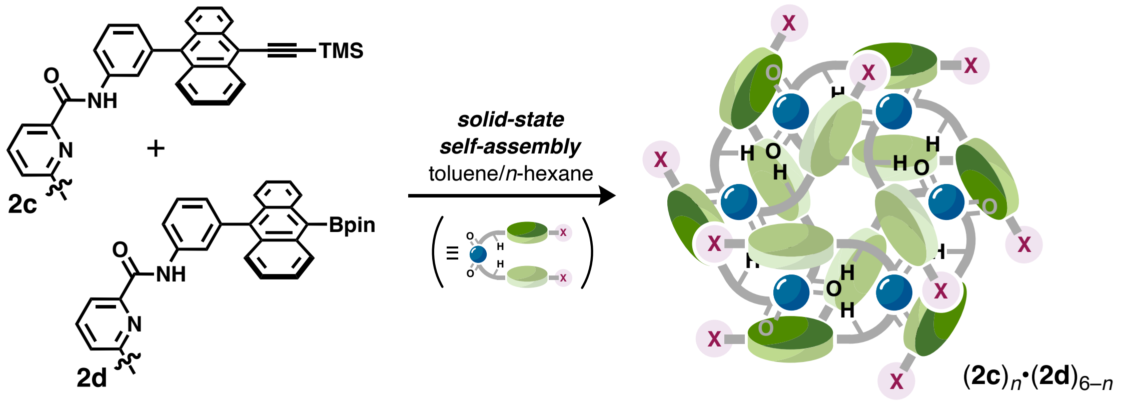

See experimental section in the manuscript for experimental procedure

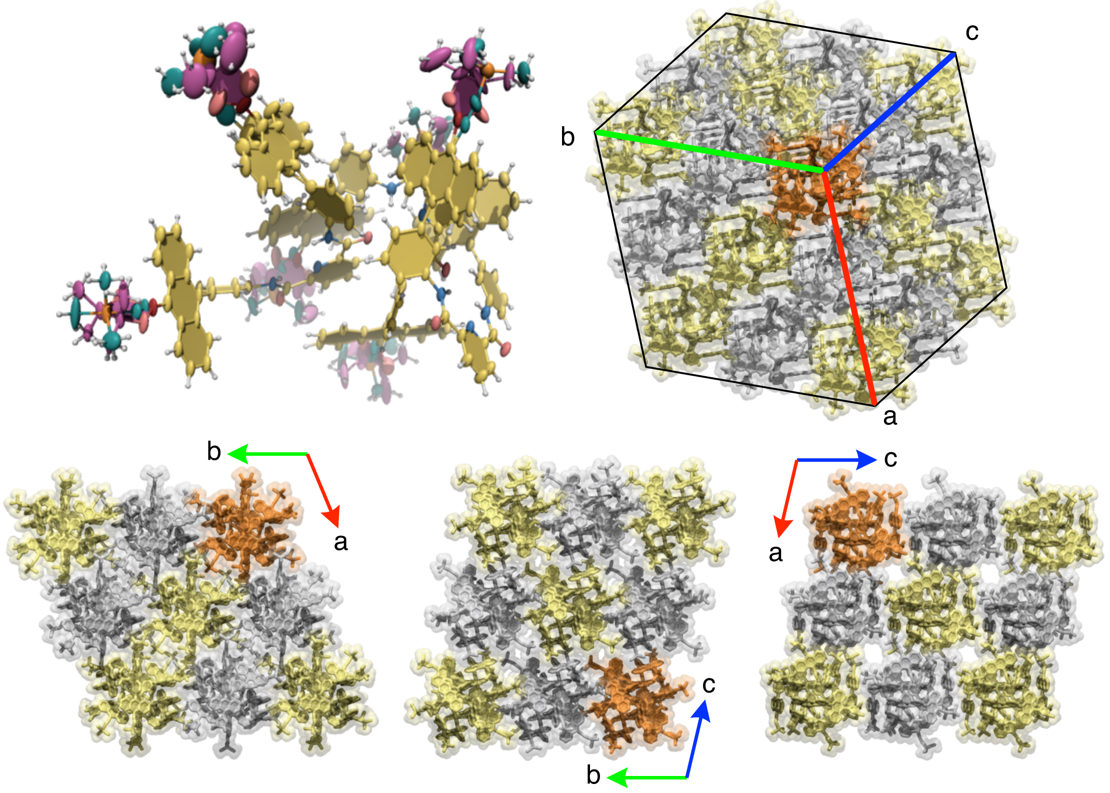

Figure S15. The ORTEP drawing of (**2c**)$_n$•(**2d**)$_{6-n}$ and its packing structure. The thermal ellipsoids are drawn at 30% possibility. Yellow: carbon, green: carbon (TMSethynyl), purple: carbon (Bpin), blue: nitrogen, pink: oxygen, orange: silicon, and red: boron.

Note:

There are two alerts A in the crystalline data of $(\mathbf{2c})_n$•$(\mathbf{2d})_{6-n}$ as below;

PLAT029_ALERT_3_A _diffrn_measured_fraction_theta_full value Low.

Because the measurement could perform only one axis rotation scan (omega scan with chi = 0) using the goniometer, we couldn't collect high completeness data for triclinic space group.

PLAT026_ALERT_3_A Ratio Observed / Unique Reflections (too) Low.

This is partly due to the severe disorder of the crystalline solvents, anthracene arms, and substituents of tweezers. In addition, as we discussed in the main text, the single crystal of $(\mathbf{2c})_n$•$(\mathbf{2d})_{6-n}$ was fragile in the oil owing to volatile solvents probably because of rapid exchanging crystalline solvents, leading to the loss of their single crystalline nature. Therefore, we applied the solvent mask implemented in Olex2.[S4]

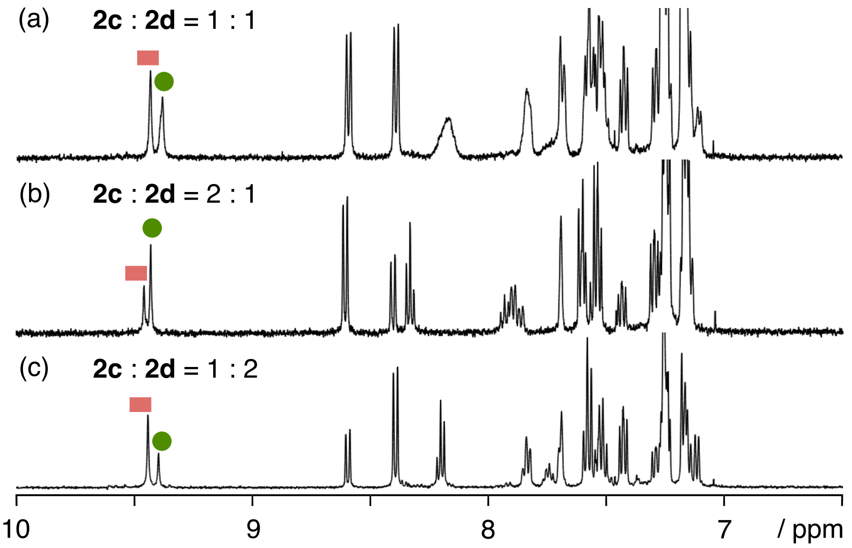

Figure S16. ^{1}H NMR spectra (500 MHz, $CDCl_3$, 298 K) of $(\mathbf{2c})_n$•$(\mathbf{2d})_{6-n}$. The crystals $(\mathbf{2c})_n$•$(\mathbf{2d})_{6-n}$ prepared from **2c** and **2d** in the ratio of (a) 1:1, (b) 2:1, and (c) 1:2 were filtrated, and excess tweezers molecules were removed by washing with toluene. After dissolving each crystal in $CDCl_3$, respective ^{1}H NMR spectra were measured (**2c**: green circle and **2d**: pink rectangle).

Table S2. Crystal data and structure refinement of (2c)$_n$•(2d)$_{6-n}$.

Compounds	**(2c)$_n$•(2d)$_{6-n}$**
Identification code	TMSBpin
CCDC number	2326659
Empirical formula	$C_{57.97}H_{49.91}B_{0.97}N_3O_{3.94}Si_{1.03}$
Formula weight	891.01
Temperature (K)	93
Wavelength (Å)	0.750
Crystal system	triclinic
Space group	P-1
Unit cell dimensions	
a (Å)	21.190(6)
b (Å)	21.218(5)
c (Å)	22.724(5)
α (°)	97.074(10)
β (°)	100.503(16)
γ (°)	111.528(5)
Volume (Å^3)	9141(4)
Z	6
Density (calculated)	0.971
Absorption coefficient	0.090
F(000)	2817.0
Crystal size	0.1 × 0.1 × 0.1
2θ range for data collection	1.966 to 71.044
	$-32 \leq h \leq 32$
Index ranges	$-30 \leq k \leq 31$
	$-28 \leq l \leq 26$
Reflections collected	100204
Independent reflections	56749 [R_{int} = 0.0815]
Completeness to θ = 35.522°	0.899
Absorption correction	multi-scan
Refinement method	Full-matrix least-squares on F^2
Data / restraints / parameters	56749/647/2258
Goodness-of-fit on F^2	0.899
Final R indices [I>2sigma(I)]	R_1 = 0.0994
	wR_2 = 0.2727
R indices (all data)	R_1 = 0.2425
	wR_2 = 0.3709
Largest diff. peak and hole	0.51 and -0.42 e Å^{-3}

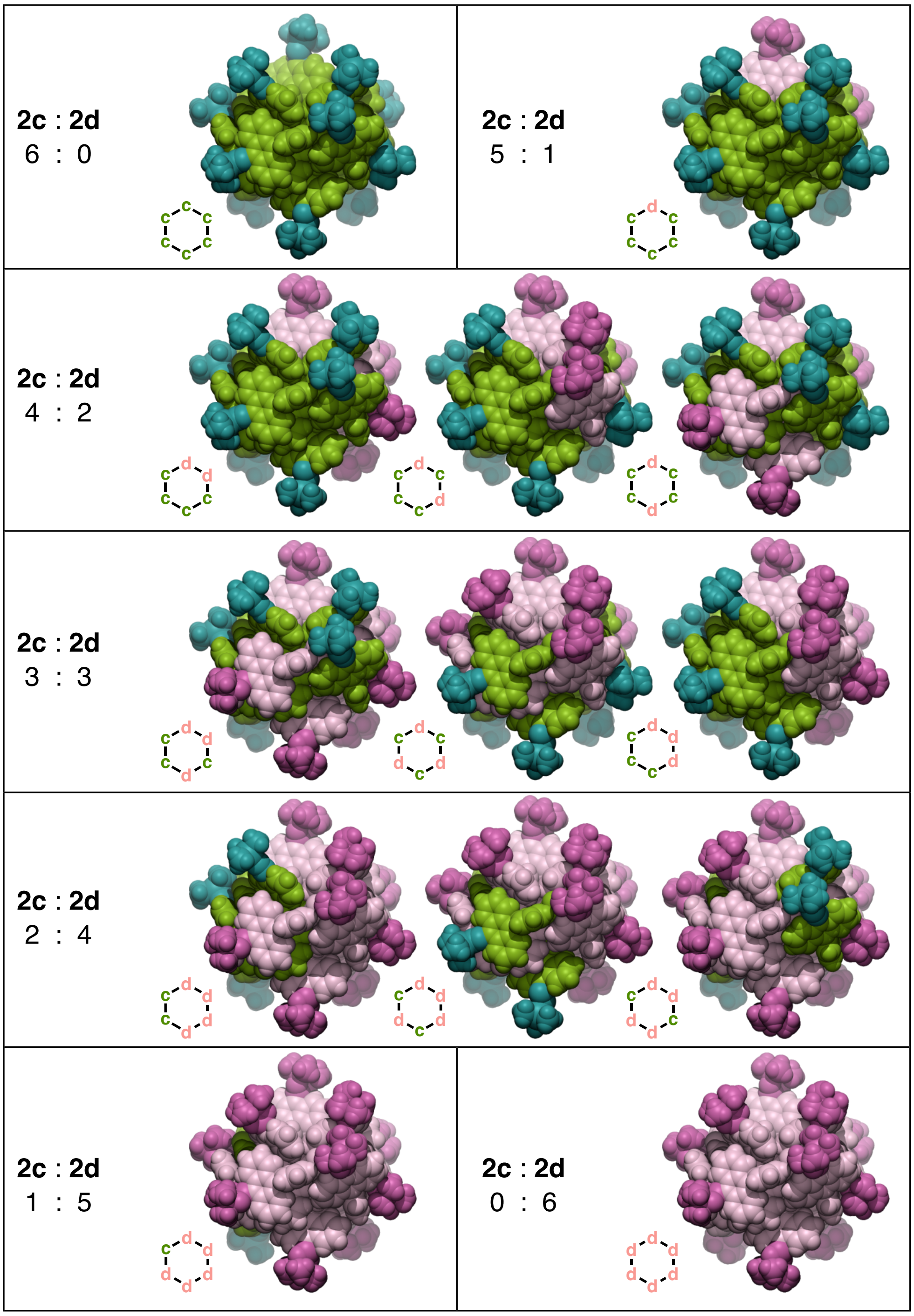

Figure S17. CPK representation of heterologous cyclic hexamers. All of possible structures $(\mathbf{2c})_n \bullet (\mathbf{2c})_{6-n}$ (n = 1–6) were estimated from the result of SXRD analysis. Molecular tweezers **2c** and **2d** were colored in green and pink, respectively.

5.2. Solid-state physical properties

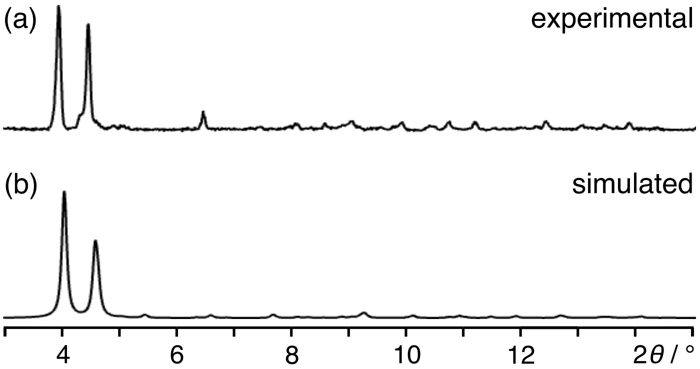

Figure S18. Powder X-ray diffraction patterns of $(\mathbf{2c})_n$•$(\mathbf{2d})_{6-n}$ at 298 K. (a) A diffraction pattern of $(\mathbf{2c})_n$•$(\mathbf{2d})_{6-n}$ and (b) its simulated pattern based on the SXRD analysis. Intensities are shown as arbitrary units.

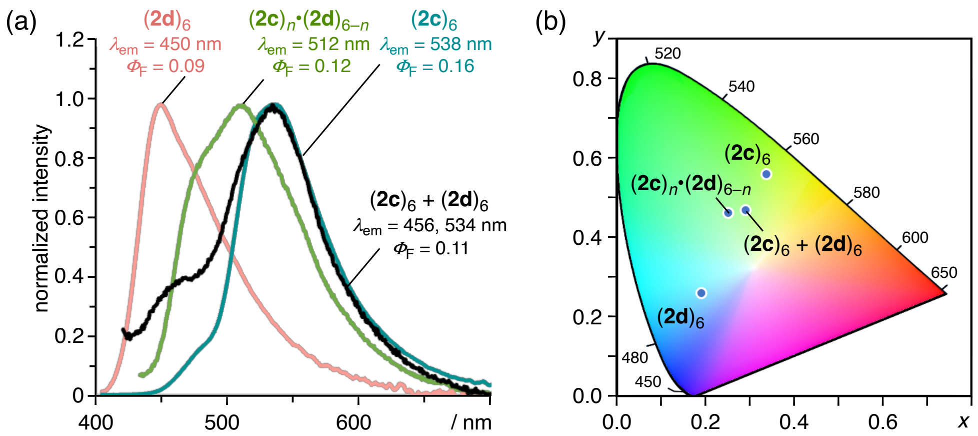

Figure S19. Comparison of fluorescent spectra of each hexamer. Fluorescence spectra of $(\mathbf{2c})_2$ (blue line), $(\mathbf{2d})_6$ (pink line), $(\mathbf{2c})_n$•$(\mathbf{2d})_{6-n}$ (green line), and $(\mathbf{2c})_6$ + $(\mathbf{2d})_6$ (black line) and (b) their CIE coordinate diagram (λ_{ex} = 393 nm, 298 K).

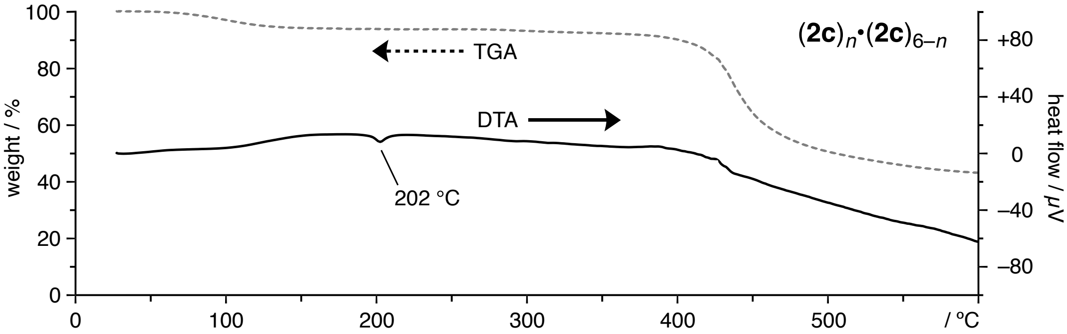

Figure S20. TG-DTA curves (heating rate of 10 K min^{-1} from 30 °C to 600 °C) of $(\mathbf{2c})_6$•$(\mathbf{2d})_{6-n}$ (TGA: dash line, DTA: solid line).

6. Dynamic Interconversion

6.1. Construction of pseudo cyclic dimer

KO-2-175

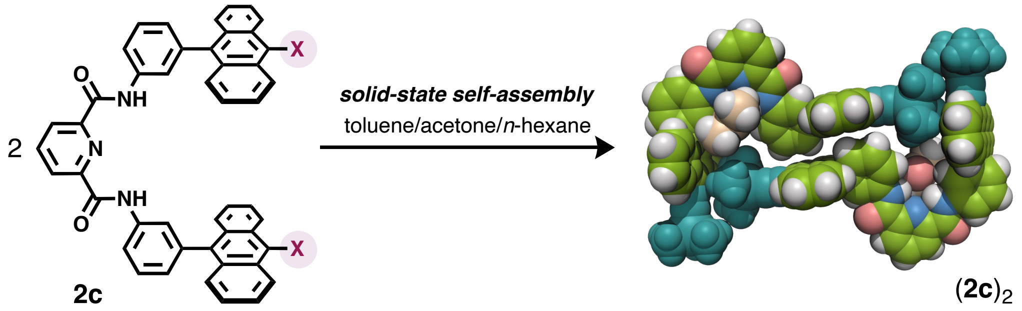

See experimental section in the manuscript for experimental procedure

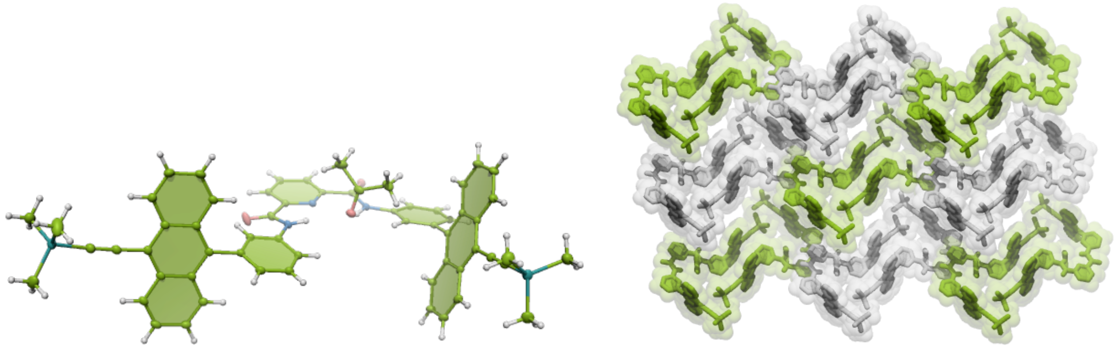

Figure S21. The ORTEP drawing structures of (**2c**)$_2$ and its packing structure. The thermal ellipsoids are drawn at 50% possibility. Green: carbon, blue: nitrogen, pink: oxygen, and dark green: silicon.

Table S3. Crystal data and structure refinement of (2c)$_2$.

Compounds	**(2c)**$_2$
Identification code	KO-TMSacetone-Tolhex
CCDC number	2326661
Empirical formula	$C_{60}H_{53}N_3O_3Si_2$
Formula weight	920.23
Temperature (K)	123
Wavelength (Å)	1.54184
Crystal system	triclinic
Space group	P-1
Unit cell dimensions	
a (Å)	8.64460(10)
b (Å)	14.32220(10)
c (Å)	21.5107(2)
α (°)	79.3420(10)
β (°)	84.4380(10)
γ (°)	74.1810(10)
Volume (Å^3)	2515.12(4)
Z	2
Density (calculated)	1.215
Absorption coefficient	1.018
F(000)	972.0
Crystal size	0.251 × 0.133 × 0.082
2θ range for data collection	4.184 to 148.994
	$-10 \leq h \leq 10$
Index ranges	$-17 \leq k \leq 17$
	$-26 \leq l \leq 26$
Reflections collected	48543
Independent reflections	10234 [Rint = 0.0266]
Completeness to θ = 74.497°	0.997
Absorption correction	multi-scan
Refinement method	Full-matrix least-squares on F^2
Data / restraints / parameters	10234/0/621
Goodness-of-fit on F^2	1.035
Final *R* indices [I>2sigma(I)]	R_1 = 0.0398
	wR_2 = 0.1090
R indices (all data)	R_1 = 0.0450
	wR_2 = 0.1155
Largest diff. peak and hole	0.30 and -0.40 e Å^{-3}

6.2. Solid-state physical properties of pseudo cyclic dimer

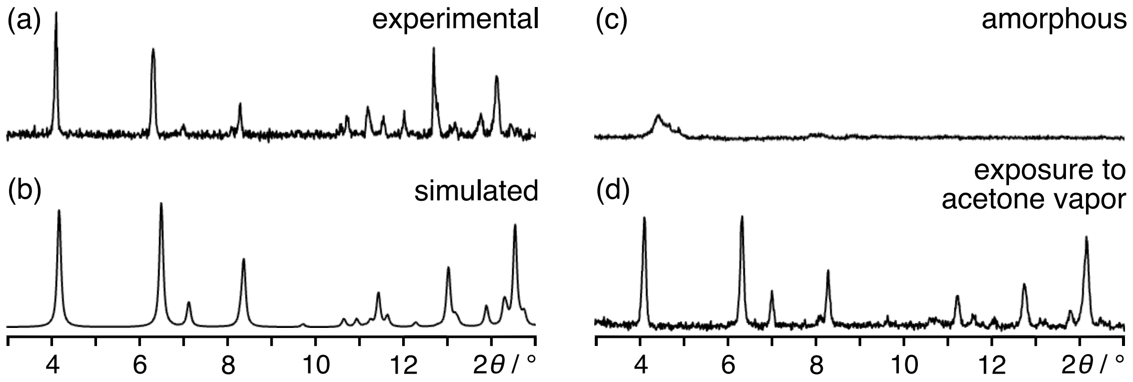

Figure S22. Powder X-ray diffraction patterns of (**2c**)$_2$ acetone adduct at 298 K. Diffraction patterns of (a) crystalline powders of (**2c**)$_2$, (b) simulated pattern, (c) amorphous powders and then (d) exposure to acetone vapor for 12 h. Intensities are shown as arbitrary units.

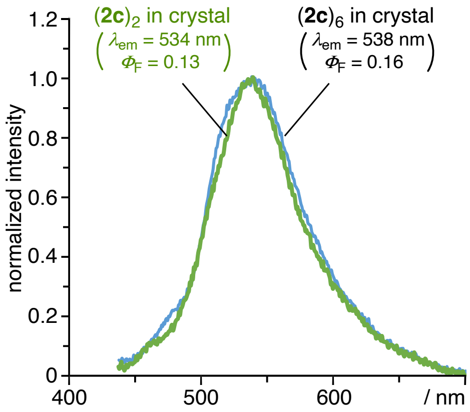

Figure S23. Solid-state fluorescence spectra of (**2c**)$_2$. Fluorescence spectra of (**2c**)$_2$ (green line) and (**2c**)$_6$ (blue line) with their quantum yields (λ_{ex} = 393 nm, 298 K).

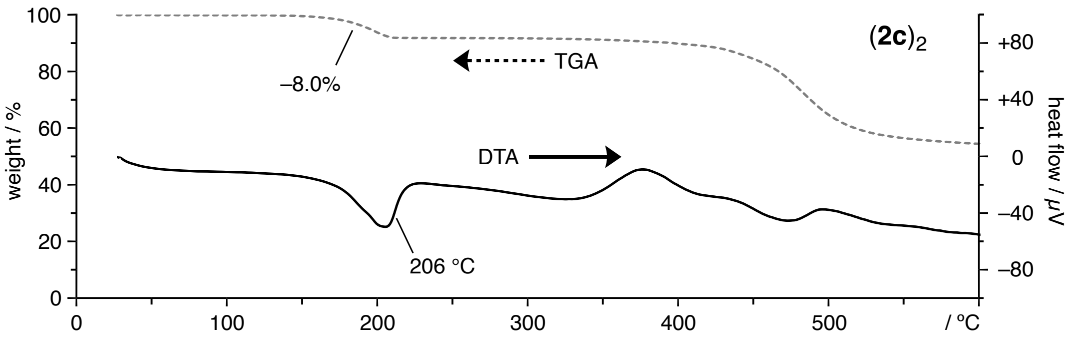

Figure S24. TG-DTA curves (heating rate of 10 K min^{-1} from 30 °C to 600 °C) of (**2c**)$_2$ (TGA: dash line, DTA: solid line).

6.3. Dynamic interconversion in the solid state

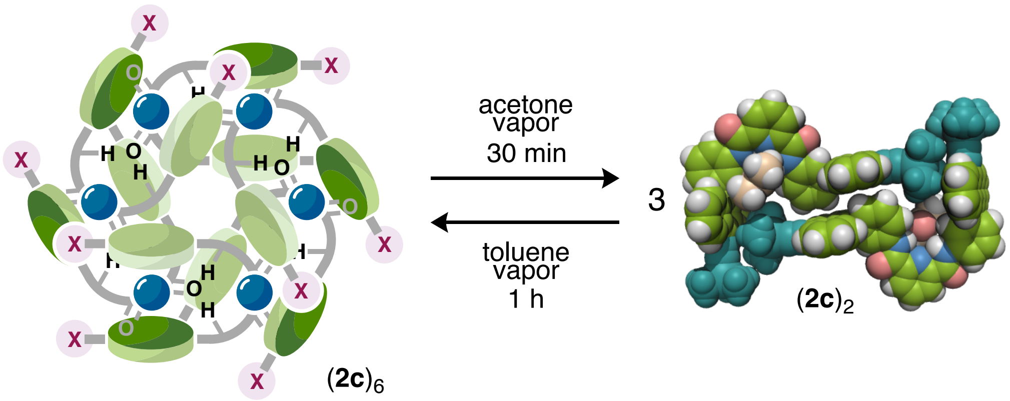

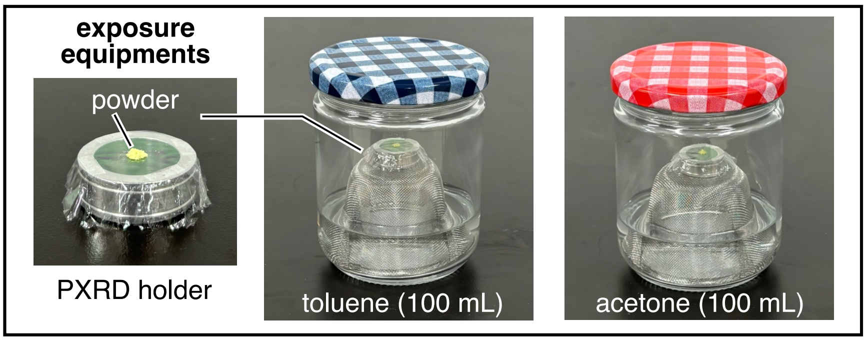

See experimental section in the manuscript for experimental procedure

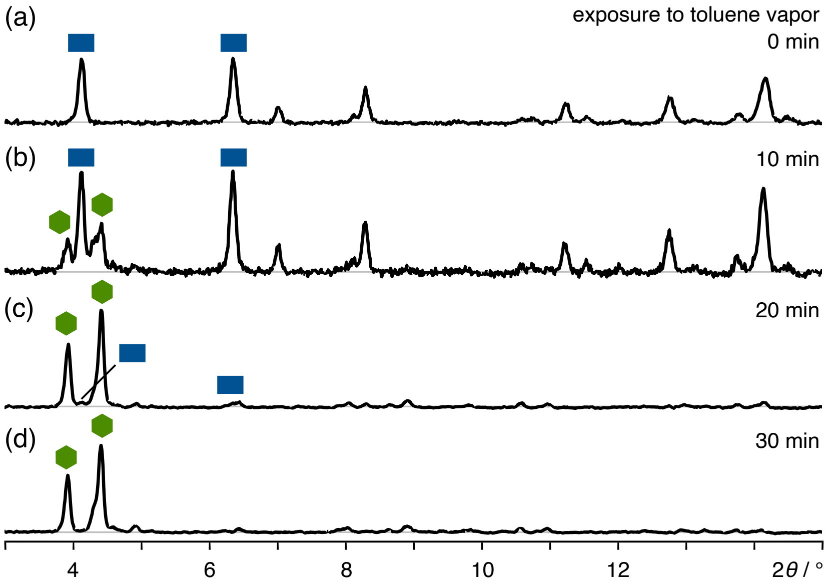

Figure S25. Time-course PXRD measurements of (**2c**)$_2$. Crystalline powders of (**2c**)$_2$ were exposed to toluene vapor at 298 K (blue rectangle: (**2c**)$_2$ and green hexagon: (**2c**)$_6$).

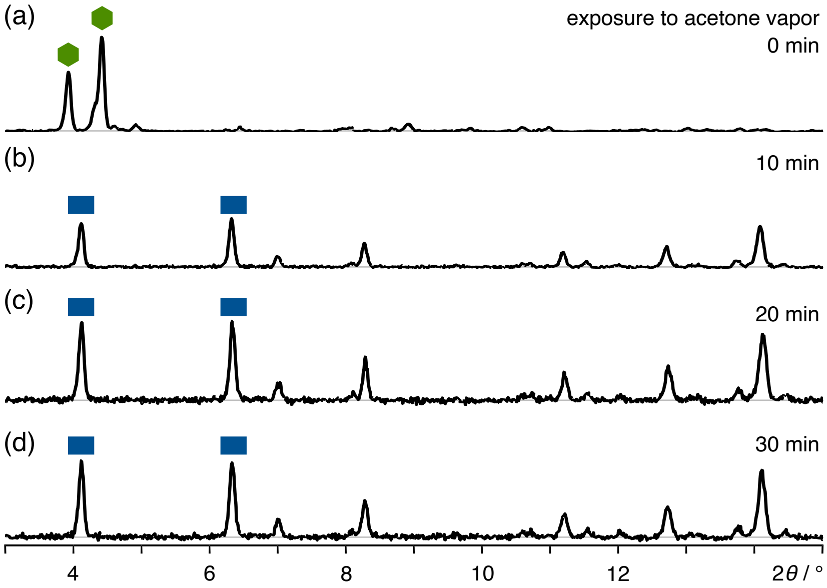

Figure S26. Time-course PXRD measurements of (**2c**)$_6$. Crystalline powders of (**2c**)$_6$ were exposed to acetone vapor at 298 K (blue rectangle: (**2c**)$_2$ and green hexagon: (**2c**)$_6$).

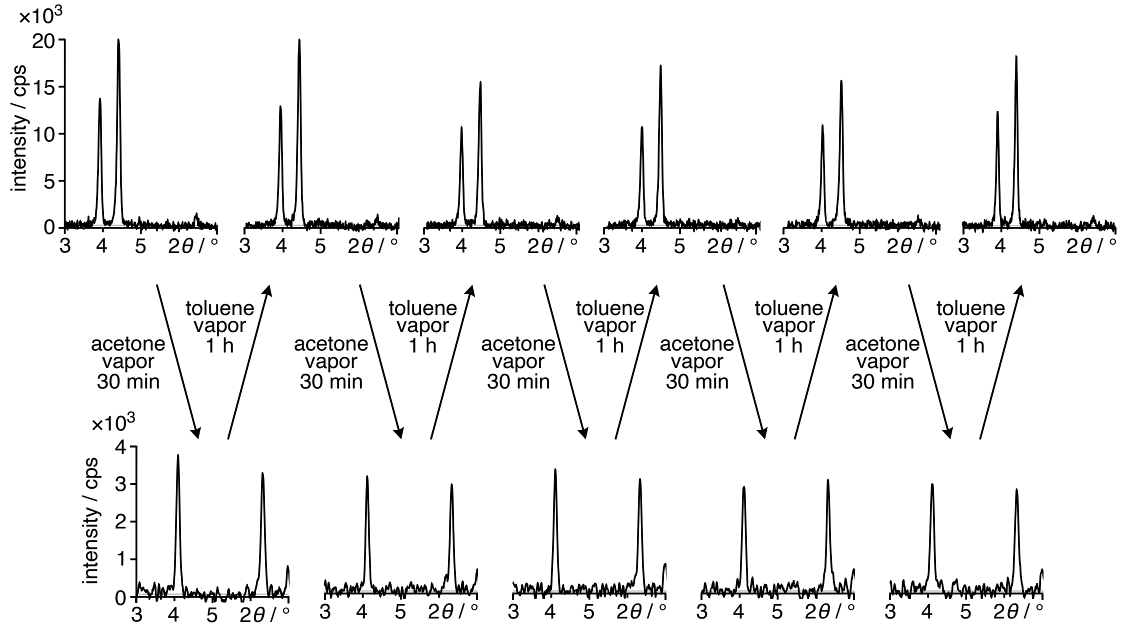

Figure S27. Reversible tests of crystal-to-crystal interconversion between (**2c**)$_6$ and (**2c**)$_2$ at 298 K. PXRD analysis was carried out after exposure the crystalline powders to acetone vapor for 30 min or toluene vapor for 1 h during each measurement.

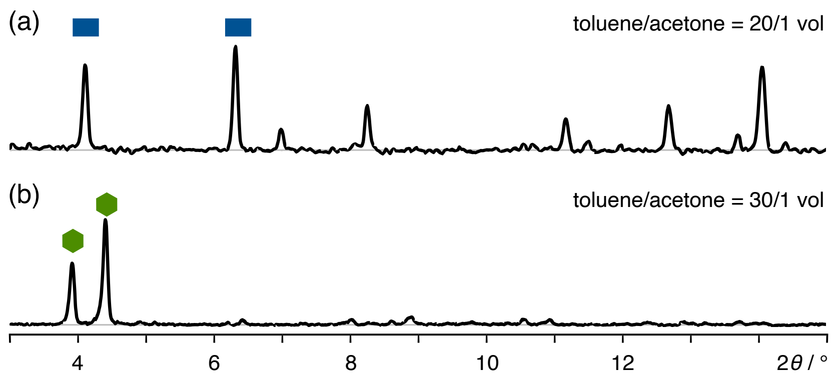

Figure S28. PXRD measurements of amorphous powders of **2c**. After exposure amorphous powders of **2c** to a mixture of toluene and acetone vapor in the ratio of (a) 20/1 vol and (b) 30/1 at 298 K for 12 h (blue rectangle: (**2c**)$_2$ and green hexagon: (**2c**)$_6$).

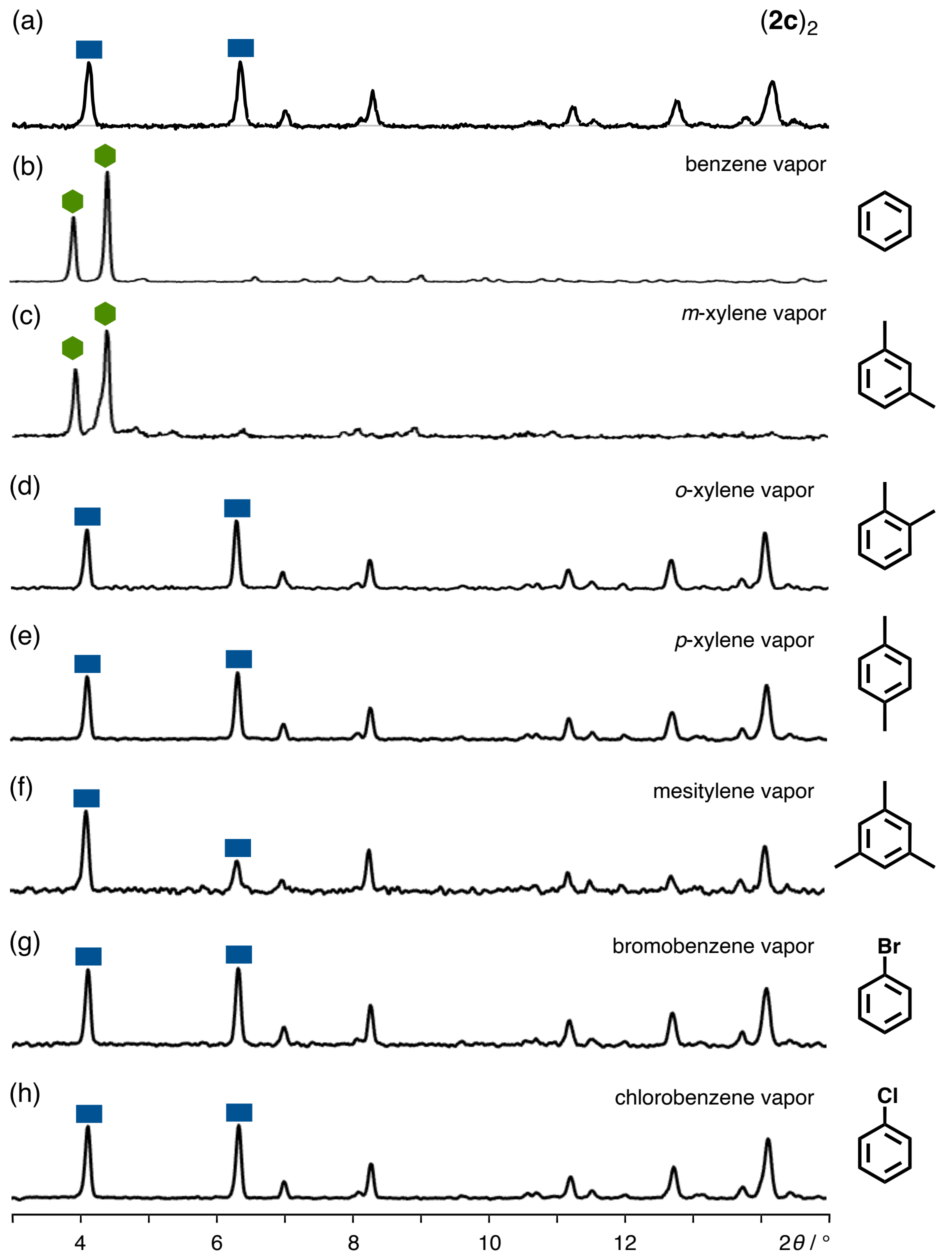

Figure S29. Solvent-dependent of interconversion from (**2c**)$_2$ to (**2c**)$_6$. PXRD diffraction patterns of (a) (**2a**)$_2$, and after exposure to (b) benzene vapor, (c) *m*-xylene vapor, (d) *o*-xylene vapor, (e) *p*-xylene vapor, (f) mesitylene vapor, (g) bromobenzene vapor and (b) chlorobenzene vapor at 298 K for 12 h (blue rectangle: (**2c**)$_2$ and green hexagon: (**2c**)$_6$).

7. NMR and MS spectra

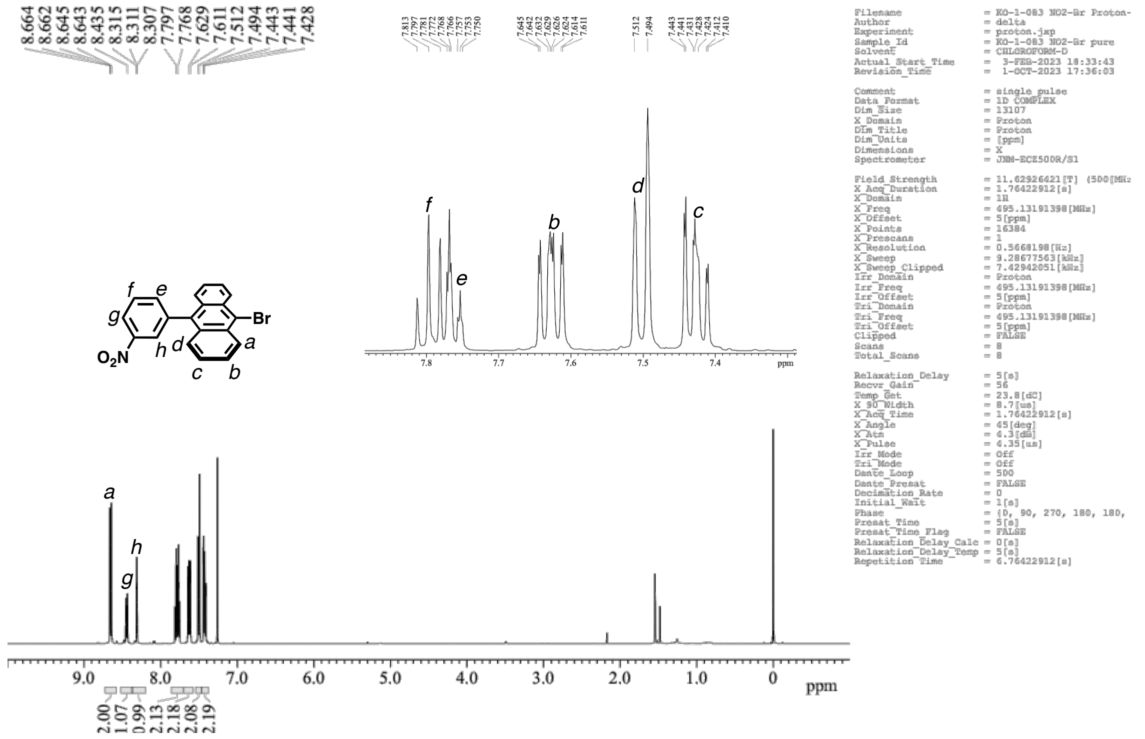

Figure S30. ^{1}H NMR spectrum (500 MHz, $CDCl_3$, 298 K) of **3a**.

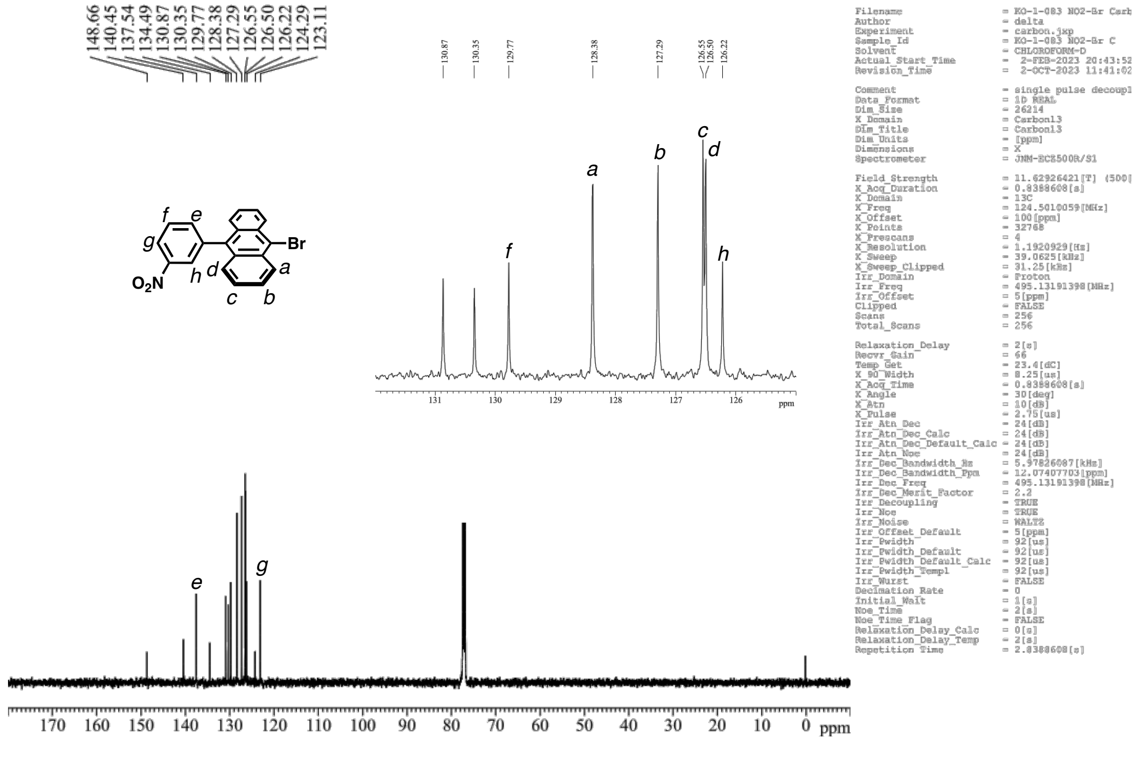

Figure S31. $^{13}C\{^{1}H\}$ NMR spectrum (125 MHz, $CDCl_3$, 298 K) of **3a**.

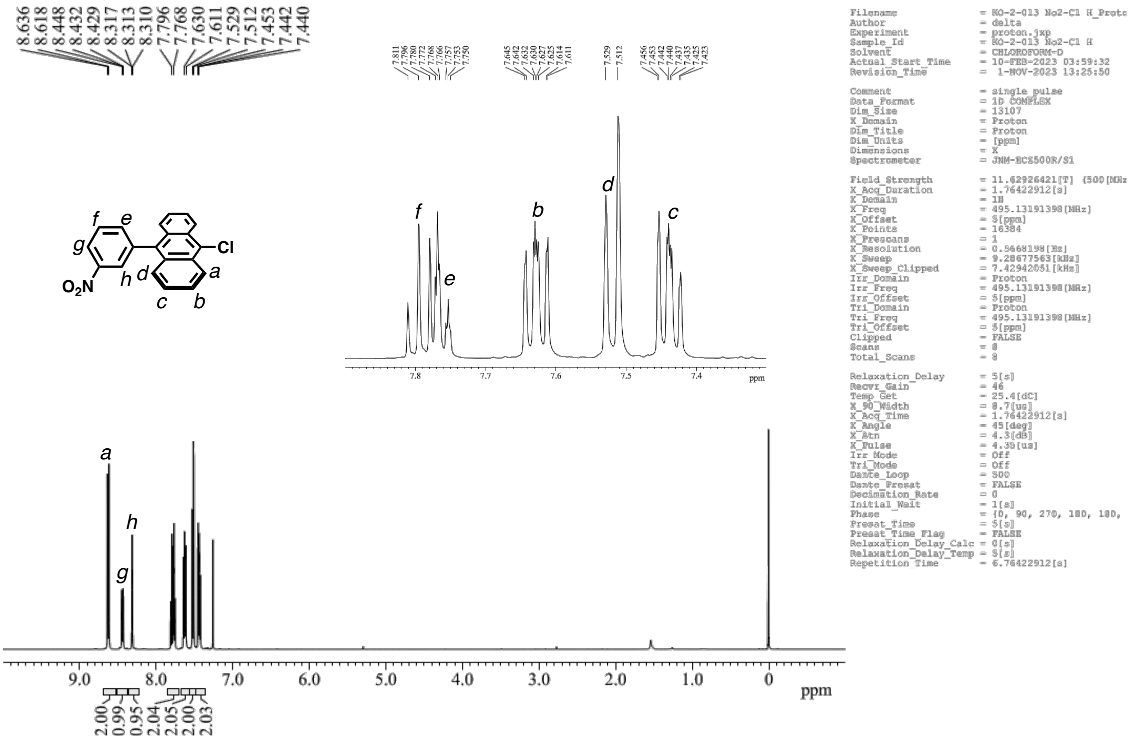

Figure S32. ^{1}H NMR spectrum (500 MHz, $CDCl_3$, 298 K) of **3b**.

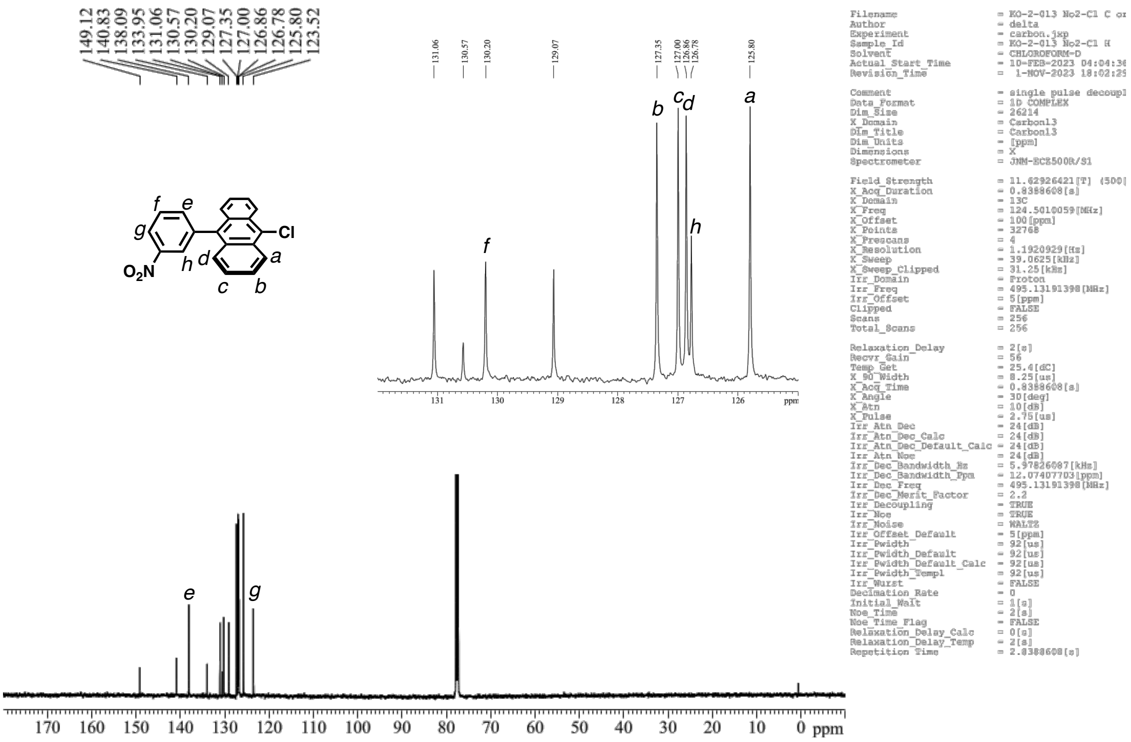

Figure S33. $^{13}C\{^{1}H\}$ NMR spectrum (125 MHz, $CDCl_3$, 298 K) of **3b**.

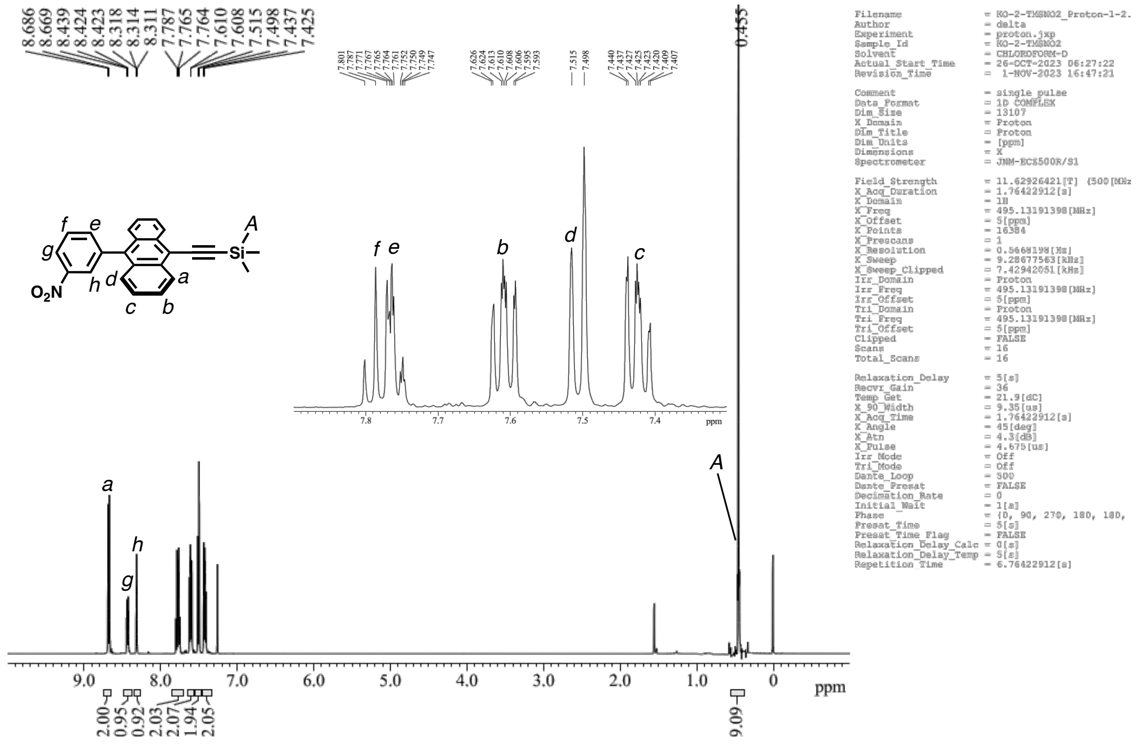

Figure S34. ^{1}H NMR spectrum (500 MHz, $CDCl_3$, 298 K) of **3c**.

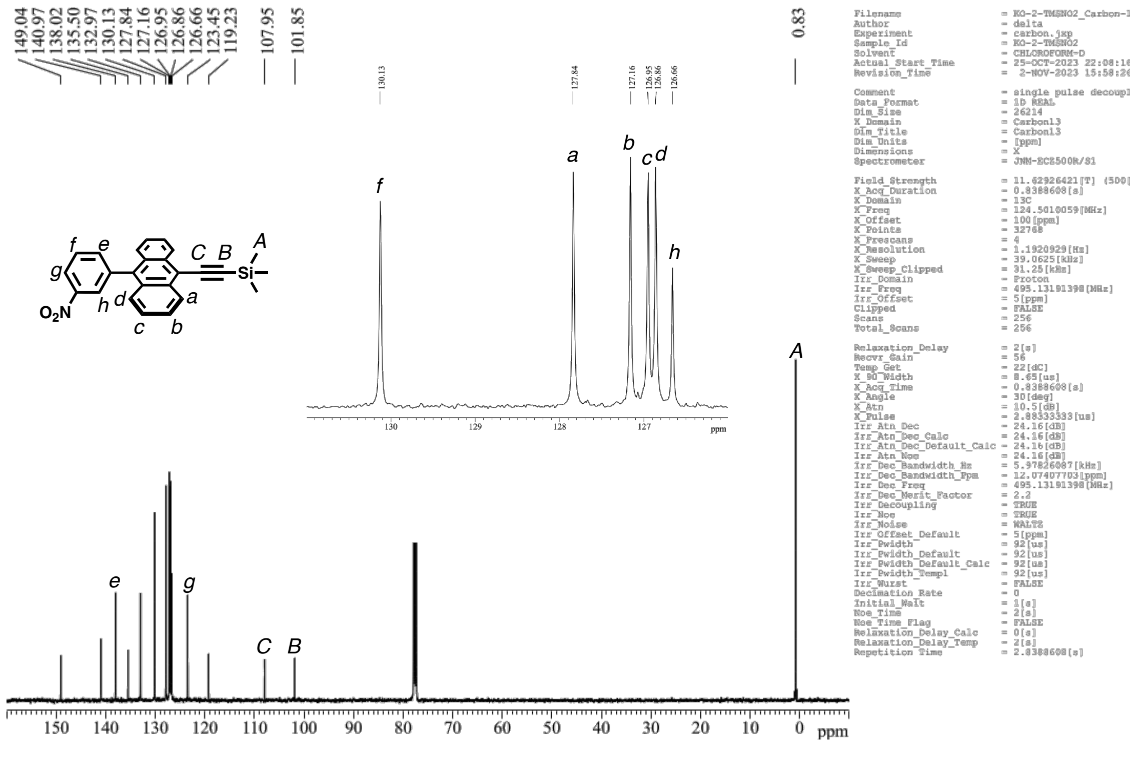

Figure S35. $^{13}C\{^{1}H\}$ NMR spectrum (125 MHz, $CDCl_3$, 298 K) of **3c**.

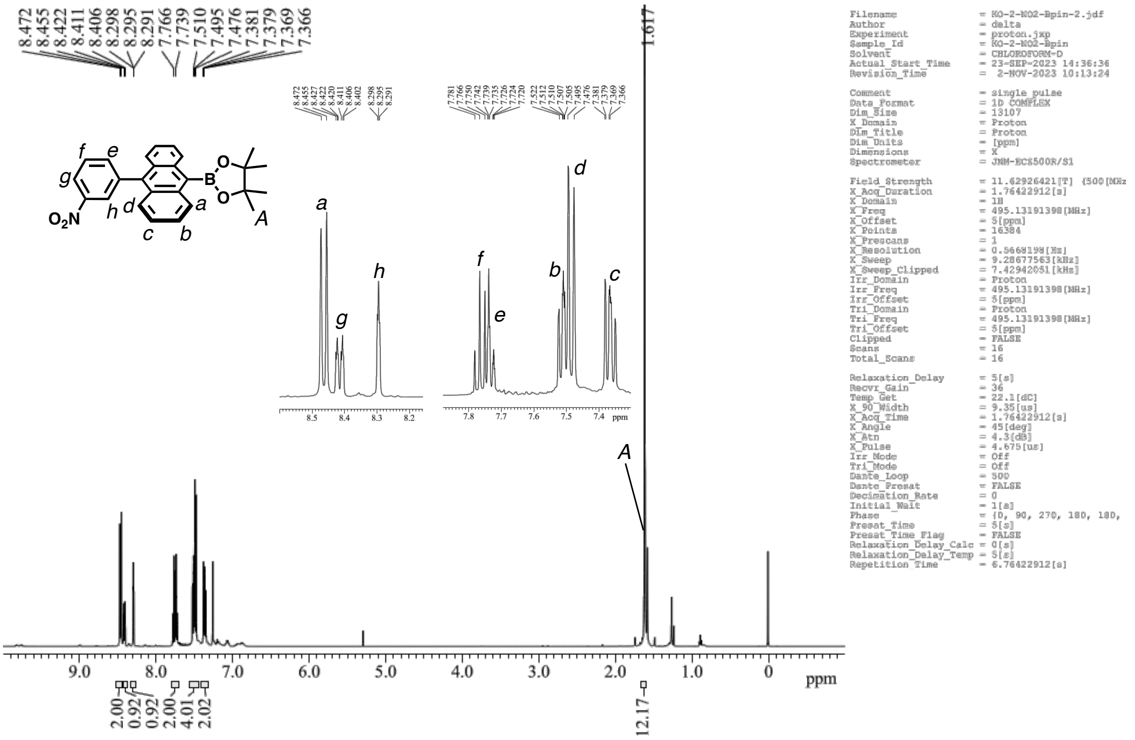

Figure S36. ^{1}H NMR spectrum (500 MHz, $CDCl_3$, 298 K) of **3d**.

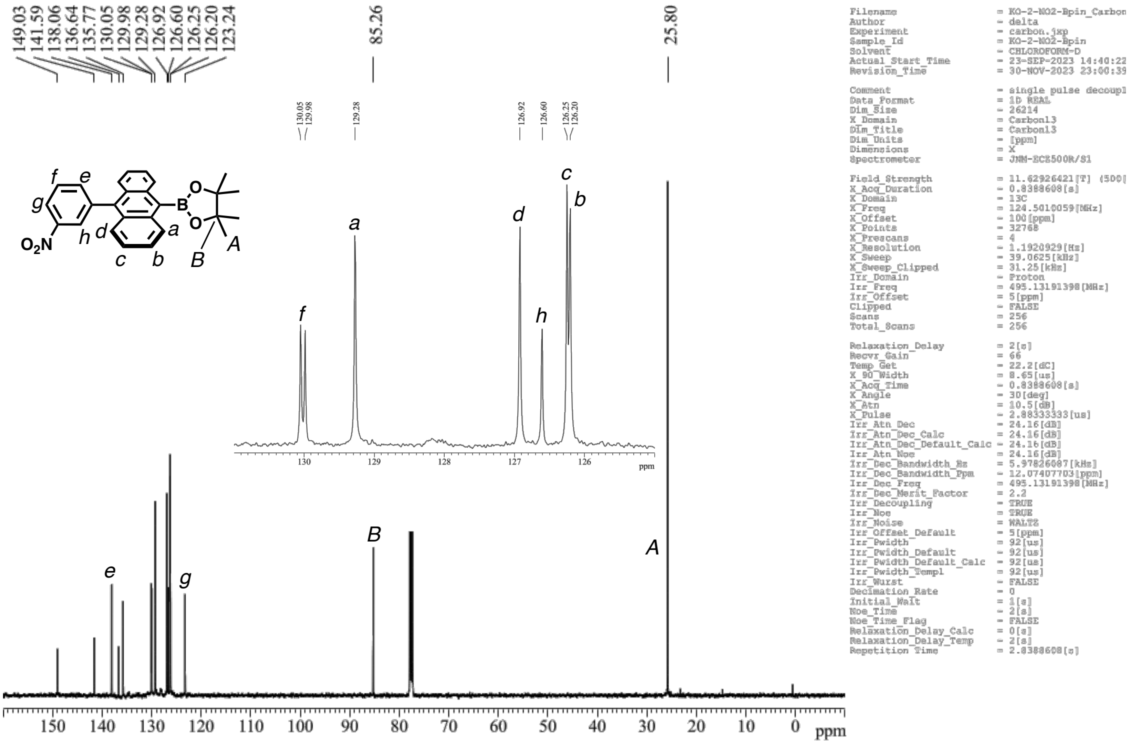

Figure S37. $^{13}C\{^{1}H\}$ NMR spectrum (125 MHz, $CDCl_3$, 298 K) of **3d**.

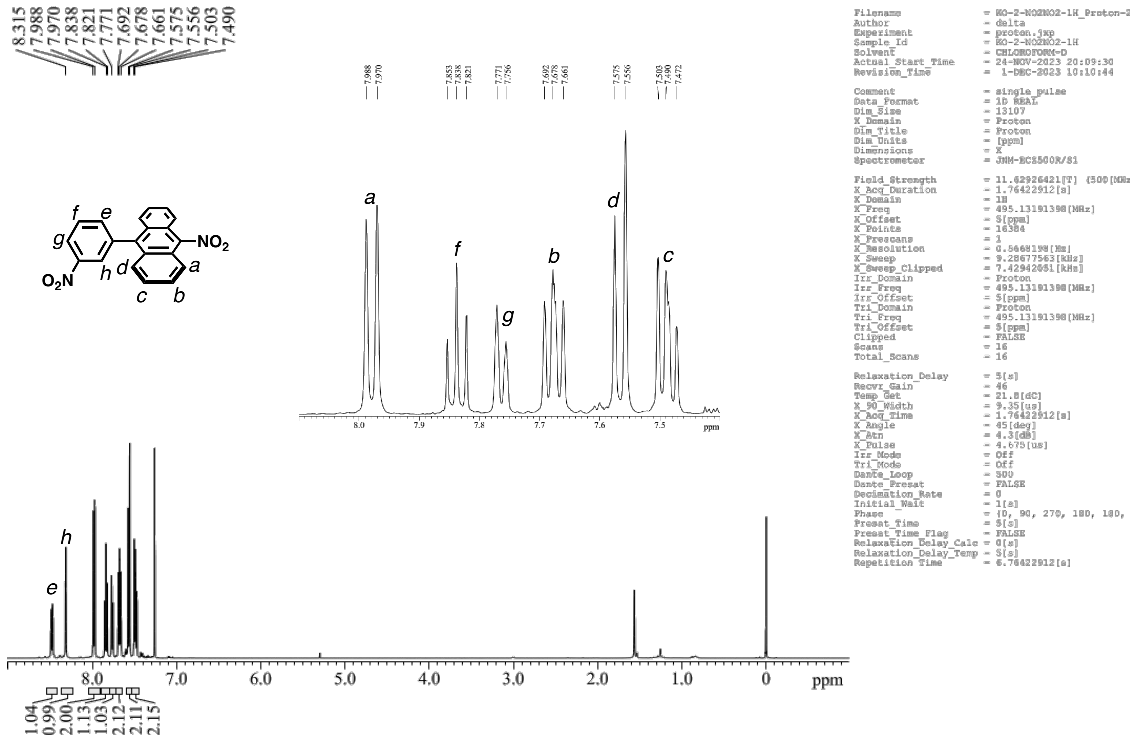

Figure S38. ^{1}H NMR spectrum (500 MHz, $CDCl_3$, 298 K) of **3e**.

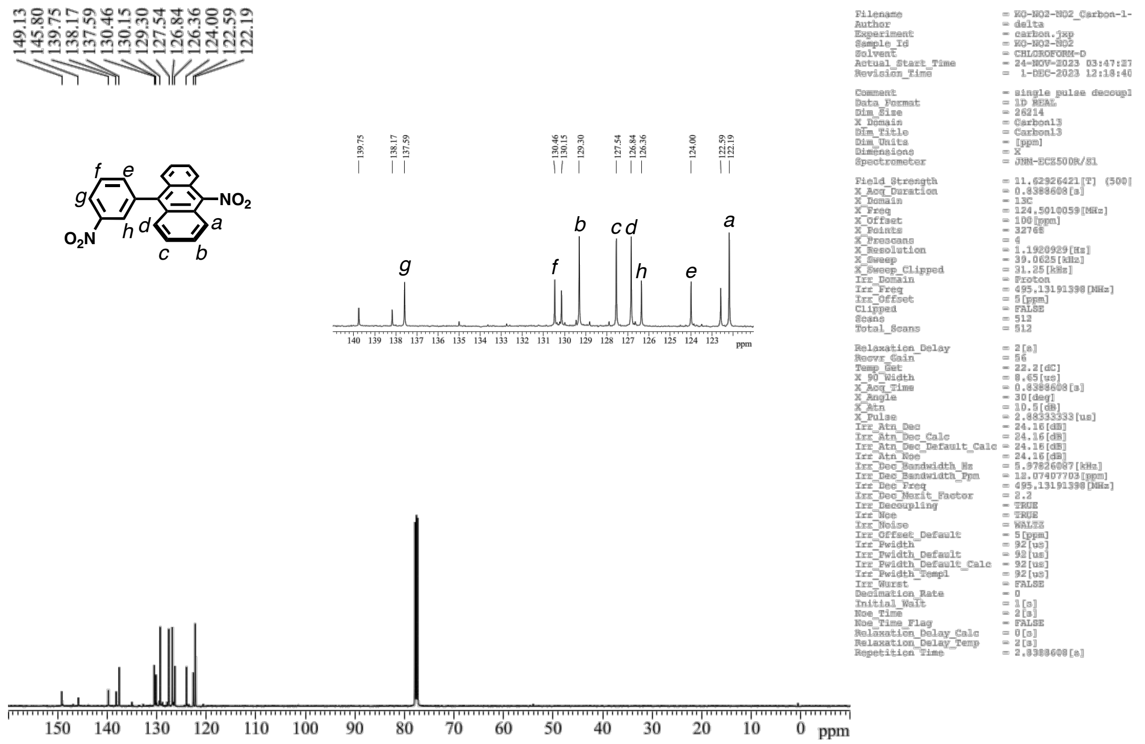

Figure S39. $^{13}C\{^{1}H\}$ NMR spectrum (125 MHz, $CDCl_3$, 298 K) of **3e**.

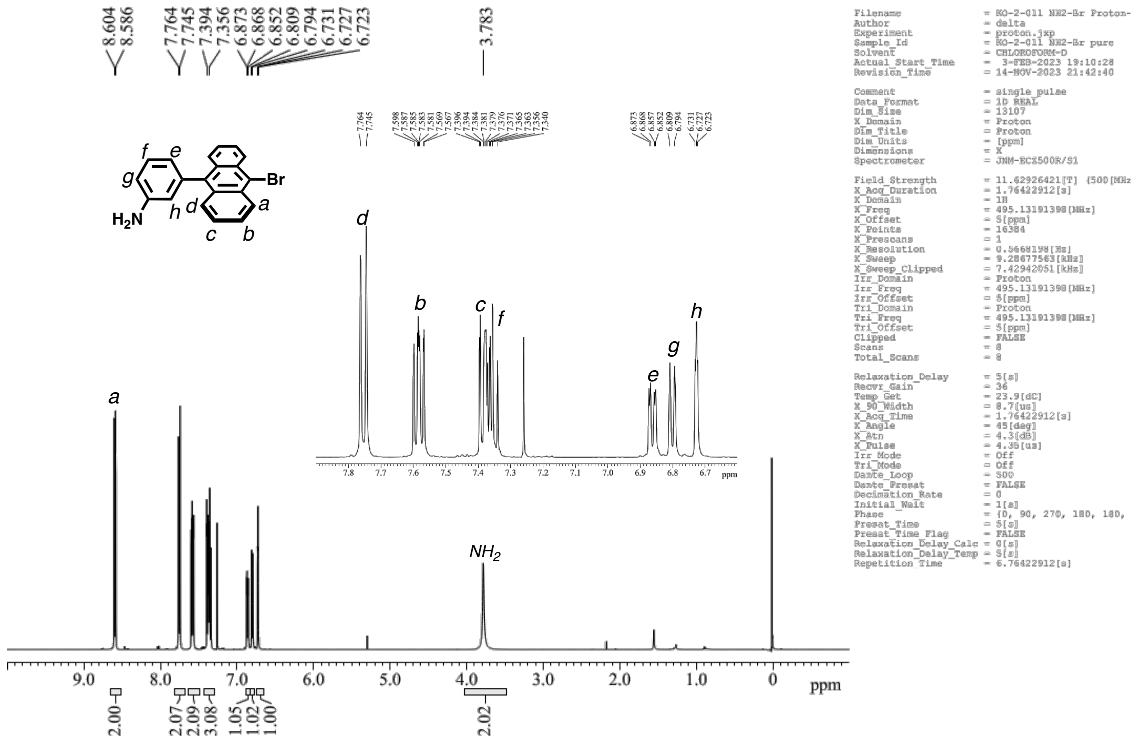

Figure S40. ^{1}H NMR spectrum (500 MHz, $CDCl_3$, 298 K) of **4a**.

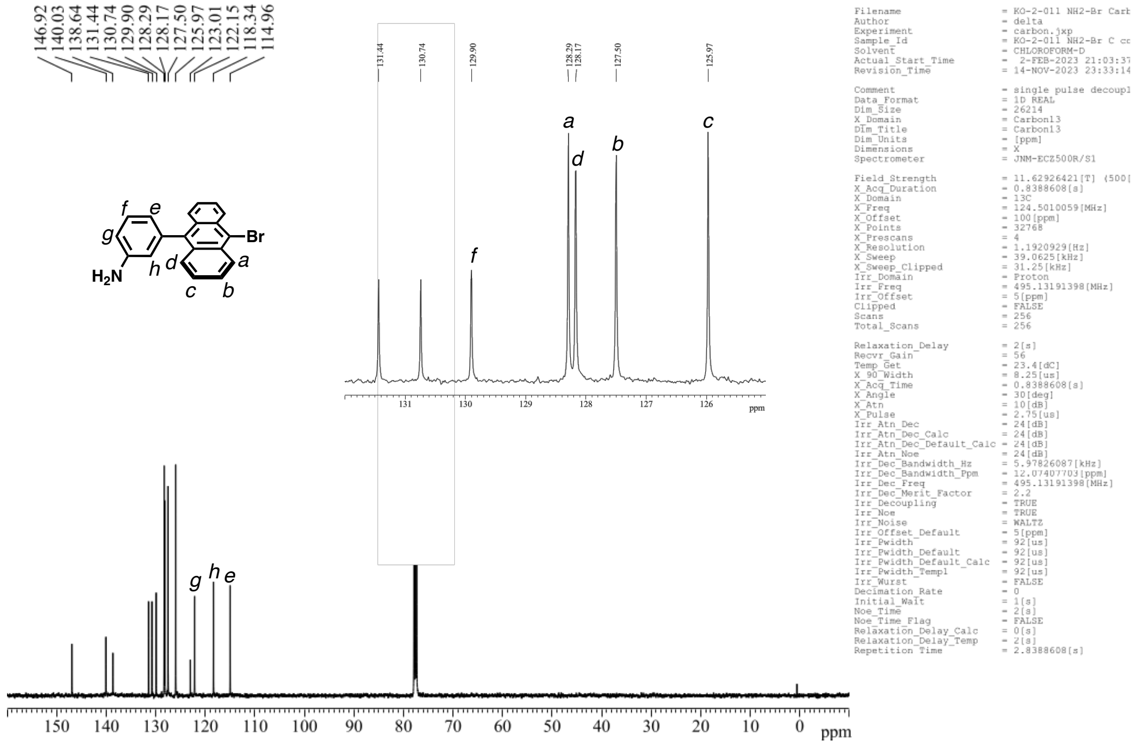

Figure S41. $^{13}C\{^{1}H\}$ NMR spectrum (125 MHz, $CDCl_3$, 298 K) of **4a**.

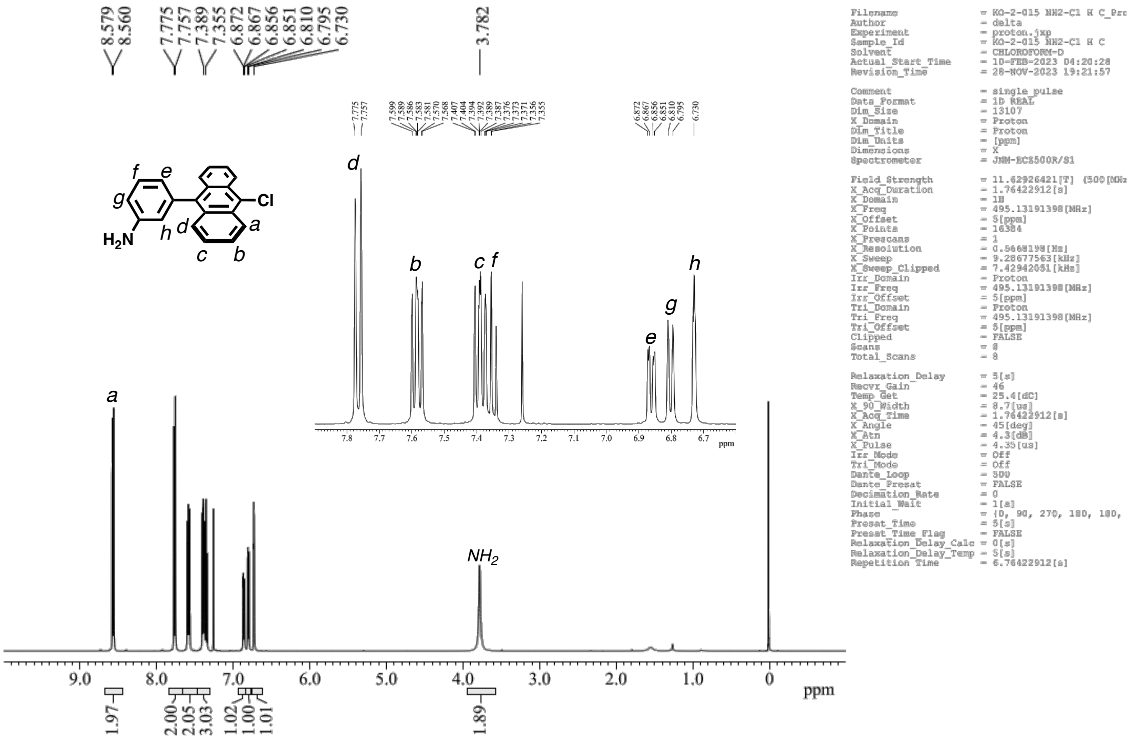

Figure S42. ^{1}H NMR spectrum (500 MHz, $CDCl_3$, 298 K) of **4b**.

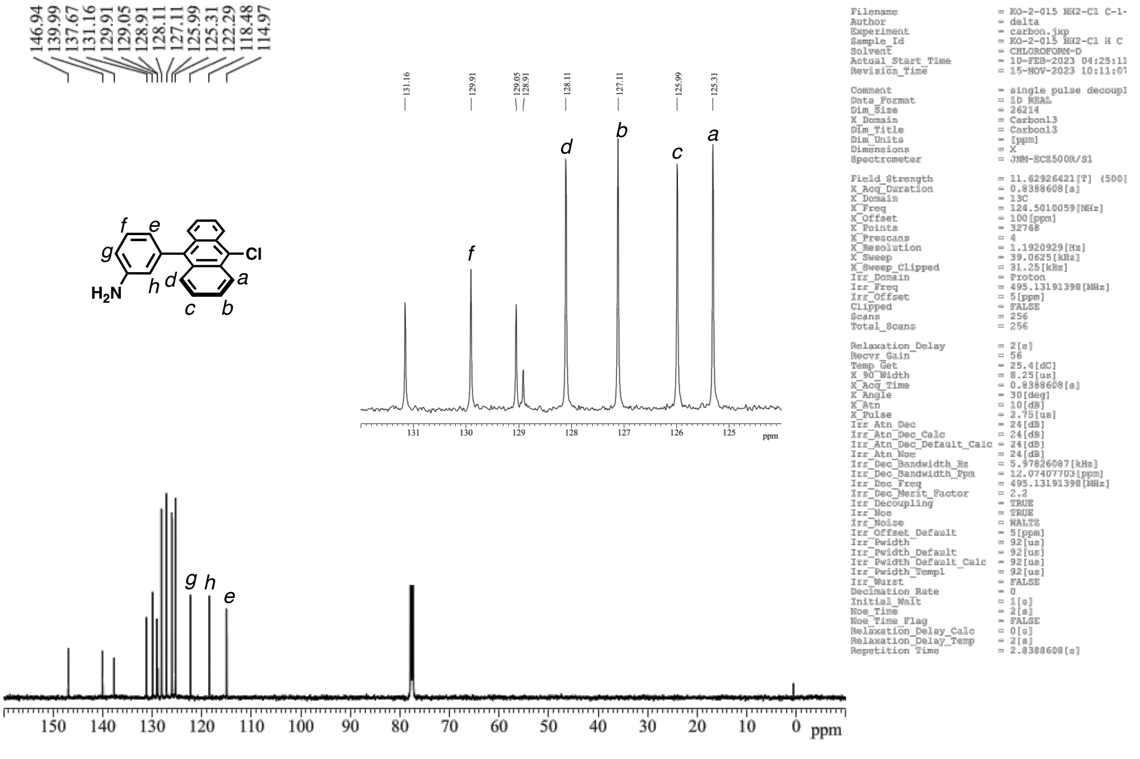

Figure S43. $^{13}C\{^{1}H\}$ NMR spectrum (125 MHz, $CDCl_3$, 298 K) of **4b**.

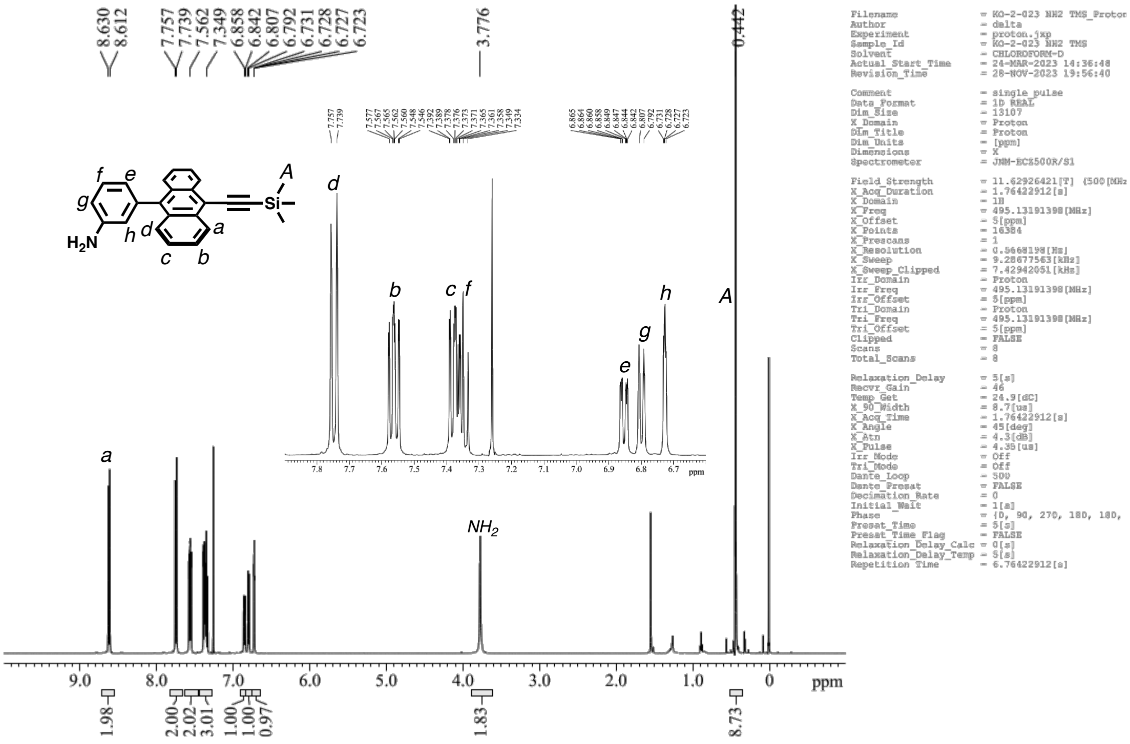

Figure S44. ^{1}H NMR spectrum (500 MHz, $CDCl_3$, 298 K) of **4c**.

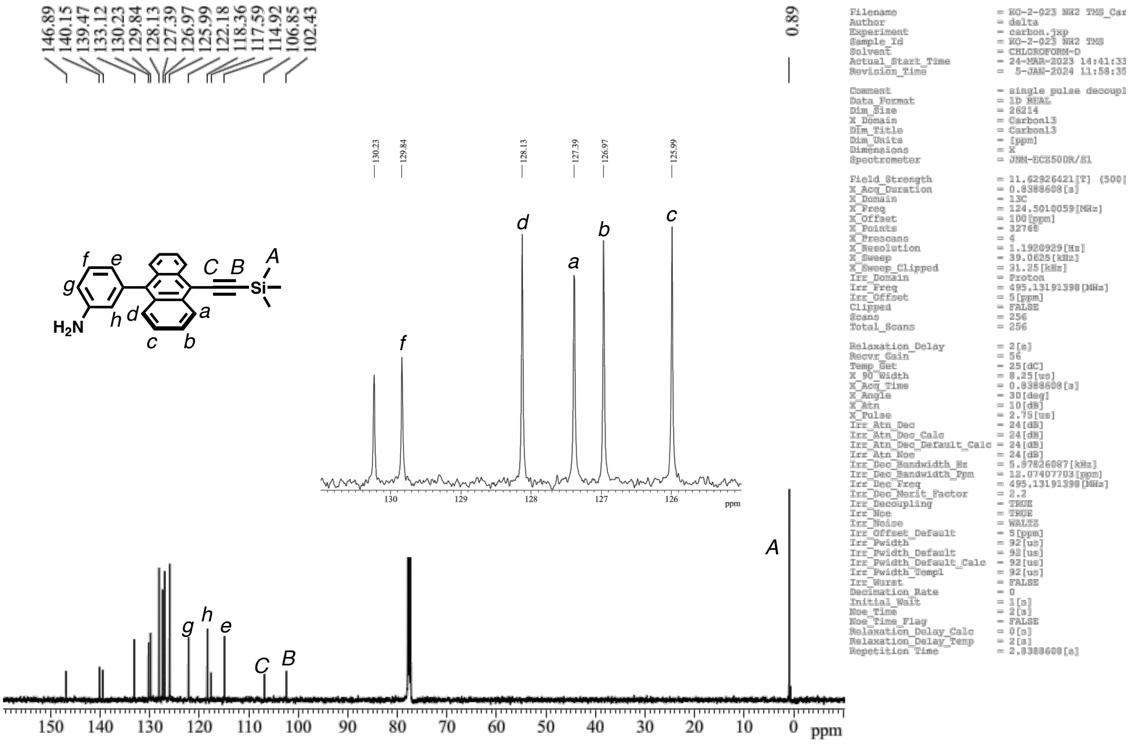

Figure S45. $^{13}C\{^{1}H\}$ NMR spectrum (125 MHz, $CDCl_3$, 298 K) of **4c**.

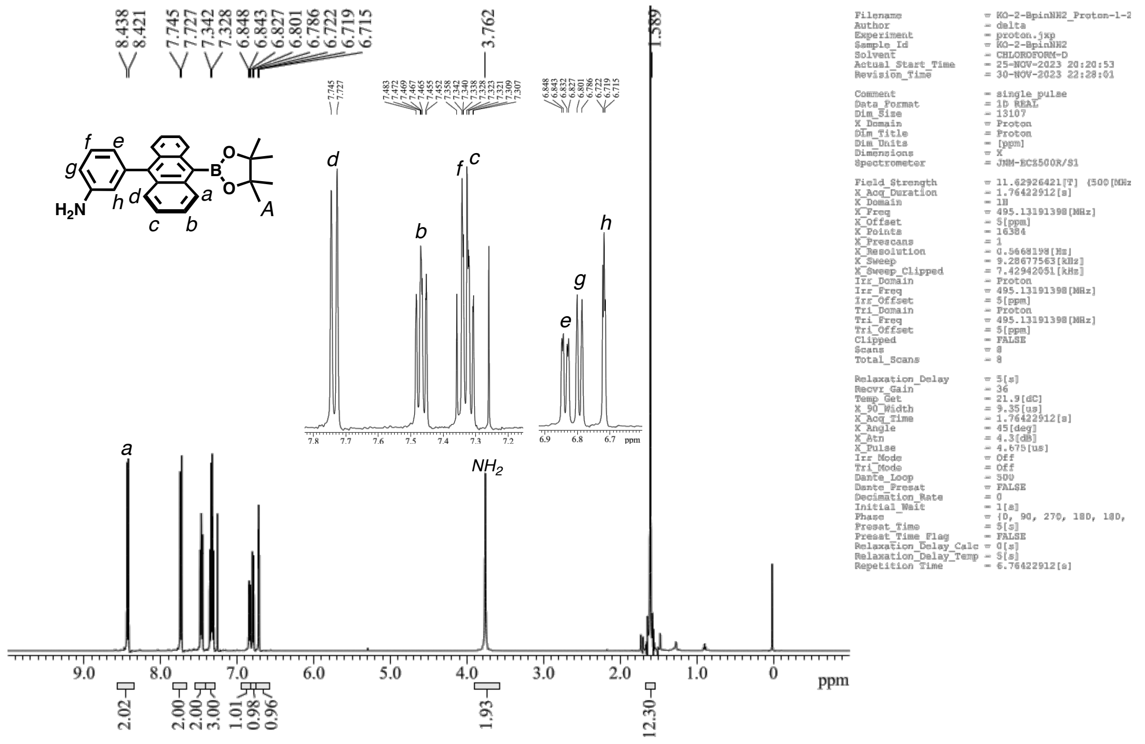

Figure S46. ^{1}H NMR spectrum (500 MHz, $CDCl_3$, 298 K) of **4d**.

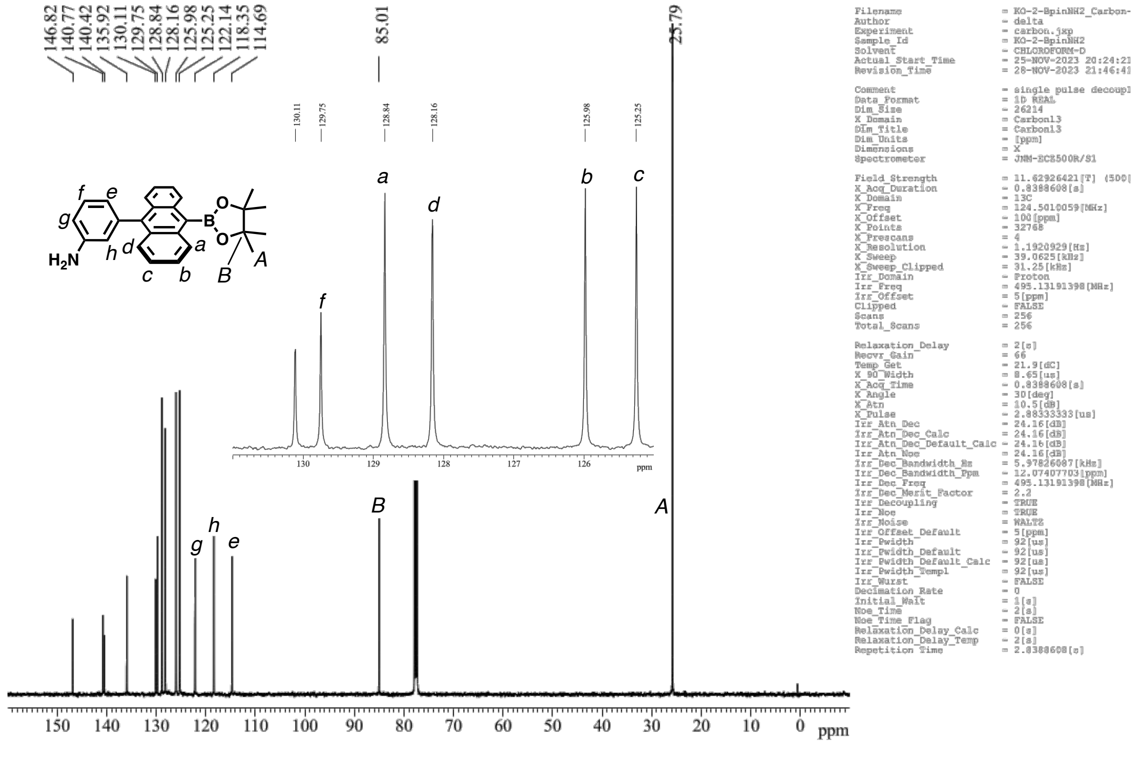

Figure S47. $^{13}C\{^{1}H\}$ NMR spectrum (125 MHz, $CDCl_3$, 298 K) of **4d**.

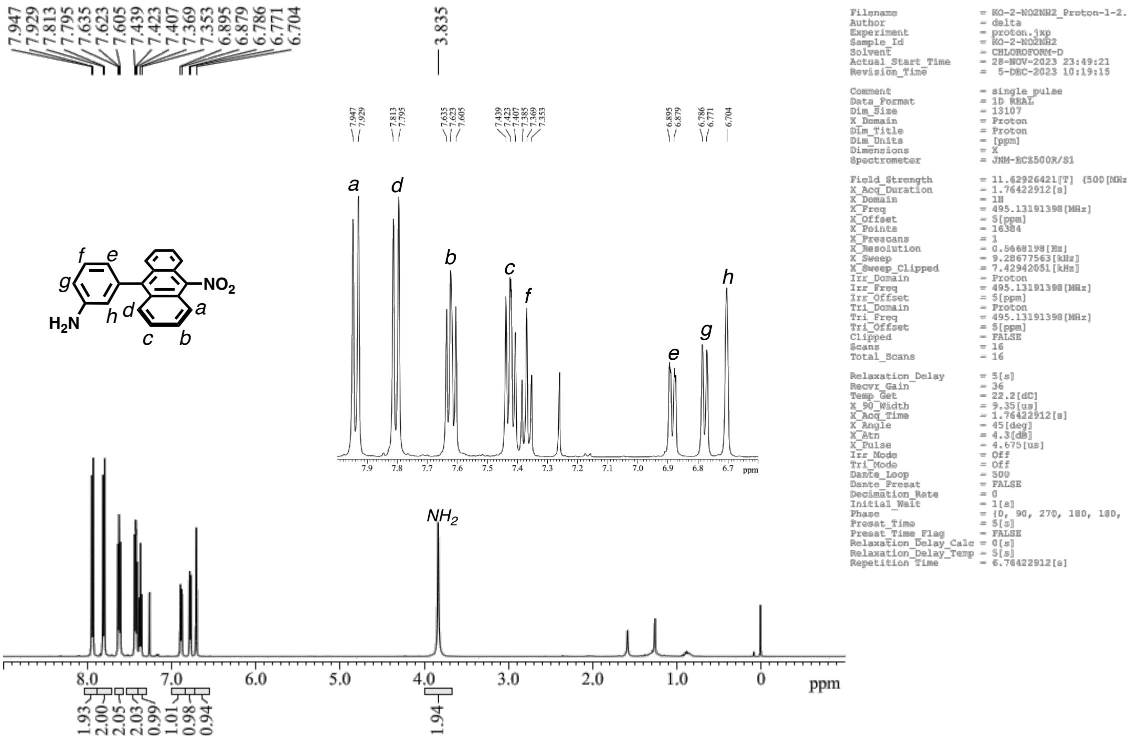

Figure S48. ^{1}H NMR spectrum (500 MHz, CDCl$_3$, 298 K) of **4e**.

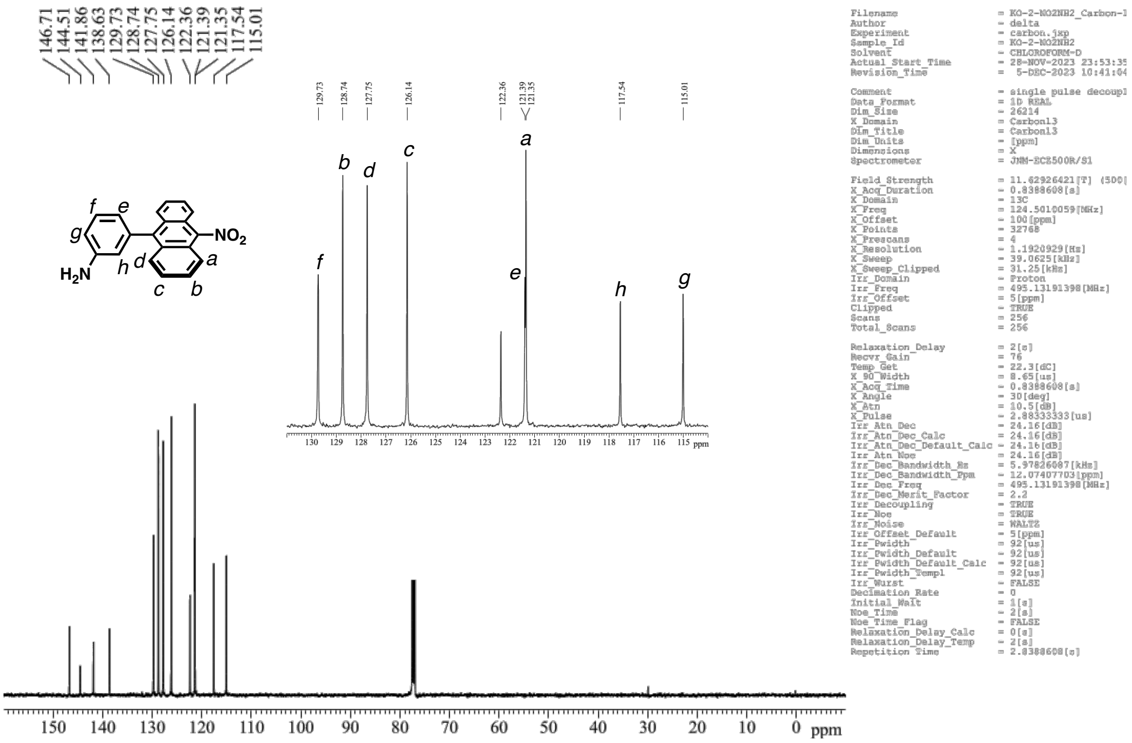

Figure S49. ^{13}C{^{1}H} NMR spectrum (125 MHz, CDCl$_3$, 298 K) of **4e**.

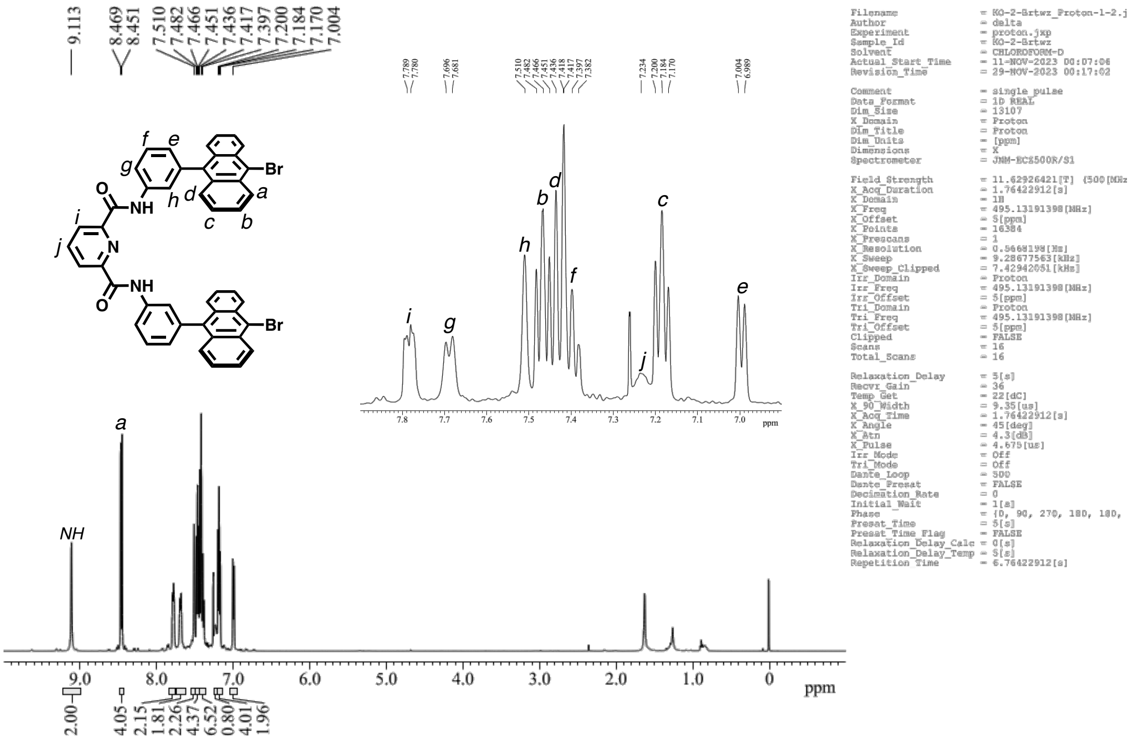

Figure S50. ^{1}H NMR spectrum (500 MHz, $CDCl_3$, 298 K) of **2a**.

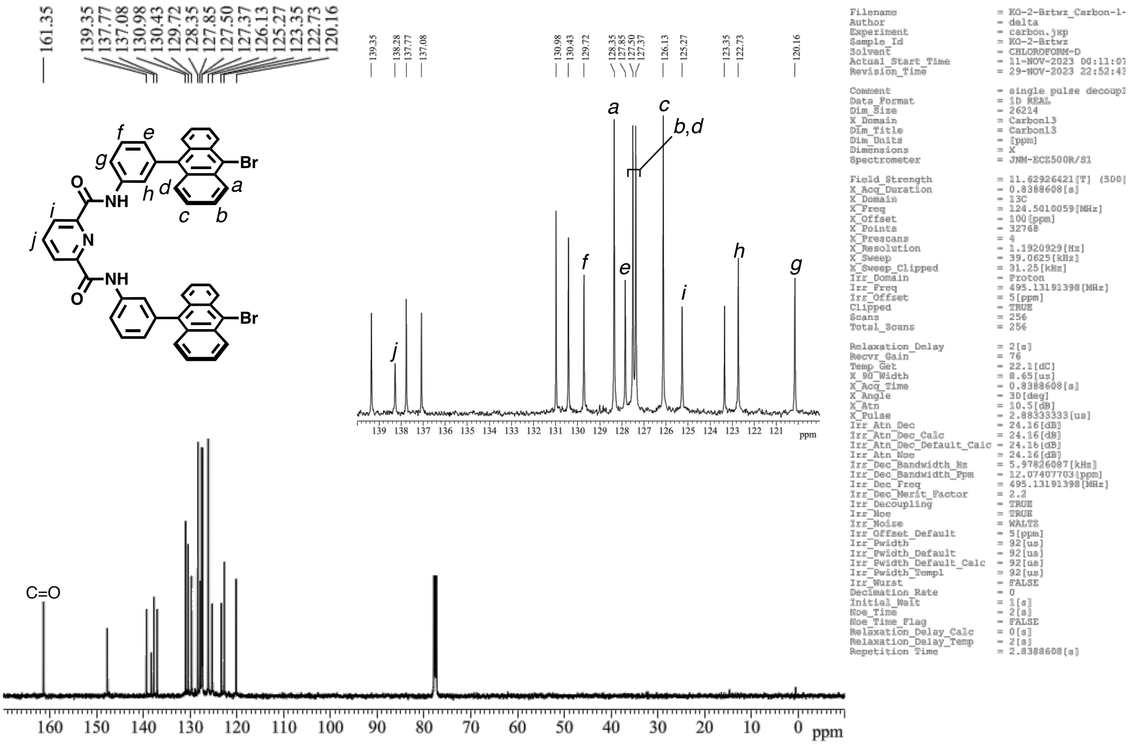

Figure S51. $^{13}C\{^{1}H\}$ NMR spectrum (125 MHz, $CDCl_3$, 298 K) of **2a**.

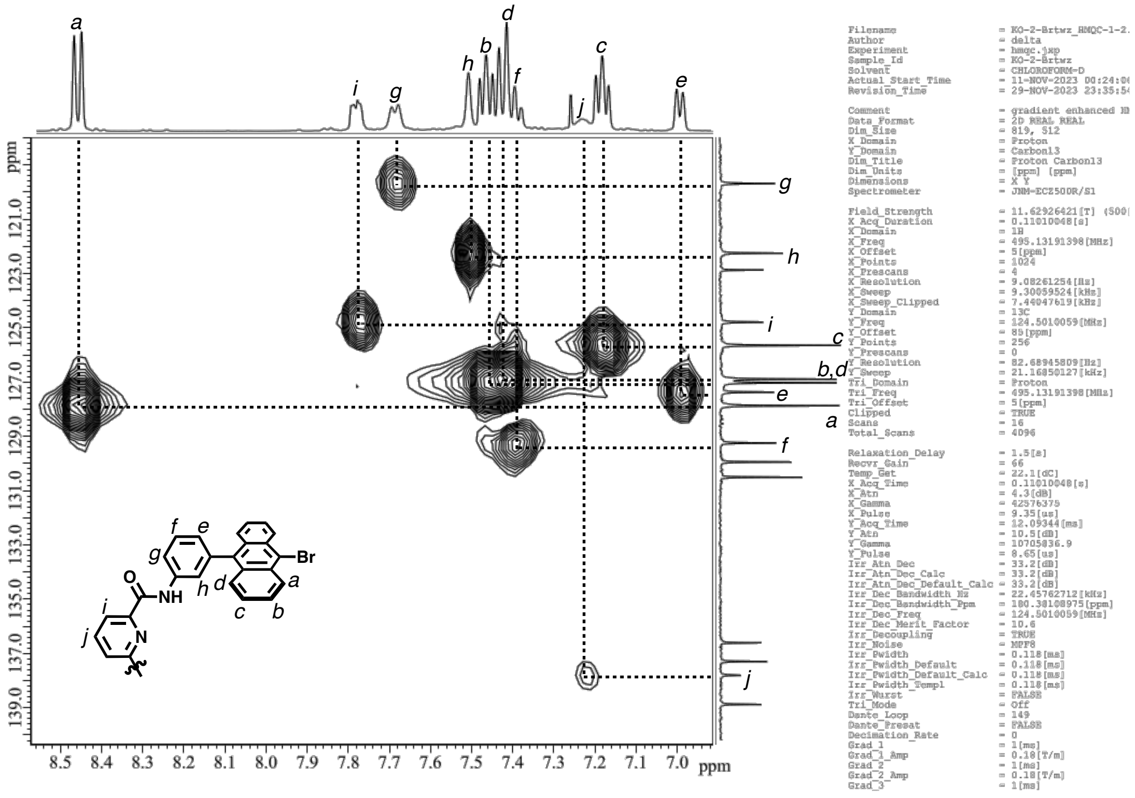

Figure S52. HMQC spectrum (500 MHz, $CDCl_3$, 298 K) of **2a**.

Analysis Info

Analysis Name	D:\Data\ofcbunseki\irai\2023\toyota_lab\yamashina\231019\KO-2-049-Br-000001.d	Acquisition Date	2023/10/19 11:07:54
Method	esi_posi_wide.m	Operator	BDAL@DE
Sample Name	KO-2-049-Br-	Instrument / Ser#	micrOTOF 213750.10321
Comment			

Acquisition Parameter

Source Type	ESI	Ion Polarity	Positive	Set Nebulizer	0.3 Bar
Focus	Not active			Set Dry Heater	200 ‹C
Scan Begin	50 m/z	Set Capillary	4500 V	Set Dry Gas	4.0 l/min
Scan End	2200 m/z	Set End Plate Offset	-500 V	Set Divert Valve	Waste

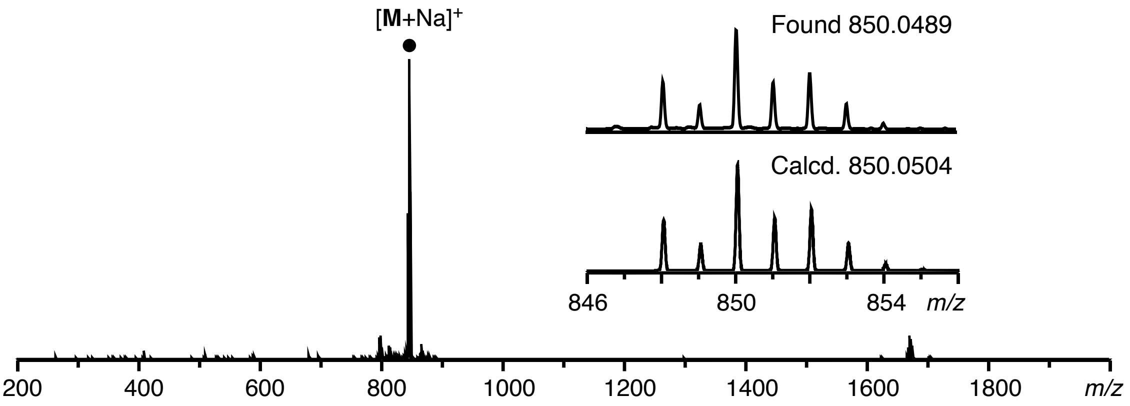

Figure S53. ESI-TOF MS spectrum ($CHCl_3$/MeOH) of **2a**.

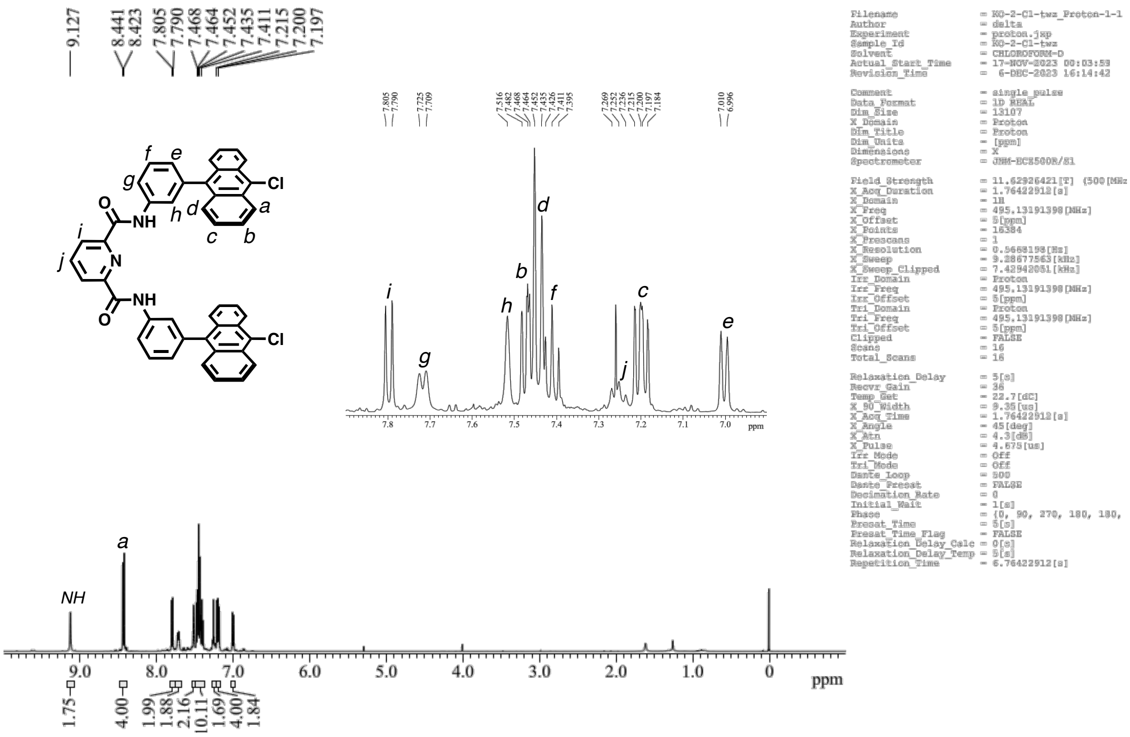

Figure S54. ^{1}H NMR spectrum (500 MHz, $CDCl_3$, 298 K) of **2b**.

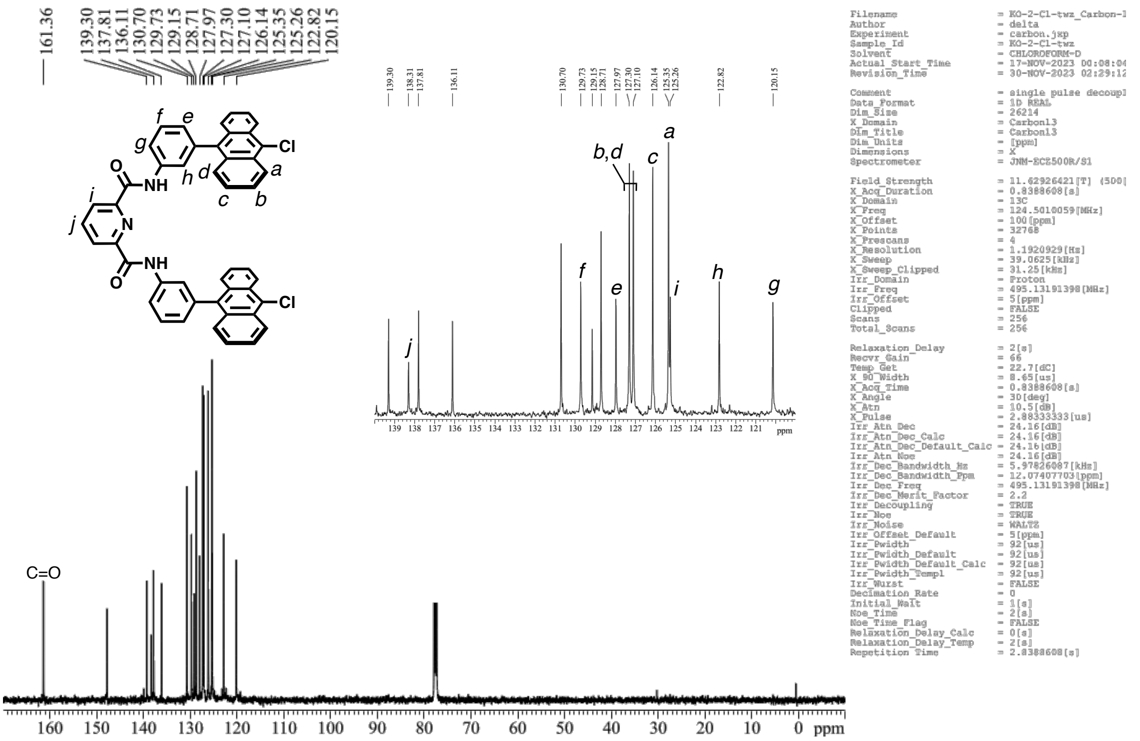

Figure S55. $^{13}C\{^{1}H\}$ NMR spectrum (125 MHz, $CDCl_3$, 298 K) of **2b**.

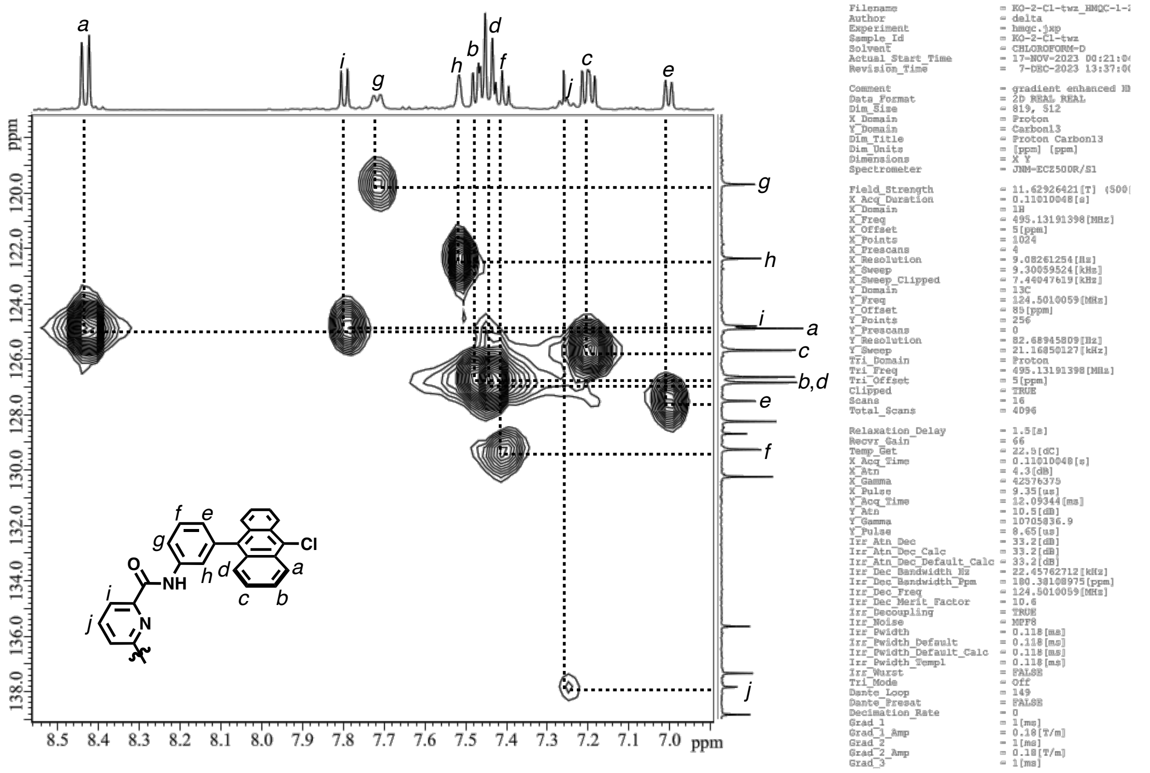

Figure S56. HMQC spectrum (500 MHz, $CDCl_3$, 298 K) of **2b**.

**Analysis Info**		Acquisition Date	2023/10/19 11:18:18	
Analysis Name	D:\Data\ofcbunseki\irai\2023\toyota_lab\yamashina\231019\KO-2-059-Cl-000001.d			
Method	esi_posi_wide.m	Operator	BDAL@DE	
Sample Name	KO-2-059-Cl-	Instrument / Ser#	micrOTOF	213750.10321
Comment				

**Acquisition Parameter**					
Source Type	ESI	Ion Polarity	Positive	Set Nebulizer	0.3 Bar
Focus	Not active			Set Dry Heater	200 ‹C
Scan Begin	50 m/z	Set Capillary	4500 V	Set Dry Gas	4.0 l/min
Scan End	2200 m/z	Set End Plate Offset	-500 V	Set Divert Valve	Waste

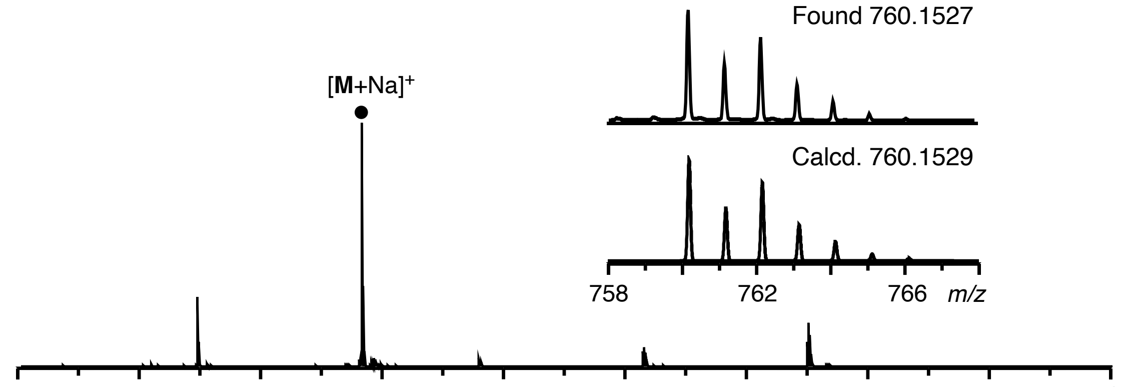

Figure S57. ESI-TOF MS spectrum ($CHCl_3$/MeOH) of **2b**.

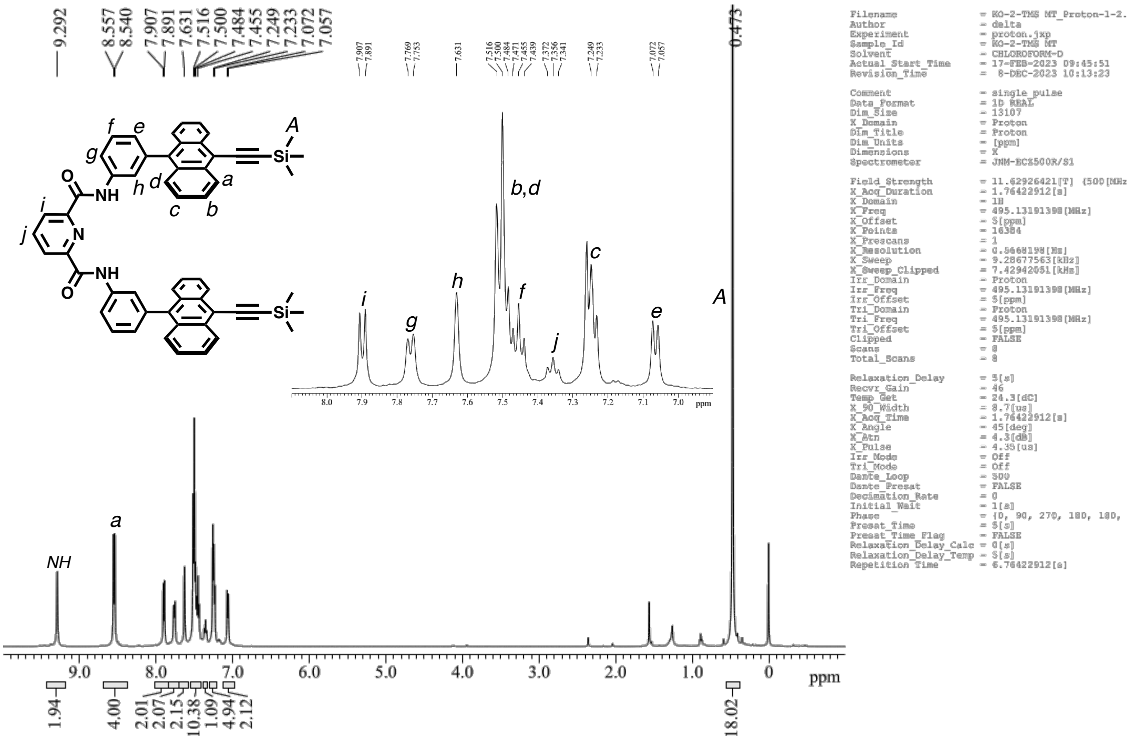

Figure S58. ^{1}H NMR spectrum (500 MHz, $CDCl_3$, 298 K) of **2c**.

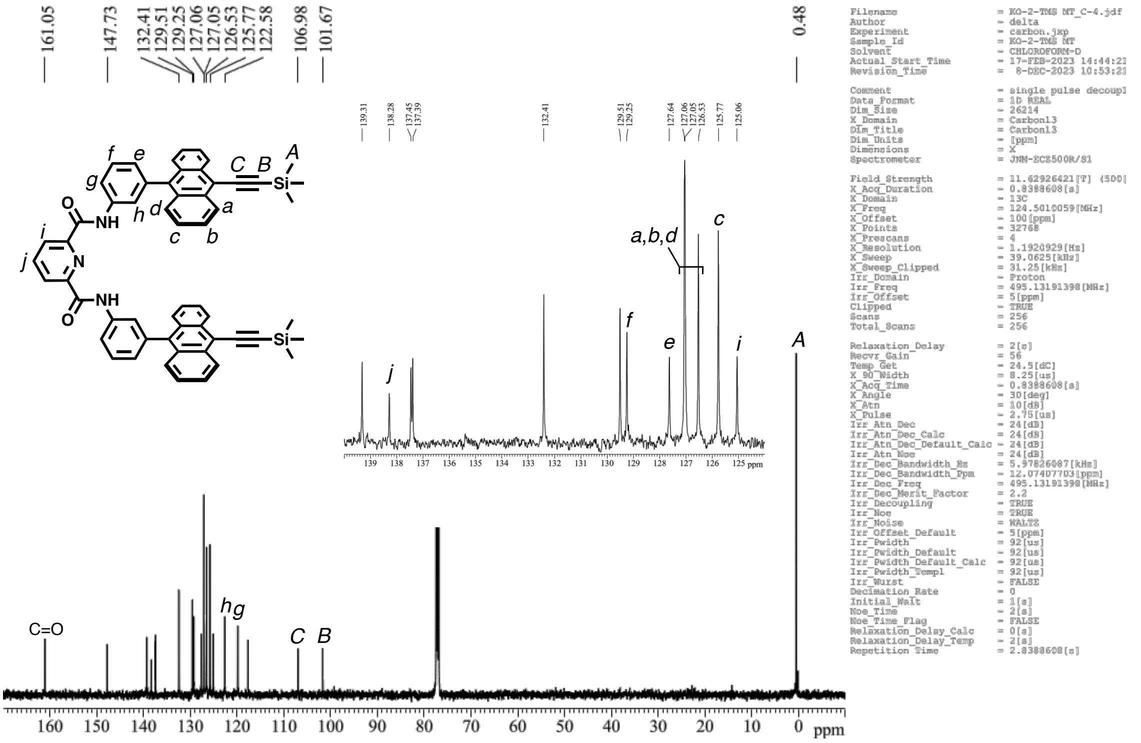

Figure S59. $^{13}C\{^1H\}$ NMR spectrum (125 MHz, $CDCl_3$, 298 K) of **2c**.

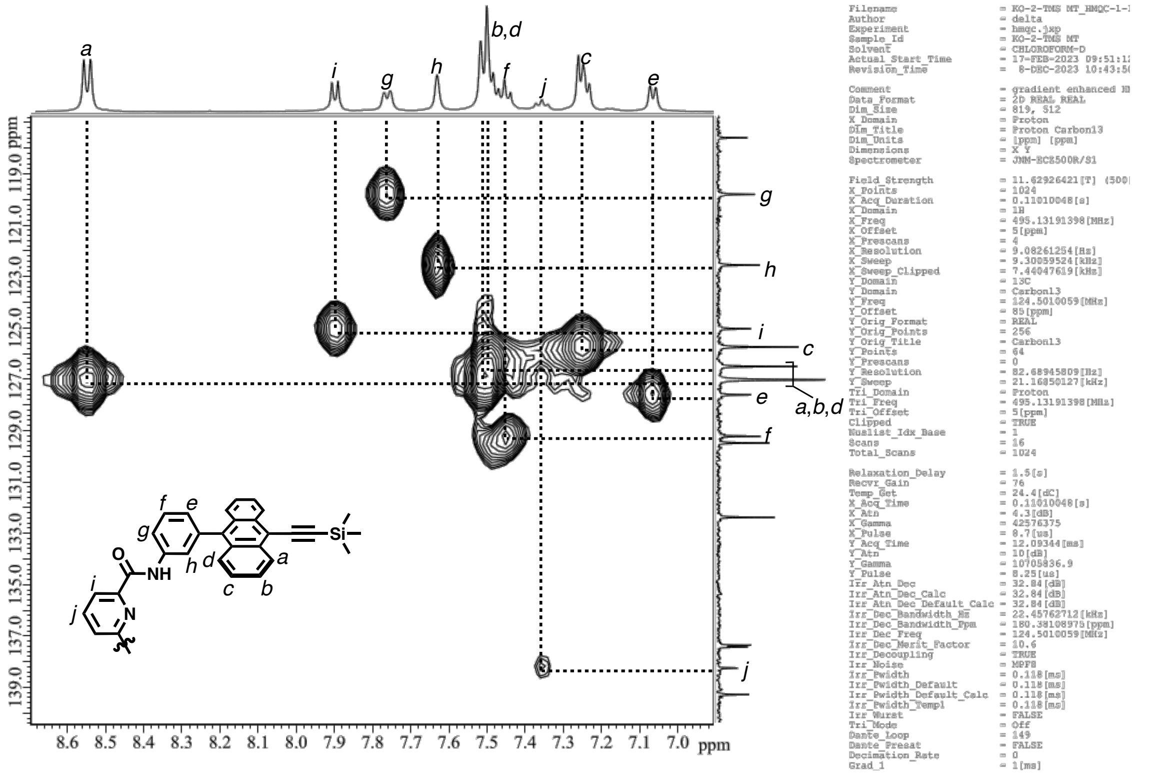

Figure S60. HMQC spectrum (500 MHz, $CDCl_3$, 298 K) of **2c**.

**Analysis Info**		Acquisition Date	2023/10/19 11:29:28	
Analysis Name	D:\Data\ofcbunseki\irai\2023\toyota_lab\yamashina\231019\KO-2-003-TMS-000001.d			
Method	esi_posi_wide.m	Operator	BDAL@DE	
Sample Name	KO-2-003-TMS-	Instrument / Ser#	micrOTOF	213750.10321
Comment				

Acquisition Parameter

Source Type	ESI	Ion Polarity	Positive	Set Nebulizer	0.3 Bar
Focus	Not active			Set Dry Heater	200 ‹C
Scan Begin	50 m/z	Set Capillary	4500 V	Set Dry Gas	4.0 l/min
Scan End	2200 m/z	Set End Plate Offset	-500 V	Set Divert Valve	Waste

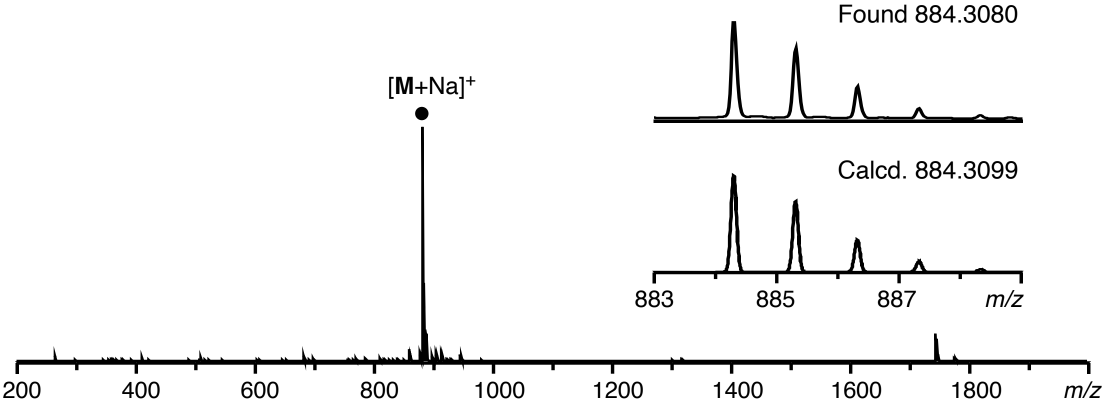

Figure S61. ESI-TOF MS spectrum ($CHCl_3$/MeOH) of **2c**.

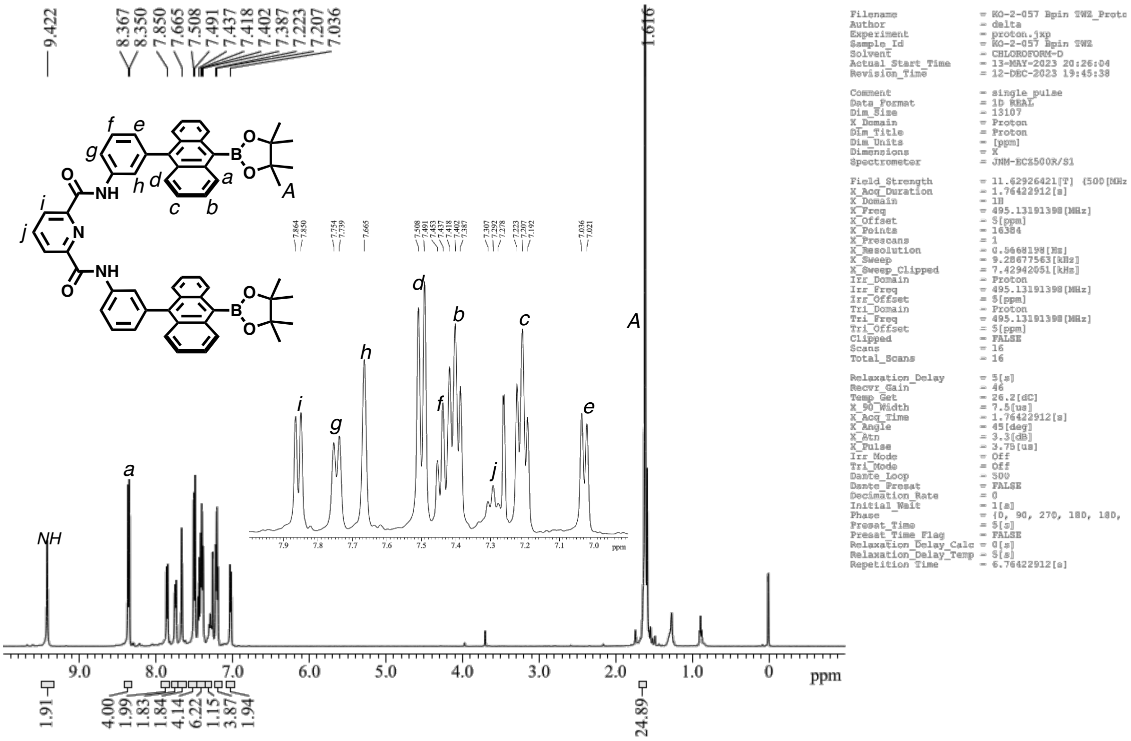

Figure S62. ^{1}H NMR spectrum (500 MHz, $CDCl_3$, 298 K) of **2d**.

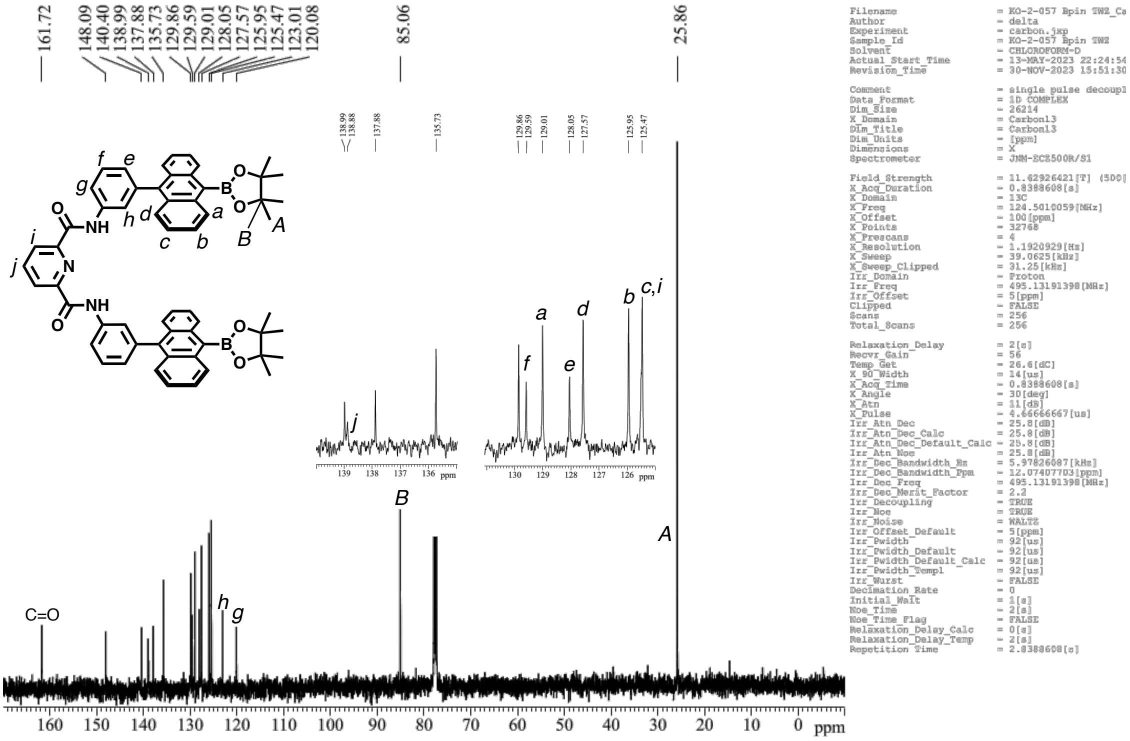

Figure S63. $^{13}C\{^{1}H\}$ NMR spectrum (125 MHz, $CDCl_3$, 298 K) of **2d**.

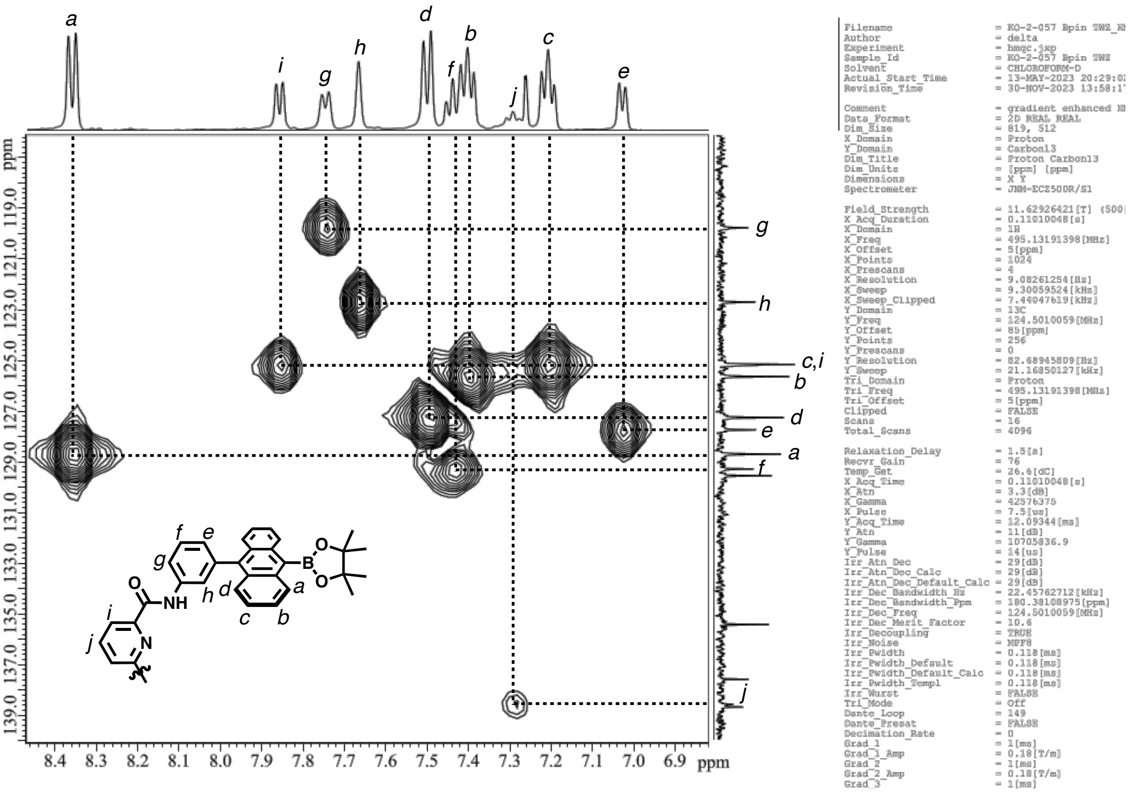

Figure S64. HMQC spectrum (500 MHz, $CDCl_3$, 298 K) of **2d**.

Analysis Info

Analysis Name	D:\Data\ofcbunseki\irai\2023\toyota_lab\yamashina\231019\KO-2-057-Bpin-000001.d	Acquisition Date	2023/10/19 11:39:42	
Method	esi_posi_wide.m	Operator	BDAL@DE	
Sample Name	KO-2-057-Bpin-	Instrument / Ser#	micrOTOF	213750.10321
Comment				

Acquisition Parameter

Source Type	ESI	Ion Polarity	Positive	Set Nebulizer	0.3 Bar
Focus	Not active			Set Dry Heater	200 ‹C
Scan Begin	50 m/z	Set Capillary	4500 V	Set Dry Gas	4.0 l/min
Scan End	2200 m/z	Set End Plate Offset	-500 V	Set Divert Valve	Waste

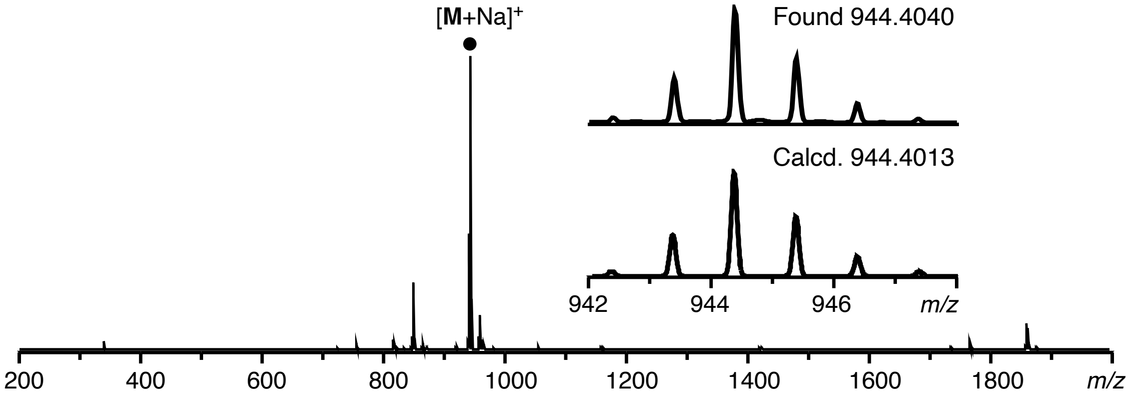

Figure S65. ESI-TOF MS spectrum ($CHCl_3$/MeOH) of **2d**.

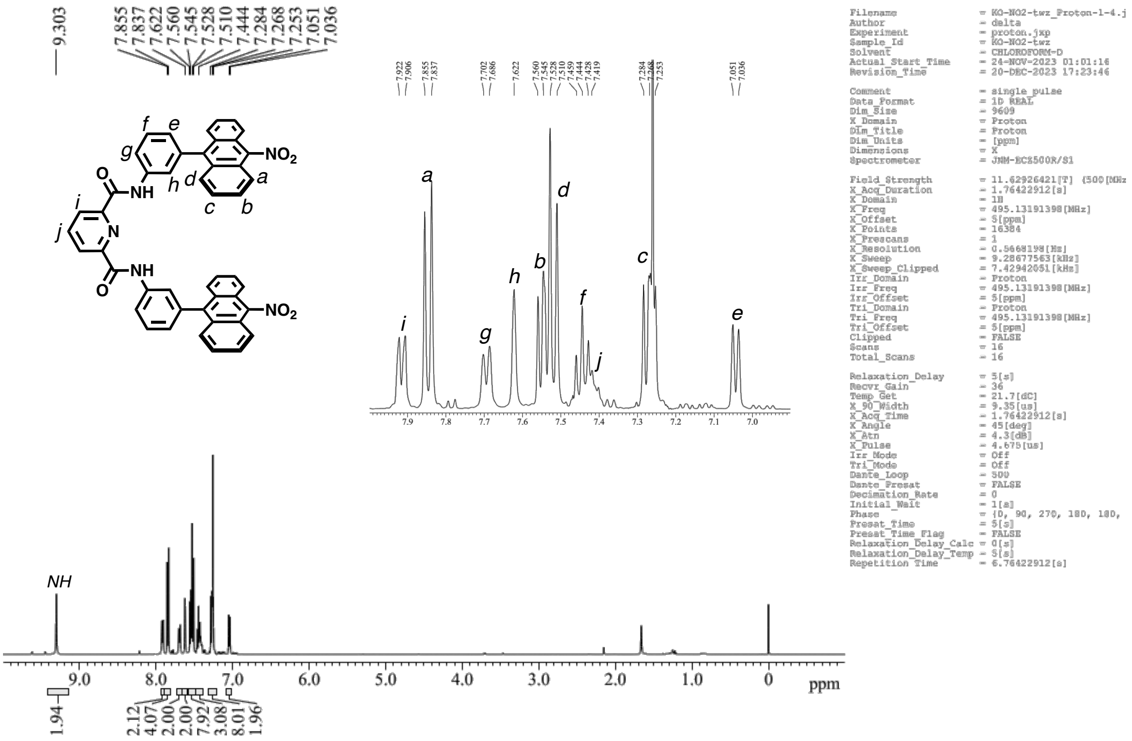

Figure S66. ^{1}H NMR spectrum (500 MHz, $CDCl_3$, 298 K) of **2e**.

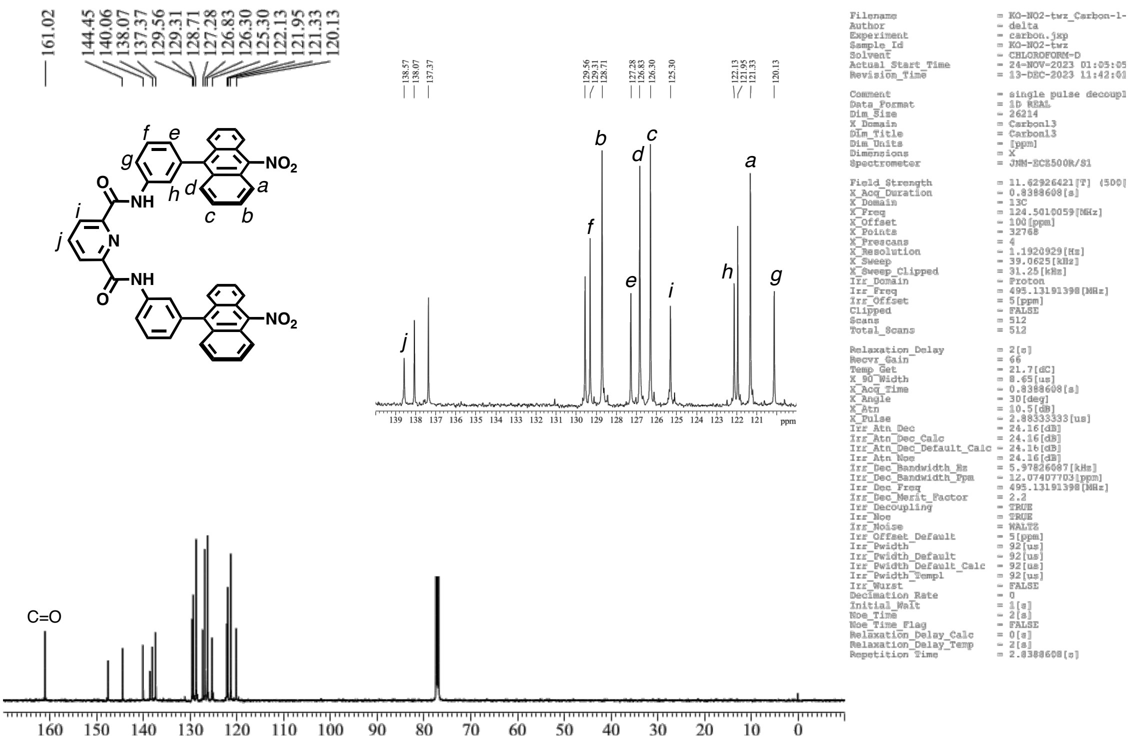

Figure S67. $^{13}C\{^1H\}$ NMR spectrum (125 MHz, $CDCl_3$, 298 K) of **2e**.

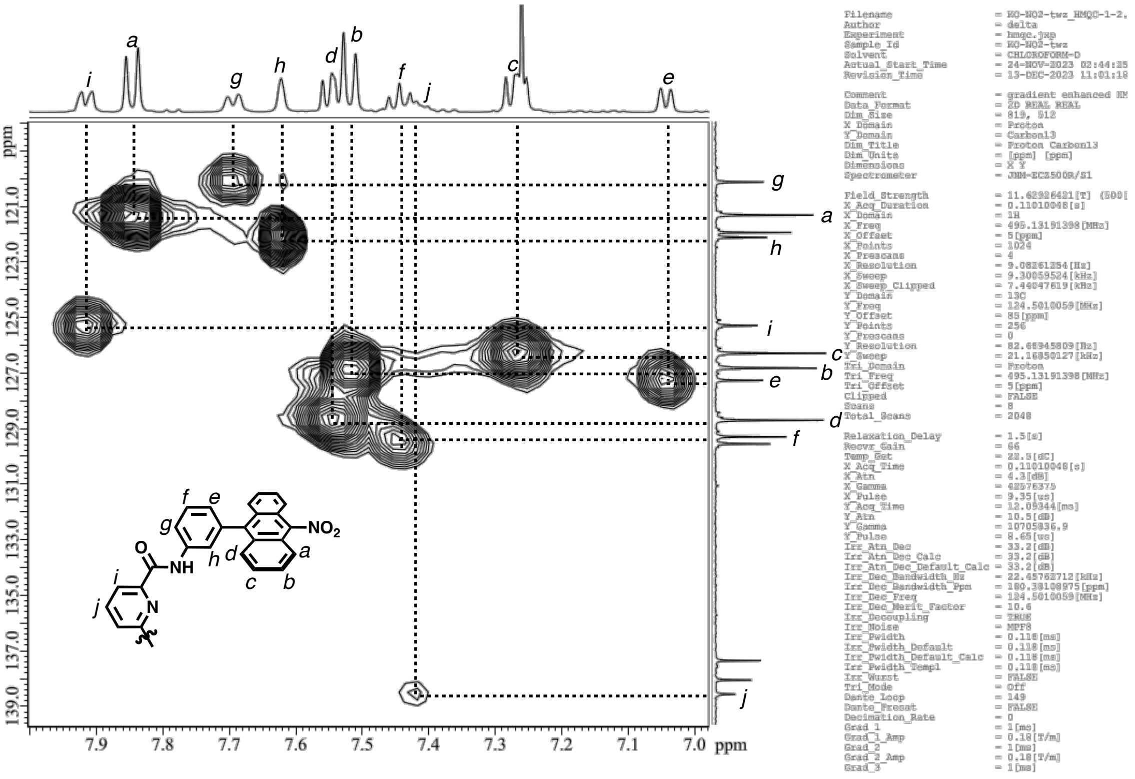

Figure S68. HMQC spectrum (500 MHz, $CDCl_3$, 298 K) of **2e**.

Analysis Info

Analysis Name	D:\Data\ofcbunseki\irai\2023\toyota_lab\yamashina\231211\KO-2-163-NO2-000001.d	Acquisition Date	2023/12/08 9:31:17	
Method	esi_posi_low.m	Operator	BDAL@DE	
Sample Name	KO-2-163-NO2-	Instrument / Ser#	micrOTOF	213750.10321
Comment				

Acquisition Parameter

Source Type	ESI	Ion Polarity	Positive	Set Nebulizer	0.3 Bar
Focus	Not active			Set Dry Heater	180 ‹C
Scan Begin	50 m/z	Set Capillary	4500 V	Set Dry Gas	4.0 l/min
Scan End	1800 m/z	Set End Plate Offset	-500 V	Set Divert Valve	Waste

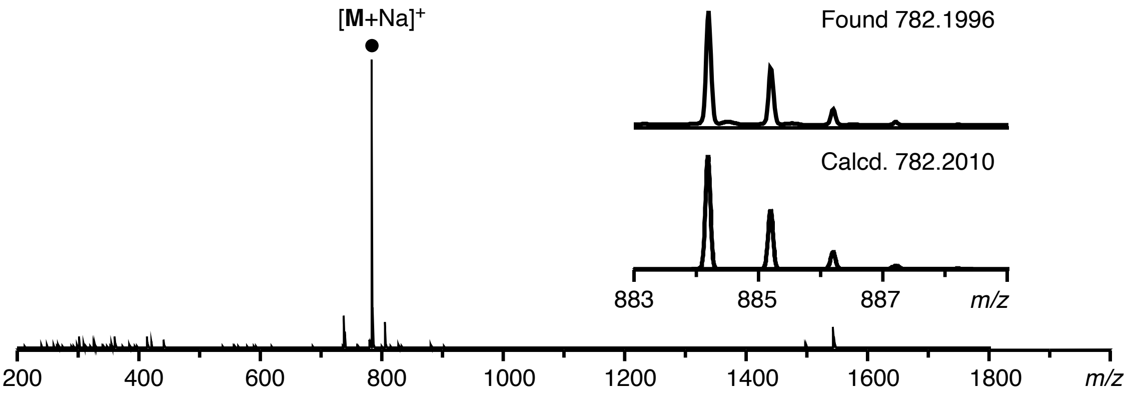

Figure S69. ESI-TOF MS spectrum (CH_3CN/MeOH) of **2e**.

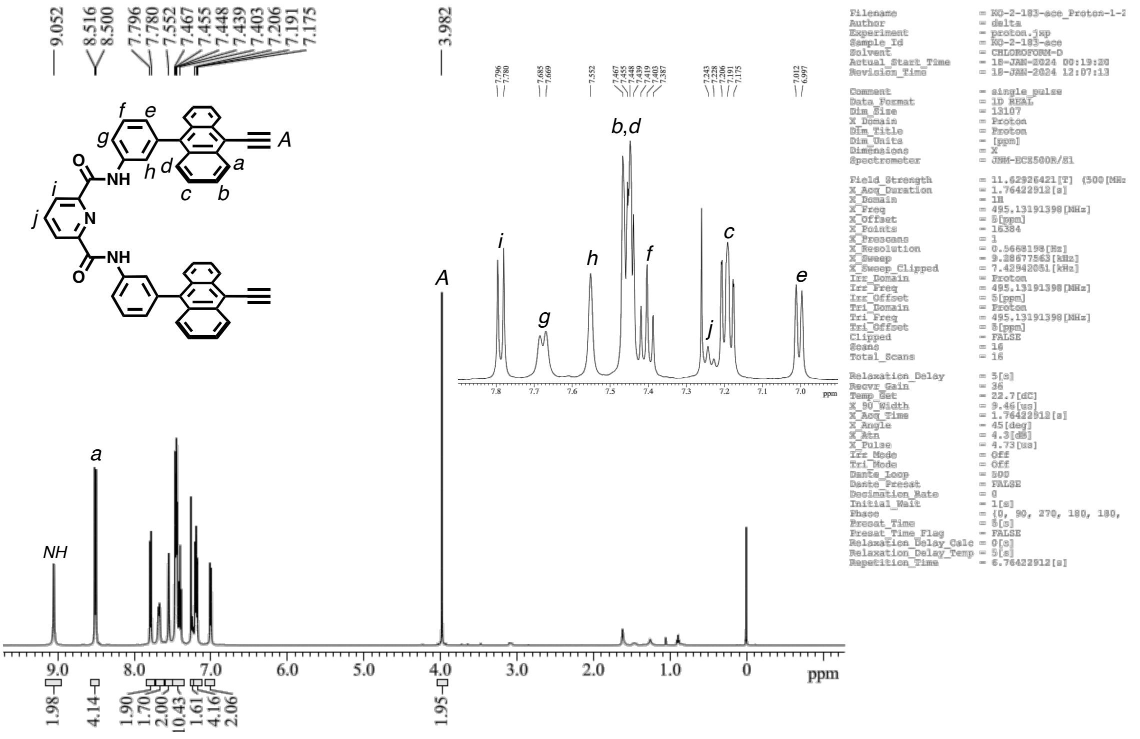

Figure S70. ^{1}H NMR spectrum (500 MHz, $CDCl_3$, 298 K) of **2f**.

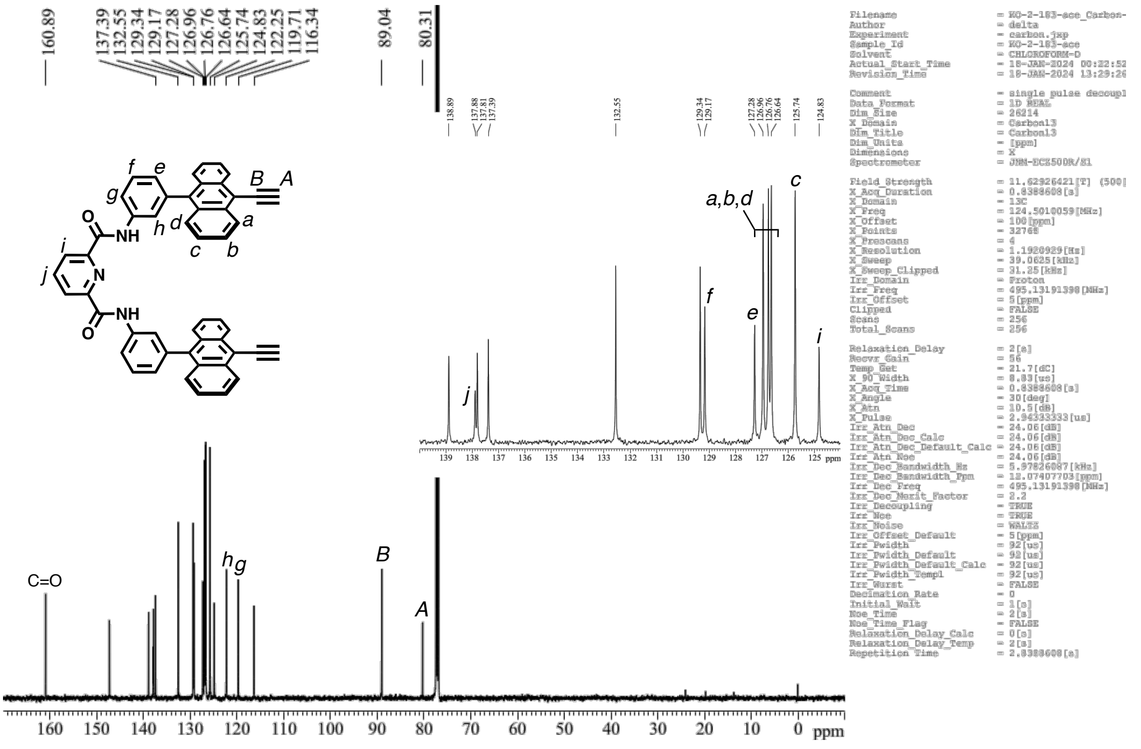

Figure S71. $^{13}C\{^{1}H\}$ NMR spectrum (125 MHz, $CDCl_3$, 298 K) of **2f**.

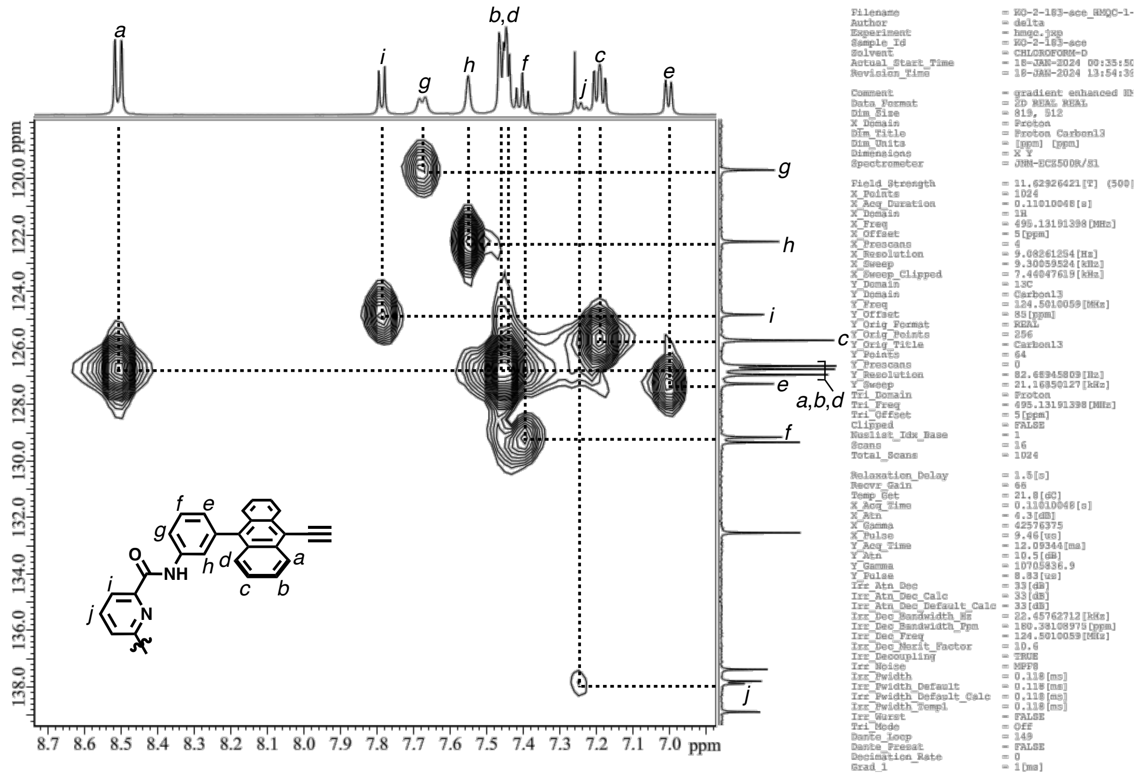

Figure S72. HMQC spectrum (500 MHz, $CDCl_3$, 298 K) of **2f**.

8. Cartesian Coordinates

Table S4. Cartesian Coordinates (B3LYP/6-31G (d,p) with the PCM ($CHCl_3$)) of head-to-head dimer (**2a**)$_2$.

head-to-head dimer (**2a**)$_2$											
C	-0.126	1.191	6.757	H	0.014	-1.346	2.92	C	-3.823	1.723	1.897
C	0	0	7.465	H	0.33	1.959	0.678	C	-3.246	-1.995	2.09
C	0.126	-1.191	6.757	H	-0.391	6.024	-0.532	C	-3.009	-2.966	3.025
C	0.123	-1.143	5.357	H	-0.956	6.614	1.817	C	-2.948	-2.629	4.405
N	0	0	4.673	H	-0.873	4.888	3.587	C	-3.123	-1.335	4.815
C	-0.123	1.143	5.357	H	0.873	-4.888	3.587	C	-3.896	3.475	3.638
C	-0.289	2.432	4.587	H	0.956	-6.614	1.817	C	-4.121	4.425	2.678
C	0.289	-2.432	4.587	H	0.391	-6.024	-0.532	C	-4.222	4.062	1.307
O	-0.461	3.496	5.184	H	-0.33	-1.959	0.678	C	-4.084	2.751	0.935
N	-0.224	2.293	3.228	Br	1.509	3.542	-6.111	C	3.682	-0.377	1.502
O	0.461	-3.496	5.184	H	2.459	4.182	0.102	C	3.823	-1.723	1.897
N	0.224	-2.293	3.228	H	4.824	4.408	-0.558	C	3.743	-2.092	3.295
C	-0.282	3.309	2.252	H	5.468	4.219	-2.965	C	3.54	-1.065	4.238
C	0.282	-3.309	2.252	H	3.764	3.814	-4.676	C	3.359	0.281	3.874
C	0.03	2.976	0.921	H	-1.087	3.061	-5.867	C	3.426	0.623	2.466
C	0	3.954	-0.08	H	-3.403	2.742	-5.138	C	4.084	-2.751	0.935
C	-0.366	5.27	0.248	H	-3.994	2.852	-2.716	C	4.222	-4.062	1.307
C	-0.676	5.596	1.563	H	-2.25	3.299	-1.04	C	4.121	-4.425	2.678
C	-0.632	4.634	2.569	Br	-1.509	-3.542	-6.111	C	3.896	-3.475	3.638
C	0.632	-4.634	2.569	H	2.25	-3.299	-1.04	C	3.123	1.335	4.815
C	0.676	-5.596	1.563	H	3.994	-2.852	-2.716	C	2.948	2.629	4.405
C	0.366	-5.27	0.248	H	3.403	-2.742	-5.138	C	3.009	2.966	3.025
C	0	-3.954	-0.08	H	1.087	-3.061	-5.867	C	3.246	1.995	2.09
C	-0.03	-2.976	0.921	H	-3.764	-3.814	-4.676	H	-2.148	-0.238	-7.245
C	0.377	3.682	-1.502	H	-5.468	-4.219	-2.965	H	0	0	-8.549
C	1.723	3.823	-1.897	H	-4.824	-4.408	-0.558	H	2.148	0.238	-7.245
C	2.092	3.743	-3.295	H	-2.459	-4.182	0.102	H	-1.346	-0.014	-2.92
C	1.065	3.54	-4.238	C	-1.191	-0.126	-6.757	H	1.346	0.014	-2.92
C	-0.281	3.359	-3.874	C	0	0	-7.465	H	-1.959	0.33	-0.678
C	-0.623	3.426	-2.466	C	1.191	0.126	-6.757	H	-6.024	-0.391	0.532
C	2.751	4.084	-0.935	C	1.143	0.123	-5.357	H	-6.614	-0.956	-1.817
C	4.062	4.222	-1.307	N	0	0	-4.673	H	-4.888	-0.873	-3.587
C	4.425	4.121	-2.678	C	-1.143	-0.123	-5.357	H	4.888	0.873	-3.587
C	3.475	3.896	-3.638	C	-2.432	-0.289	-4.587	H	6.614	0.956	-1.817
C	-1.335	3.123	-4.815	C	2.432	0.289	-4.587	H	6.024	0.391	0.532
C	-2.629	2.948	-4.405	O	-3.496	-0.461	-5.184	H	1.959	-0.33	-0.678
C	-2.966	3.009	-3.025	N	-2.293	-0.224	-3.228	Br	-3.542	1.509	6.111
C	-1.995	3.246	-2.09	O	3.496	0.461	-5.184	H	-3.299	-2.25	1.04
C	-0.377	-3.682	-1.502	N	2.293	0.224	-3.228	H	-2.852	-3.994	2.716
C	0.623	-3.426	-2.466	C	-3.309	-0.282	-2.252	H	-2.742	-3.403	5.138
C	0.281	-3.359	-3.874	C	3.309	0.282	-2.252	H	-3.061	-1.087	5.867
C	-1.065	-3.54	-4.238	C	-2.976	0.03	-0.921	H	-3.814	3.764	4.676
C	-2.092	-3.743	-3.295	C	-3.954	0	0.08	H	-4.219	5.468	2.965
C	-1.723	-3.823	-1.897	C	-5.27	-0.366	-0.248	H	-4.408	4.824	0.558
C	1.995	-3.246	-2.09	C	-5.596	-0.676	-1.563	H	-4.182	2.459	-0.102
C	2.966	-3.009	-3.025	C	-4.634	-0.632	-2.569	Br	3.542	-1.509	6.111
C	2.629	-2.948	-4.405	C	4.634	0.632	-2.569	H	4.182	-2.459	-0.102
C	1.335	-3.123	-4.815	C	5.596	0.676	-1.563	H	4.408	-4.824	0.558
C	-3.475	-3.896	-3.638	C	5.27	0.366	-0.248	H	4.219	-5.468	2.965
C	-4.425	-4.121	-2.678	C	3.954	0	0.08	H	3.814	-3.764	4.676
C	-4.062	-4.222	-1.307	C	2.976	-0.03	-0.921	H	3.061	1.087	5.867
C	-2.751	-4.084	-0.935	C	-3.682	0.377	1.502	H	2.742	3.403	5.138
H	-0.238	2.148	7.245	C	-3.426	-0.623	2.466	H	2.852	3.994	2.716
H	0	0	8.549	C	-3.359	-0.281	3.874	H	3.299	2.25	1.04
H	0.238	-2.148	7.245	C	-3.54	1.065	4.238				
H	-0.014	1.346	2.92	C	-3.743	2.092	3.295				

Table S5. Cartesian Coordinates (B3LYP/6-31G (d,p) with the PCM ($CHCl_3$)) of head-to-tail dimer (**2a**)$_2$.

head-to-tail dimer (**2a**)$_2$											
C	6.881	-4.231	0.595	H	7.521	-4.745	2.558	C	1.482	4.013	-1.542
C	5.278	-2.61	-3.864	H	4.602	-1.751	-5.691	C	0.145	4.523	-1.321
C	6.626	-3.197	-0.36	H	5.15	0.592	-5.008	C	-0.01	5.619	-0.411
C	6.883	-1.826	0.031	H	6.125	1.031	-2.795	C	1.074	6.239	0.152
C	7.356	-1.574	1.359	Br	5.589	-5.227	-2.109	C	2.391	5.791	-0.143
C	7.577	-2.595	2.243	H	4.555	0.486	0.071	C	-0.316	0.824	-4.802
C	7.341	-3.941	1.852	H	6.412	4.166	1.271	C	-1.61	1.389	-4.64
C	5.033	-1.562	-4.713	H	8.568	3.341	0.351	C	-1.814	2.391	-3.728
C	5.343	-0.229	-4.326	H	8.735	1.076	-0.658	C	-0.746	2.9	-2.921
C	5.884	0.017	-3.092	H	3.312	2.107	0.964	C	0.573	2.336	-3.107
C	6.135	-1.041	-2.162	H	-1.555	3.604	2.906	C	1.624	2.868	-2.344
C	5.84	-2.399	-2.565	H	-0.162	5.302	4.15	C	-0.955	3.926	-1.974
C	6.093	-3.427	-1.641	H	2.324	5.357	3.708	C	-2.349	4.364	-1.671
C	6.619	-0.772	-0.867	H	-7.952	-0.912	-1.916	C	-3.208	3.48	-1.01
C	6.642	0.634	-0.364	H	-6.396	-5.238	0.099	C	-4.536	3.837	-0.735
C	5.432	1.121	0.121	H	-3.225	0.454	-2.574	C	-5.004	5.106	-1.115
C	5.337	2.383	0.719	H	-5.043	1.955	-3.232	C	-4.141	5.982	-1.774
C	6.477	3.193	0.802	H	-7.422	1.267	-2.903	C	-2.824	5.624	-2.059
C	7.685	2.713	0.289	H	-4.562	-6.728	0.747	N	-5.341	2.879	-0.089
C	7.784	1.443	-0.286	H	-2.186	-6.024	0.4	C	-6.702	2.854	-0.016
N	4.074	2.729	1.21	H	-1.685	-3.847	-0.598	O	-7.446	3.756	-0.402
C	3.693	3.756	2.014	Br	-7.971	-3.283	-0.736	C	-7.284	1.589	0.566
O	4.43	4.634	2.462	H	-2.429	0.053	-0.053	H	4.362	-1.981	1.096
C	2.213	3.733	2.314	H	1.63	-0.221	-1.426	H	3.091	1.489	4.505
N	1.47	2.83	1.664	H	1	-2.106	-2.883	H	-0.352	-3.535	1
C	-0.483	3.677	2.771	H	-1.348	-2.926	-2.944	H	1.31	-4.542	-0.496
C	0.296	4.61	3.451	H	1.029	1.36	0.153	H	3.681	-3.777	-0.432
C	1.669	4.647	3.218	C	3.331	-2.31	1.077	H	1.384	2.546	5.908
C	-6.919	-0.621	-2.058	C	2.078	1.106	4.505	H	-0.954	1.659	5.935
C	-5.37	-4.936	-0.068	C	2.388	-1.686	1.958	H	-1.564	-0.242	4.517
C	-5.89	-1.523	-1.639	C	1.018	-2.153	1.944	Br	4.571	-0.151	3.027
C	-4.509	-1.122	-1.816	C	0.675	-3.2	1.03	H	-2.322	-0.458	2.098
C	-4.251	0.154	-2.413	C	1.604	-3.76	0.197	H	-4.837	-3.841	2.939
C	-5.268	0.988	-2.794	C	2.953	-3.313	0.223	H	-2.857	-5.09	3.748
C	-6.621	0.595	-2.61	C	1.124	1.694	5.29	H	-0.602	-4.05	3.692
C	-4.344	-5.765	0.295	C	-0.208	1.195	5.297	H	-4.569	-0.363	1.649
C	-2.995	-5.367	0.096	C	-0.55	0.135	4.502	H	-10.31	0.141	1.053
C	-2.713	-4.149	-0.46	C	0.403	-0.494	3.635	H	-8.756	-1.67	1.881
C	-3.749	-3.248	-0.868	C	1.766	-0.004	3.657	H	3.588	4.338	-1.137
C	-5.122	-3.66	-0.666	C	2.719	-0.649	2.849	H	1.722	0.848	-4.187
C	-6.144	-2.774	-1.051	C	0.054	-1.585	2.809	H	-1.01	5.966	-0.185
C	-3.458	-1.978	-1.418	C	-1.312	-2.186	2.873	H	0.93	7.074	0.83
C	-2.043	-1.5	-1.483	C	-2.438	-1.478	2.447	H	3.244	6.291	0.303
C	-1.681	-0.428	-0.66	C	-3.711	-2.068	2.469	H	-0.164	0.025	-5.522
C	-0.358	0.026	-0.618	C	-3.861	-3.38	2.939	H	-2.438	1.026	-5.239
C	0.611	-0.593	-1.42	C	-2.737	-4.074	3.384	H	-2.797	2.83	-3.618
C	0.245	-1.644	-2.254	C	-1.472	-3.495	3.355	Br	3.316	1.942	-2.339
C	-1.072	-2.103	-2.294	N	-4.786	-1.288	2.002	H	-2.84	2.507	-0.706
N	0.054	1.086	0.21	C	-6.114	-1.597	2.001	H	-6.025	5.386	-0.907
C	-0.664	1.792	1.123	O	-6.6	-2.657	2.393	H	-4.51	6.959	-2.072
O	-1.873	1.661	1.348	C	-7.009	-0.497	1.484	H	-2.165	6.309	-2.582
C	0.151	2.807	1.881	N	-6.46	0.635	1.022	H	-4.884	2.026	0.216
H	6.694	-5.259	0.31	C	-9.236	0.279	1.045	C	-8.676	1.459	0.567
H	5.041	-3.621	-4.17	C	-8.39	-0.723	1.51	H	-9.278	2.274	0.186
H	7.528	-0.547	1.658	C	2.585	4.697	-0.942				
H	7.93	-2.379	3.247	C	0.737	1.28	-4.058				

Table S6. Cartesian Coordinates (PM6) of head-to-head dimer (**2c**)$_2$.

head-to-head dimer (**2c**)$_2$											
C	6.146	-0.911	0.786	C	-6.09	3.69	-1.184	C	6.089	-3.691	-1.184
C	6.851	0.002	-0.001	H	-0.564	5.705	-0.985	H	1.197	-1.619	-3.726
C	6.145	0.914	-0.788	H	-1.504	7.434	0.546	H	2.711	0.117	-4.672
C	4.737	0.873	-0.766	H	-3.897	7.401	1.153	H	5.123	0.09	-4.164
N	4.033	0.001	-0.001	H	-5.413	5.659	0.221	H	6.087	-1.689	-2.704
C	4.738	-0.871	0.765	H	-6.087	1.688	-2.704	H	5.412	-5.66	0.221
C	3.992	-1.81	1.692	H	-5.122	-0.09	-4.164	H	3.895	-7.402	1.151
C	3.991	1.812	-1.693	H	-2.71	-0.116	-4.673	H	1.502	-7.435	0.543
O	4.533	-2.229	2.701	H	-1.197	1.62	-3.725	H	0.563	-5.705	-0.988
N	2.69	-2.152	1.313	C	-6.145	-0.914	-0.787	C	6.087	3.692	1.184
O	4.532	2.232	-2.703	C	-6.851	-0.002	0	H	0.562	5.706	0.987
N	2.689	2.153	-1.314	C	-6.146	0.911	0.787	H	1.501	7.435	-0.545
C	1.786	-2.94	2.097	C	-4.738	0.871	0.765	H	3.894	7.403	-1.152
C	1.784	2.941	-2.098	N	-4.033	-0.001	0	H	5.411	5.661	-0.221
C	0.431	-2.949	1.709	C	-4.737	-0.873	-0.766	H	6.086	1.69	2.704
C	-0.495	-3.675	2.466	C	-3.991	-1.811	-1.693	H	5.121	-0.089	4.164
C	-0.076	-4.408	3.588	C	-3.992	1.809	1.693	H	2.71	-0.116	4.672
C	1.272	-4.405	3.95	O	-4.532	-2.231	-2.703	H	1.195	1.62	3.725
C	2.209	-3.674	3.219	N	-2.689	-2.152	-1.315	C	-7.278	3.709	-0.93
C	2.207	3.675	-3.22	O	-4.533	2.229	2.702	Si	-9.058	3.773	-0.568
C	1.269	4.406	-3.951	N	-2.69	2.151	1.314	C	-9.333	3.118	1.157
C	-0.079	4.408	-3.589	C	-1.785	-2.94	-2.099	C	-9.609	5.548	-0.689
C	-0.497	3.675	-2.467	C	-1.786	2.94	2.098	C	-9.944	2.718	-1.824
C	0.428	2.949	-1.71	C	-0.429	-2.949	-1.71	C	-7.275	-3.71	0.931
C	-1.936	-3.664	2.105	C	0.497	-3.674	-2.467	Si	-9.056	-3.774	0.569
C	-2.776	-2.667	2.643	C	0.079	-4.407	-3.59	C	-9.33	-3.125	-1.158
C	-4.175	-2.679	2.343	C	-1.269	-4.404	-3.953	C	-9.608	-5.549	0.697
C	-4.7	-3.675	1.485	C	-2.207	-3.674	-3.221	C	-9.941	-2.714	1.821
C	-3.85	-4.662	0.93	C	-2.209	3.674	3.22	Si	9.057	-3.775	-0.567
C	-2.457	-4.662	1.259	C	-1.271	4.404	3.951	C	9.944	-2.721	-1.823
C	-2.263	-1.625	3.505	C	0.077	4.408	3.589	C	9.607	-5.55	-0.687
C	-3.089	-0.677	4.029	C	0.495	3.675	2.467	C	9.332	-3.119	1.157
C	-4.495	-0.698	3.737	C	-0.43	2.949	1.71	C	7.277	-3.71	-0.929
C	-5.017	-1.663	2.93	C	1.938	-3.662	-2.106	C	7.276	3.711	0.93
C	-4.35	-5.681	0.036	C	2.778	-2.666	-2.643	Si	9.056	3.776	0.568
C	-3.523	-6.636	-0.474	C	4.177	-2.678	-2.343	C	9.606	5.551	0.691
C	-2.129	-6.652	-0.125	C	4.702	-3.674	-1.484	C	9.331	3.123	-1.157
C	-1.618	-5.704	0.709	C	3.851	-4.661	-0.93	C	9.943	2.72	1.823
C	-1.938	3.663	-2.105	C	2.458	-4.661	-1.259	H	-8.693	3.646	1.882
C	-2.459	4.661	-1.258	C	2.266	-1.624	-3.505	H	-9.112	2.044	1.232
C	-3.852	4.66	-0.929	C	3.091	-0.676	-4.028	H	-10.379	3.266	1.467
C	-4.702	3.674	-1.484	C	4.498	-0.696	-3.736	H	-9.089	6.175	0.052
C	-4.178	2.678	-2.343	C	5.02	-1.662	-2.929	H	-10.69	5.648	-0.516
C	-2.778	2.666	-2.643	C	4.351	-5.68	-0.036	H	-9.389	5.961	-1.687
C	-1.621	5.702	-0.708	C	3.524	-6.636	0.473	H	-9.596	1.675	-1.798
C	-2.132	6.65	0.126	C	2.13	-6.651	0.124	H	-9.773	3.102	-2.842
C	-3.526	6.635	0.475	C	1.62	-5.703	-0.71	H	-11.03	2.716	-1.645
C	-4.353	5.679	-0.035	C	1.936	3.663	2.105	H	-10.375	-3.277	-1.469
C	-5.02	1.661	-2.929	C	2.457	4.662	1.259	H	-8.688	-3.654	-1.881
C	-4.497	0.696	-3.737	C	3.85	4.661	0.93	H	-9.111	-2.051	-1.237
C	-3.091	0.676	-4.029	C	4.7	3.675	1.484	H	-10.689	-5.648	0.523
C	-2.265	1.625	-3.505	C	4.176	2.679	2.343	H	-9.388	-5.958	1.695
H	6.66	-1.646	1.413	C	2.776	2.667	2.643	H	-9.089	-6.179	-0.043
H	7.943	0.002	-0.001	C	1.618	5.703	0.709	H	-9.77	-3.095	2.841
H	6.659	1.65	-1.415	C	2.129	6.651	-0.125	H	-11.027	-2.711	1.642
H	2.32	-1.768	0.434	C	3.523	6.636	-0.474	H	-9.591	-1.672	1.793
H	2.319	1.768	-0.435	C	4.35	5.681	0.036	H	11.03	-2.719	-1.644
H	0.098	-2.404	0.824	C	5.018	1.663	2.929	H	9.596	-1.678	-1.799
H	-0.803	-4.972	4.17	C	4.497	0.697	3.737	H	9.774	-3.106	-2.841
H	1.601	-4.977	4.819	C	3.09	0.677	4.028	H	9.087	-6.177	0.055
H	3.262	-3.673	3.519	C	2.264	1.625	3.505	H	10.688	-5.651	-0.513
H	3.259	3.674	-3.52	H	-6.659	-1.649	-1.414	H	9.387	-5.963	-1.684
H	1.597	4.978	-4.82	H	-7.943	-0.002	0	H	9.114	-2.045	1.232
H	-0.806	4.971	-4.17	H	-6.66	1.646	1.415	H	10.377	-3.269	1.469
H	0.096	2.404	-0.824	H	-2.319	-1.768	-0.435	H	8.69	-3.645	1.882
C	-6.087	-3.692	1.185	H	-2.32	1.768	0.434	H	9.087	6.179	-0.049
H	-1.194	-1.621	3.725	H	-0.097	-2.404	-0.824	H	10.688	5.652	0.518
H	-2.708	0.116	4.673	H	0.806	-4.969	-4.172	H	9.386	5.963	1.689
H	-5.12	0.089	4.164	H	-1.598	-4.975	-4.822	H	8.69	3.651	-1.882
H	-6.085	-1.69	2.705	H	-3.26	-3.672	-3.521	H	9.112	2.049	-1.234
H	-5.411	-5.661	-0.22	H	-3.261	3.672	3.52	H	10.376	3.274	-1.468
H	-3.894	-7.403	-1.151	H	-1.6	4.976	4.82	H	11.028	2.718	1.644
H	-1.501	-7.436	-0.545	H	0.804	4.971	4.171	H	9.595	1.677	1.796
H	-0.562	-5.706	0.986	H	-0.098	2.404	0.824	H	9.771	3.103	2.842

Table S7. Cartesian Coordinates (PM6) of head-to-tail dimer (**2c**)$_2$.

head-to-tail dimer (**2c**)$_2$											
C	-8.343	4.462	0.68	H	3.9	5.066	1.279	H	-1.743	-4.762	-4.375
C	-6.379	4.787	-3.904	H	3.598	3.28	-0.425	H	2.673	-6.293	0.897
C	-7.679	3.877	-0.463	H	3.925	0.34	-0.452	H	1.232	-7.368	2.618
C	-7.333	2.489	-0.417	H	0.666	0.492	-3.326	H	-1.195	-7.632	2.235
C	-7.688	1.729	0.76	H	1.986	2.046	-4.727	H	-0.303	-3.659	-6.084
C	-8.313	2.318	1.817	H	4.262	2.758	-4.015	H	2.109	-3.331	-5.666
C	-8.644	3.715	1.779	H	0.456	-0.778	-1.43	H	3.139	-4.103	-3.536
C	-5.741	4.197	-4.953	C	-4.169	1.559	2.111	H	3.061	-3.204	-0.386
C	-5.378	2.808	-4.893	C	-2.781	-2.413	4.799	H	7.128	-4.689	-0.692
C	-5.669	2.066	-3.789	C	-3.182	0.815	2.859	H	6.264	-6.775	-1.714
C	-6.349	2.653	-2.654	C	-1.865	1.358	2.986	H	3.822	-7.087	-2.091
C	-6.707	4.038	-2.713	C	-1.57	2.611	2.328	H	4.809	-1.927	0.414
C	-7.364	4.64	-1.613	C	-2.525	3.276	1.619	C	8.378	-0.232	0.807
C	-6.659	1.899	-1.506	C	-3.855	2.743	1.514	H	9.159	-0.745	0.238
C	-6.26	0.472	-1.419	C	-1.824	-3.081	5.503	C	-7.709	6.016	-1.663
C	-5.032	0.145	-0.836	C	-0.512	-2.517	5.657	C	-8.006	7.193	-1.705
C	-4.679	-1.211	-0.681	C	-0.209	-1.313	5.096	C	-4.824	-0.912	3.419
C	-5.543	-2.224	-1.127	C	-1.191	-0.574	4.333	C	-5.975	-1.3	3.381
C	-6.75	-1.875	-1.735	C	-2.502	-1.133	4.19	C	-2.309	-5.899	-2.209
C	-7.119	-0.536	-1.883	C	-3.496	-0.417	3.482	C	-3.495	-6.075	-2.405
N	-3.414	-1.487	-0.069	C	-0.896	0.676	3.753	C	9.472	3.446	-0.813
C	-2.955	-2.752	0.316	C	0.416	1.322	4.006	C	10.583	3.851	-0.534
O	-3.617	-3.769	0.233	C	1.566	0.922	3.32	Si	-5.294	-6.277	-2.59
C	-1.551	-2.774	0.893	C	2.792	1.568	3.589	Si	-7.718	-1.817	3.283
N	-0.62	-1.96	0.34	C	2.854	2.603	4.539	Si	12.246	4.464	-0.122
C	1.022	-2.882	1.875	C	1.696	2.983	5.217	Si	-8.453	8.956	-1.766
C	0.045	-3.712	2.436	C	0.477	2.353	4.96	C	12.881	3.477	1.325
C	-1.26	-3.67	1.941	N	3.941	1.119	2.865	H	12.227	3.59	2.204
C	9.165	1.288	-2.693	C	5.239	1.606	3.044	H	12.939	2.405	1.084
C	7.165	4.595	0.47	O	5.519	2.516	3.807	H	13.889	3.81	1.615
C	8.024	1.911	-2.062	C	6.333	0.953	2.223	C	13.335	4.226	-1.614
C	6.714	1.434	-2.387	N	6.099	-0.254	1.651	H	12.934	4.769	-2.485
C	6.593	0.336	-3.321	C	8.599	1.014	1.4	H	14.354	4.598	-1.428
C	7.694	-0.232	-3.886	C	7.569	1.621	2.122	H	13.411	3.163	-1.889
C	9.007	0.257	-3.57	C	-1.159	-6.736	0.291	C	12.105	6.268	0.317
C	6.067	5.116	1.085	C	-0.675	-4.611	-4.203	H	11.682	6.846	-0.52
C	4.754	4.631	0.761	C	-0.358	-6.122	-0.743	H	11.45	6.417	1.189
C	4.593	3.649	-0.169	C	1.043	-5.947	-0.512	H	13.088	6.699	0.558
C	5.732	3.059	-0.838	C	1.601	-6.417	0.737	C	-9.319	9.386	-0.173
C	7.04	3.54	-0.51	C	0.813	-7.003	1.682	H	-9.614	10.446	-0.155
C	8.174	2.969	-1.134	C	-0.597	-7.162	1.457	H	-10.229	8.781	-0.04
C	5.584	2.03	-1.79	C	0.109	-4.006	-5.139	H	-8.666	9.205	0.695
C	4.225	1.59	-2.195	C	1.512	-3.817	-4.895	C	-6.886	9.943	-1.955
C	3.493	0.709	-1.387	C	2.073	-4.24	-3.728	H	-6.2	9.76	-1.113
C	2.209	0.319	-1.795	C	1.277	-4.886	-2.708	H	-6.355	9.674	-2.882
C	1.66	0.797	-3.004	C	-0.121	-5.072	-2.95	H	-7.095	11.023	-1.989
C	2.406	1.671	-3.793	C	-0.926	-5.697	-1.968	C	-9.582	9.215	-3.224
C	3.684	2.072	-3.397	C	1.843	-5.334	-1.497	H	-9.896	10.267	-3.304
N	1.384	-0.569	-1.026	C	3.298	-5.16	-1.262	H	-9.085	8.939	-4.166
C	1.701	-1.131	0.205	C	3.765	-3.98	-0.675	H	-10.491	8.599	-3.134
O	2.777	-0.959	0.77	C	5.149	-3.806	-0.472	C	-5.871	-5.131	-3.943
C	0.648	-2.025	0.825	C	6.051	-4.818	-0.848	H	-5.376	-5.363	-4.897
H	-8.597	5.523	0.635	C	5.563	-5.991	-1.425	H	-6.957	-5.21	-4.099
H	-6.66	5.842	-3.938	C	4.194	-6.172	-1.637	H	-5.639	-4.083	-3.691
H	-7.454	0.656	0.778	N	5.567	-2.571	0.117	C	-6.047	-5.806	-0.955
H	-8.576	1.738	2.71	C	6.895	-2.2	0.346	H	-5.592	-4.851	-0.603
H	-9.14	4.154	2.643	O	7.851	-2.902	0.062	H	-7.134	-5.672	-1.005
H	-5.493	4.757	-5.853	C	7.112	-0.831	0.958	H	-5.831	-6.561	-0.183
H	-4.869	2.37	-5.75	H	-5.18	1.148	2.04	C	-5.653	-8.052	-3.028
H	-5.4	1.011	-3.733	H	-3.786	-2.826	4.682	H	-5.185	-8.325	-3.986
H	-4.369	0.94	-0.496	H	-0.558	3.01	2.415	H	-5.26	-8.735	-2.259
H	-5.28	-3.286	-0.992	H	-2.307	4.221	1.122	H	-6.735	-8.23	-3.116
H	-7.414	-2.664	-2.092	H	-4.601	3.308	0.953	C	-8.163	-1.889	1.477
H	-8.066	-0.272	-2.348	H	-2.032	-4.042	5.969	H	-7.55	-2.642	0.953
H	-2.806	-0.673	0.094	H	0.224	-3.071	6.237	H	-9.22	-2.145	1.322
H	2.054	-2.89	2.236	H	0.783	-0.873	5.215	H	-7.973	-0.917	0.98
H	0.303	-4.392	3.25	H	1.513	0.119	2.586	C	-8.74	-0.528	4.163
H	-2.044	-4.318	2.341	H	3.803	3.108	4.752	H	-9.803	-0.809	4.181
H	10.157	1.669	-2.447	H	1.747	3.783	5.957	H	-8.406	-0.403	5.205
H	8.166	4.962	0.702	H	-0.422	2.654	5.492	H	-8.658	0.456	3.663
H	5.592	-0.031	-3.554	H	3.784	0.343	2.192	C	-7.896	-3.483	4.097
H	7.607	-1.065	-4.583	H	9.565	1.515	1.29	H	-8.935	-3.842	4.049
H	9.864	-0.219	-4.044	H	7.7	2.595	2.603	H	-7.259	-4.231	3.599
H	6.156	5.902	1.834	H	-2.232	-6.844	0.109	H	-7.603	-3.443	5.157
